# Transcriptomic Analysis of Differentially Expressed Genes During Larval Development of *Rapana venosa* by Digital Gene Expression Profiling

**DOI:** 10.1534/g3.116.029314

**Published:** 2016-05-18

**Authors:** Hao Song, Zheng-Lin Yu, Li-Na Sun, Dong-Xiu Xue, Tao Zhang, Hai-Yan Wang

**Affiliations:** * Key Laboratory of Marine Ecology and Environmental Sciences, Institute of Oceanology, Chinese Academy of Sciences, Qingdao 266071, People’s Republic of China; † University of Chinese Academy of Sciences, Beijing 100049, People’s Republic of China

**Keywords:** transcriptome, *Rapana venosa*, gastropod, larva, digital gene expression

## Abstract

During the life cycle of shellfish, larval development, especially metamorphosis, has a vital influence on the dynamics, distribution, and recruitment of natural populations, as well as seed breeding. *Rapana venosa*, a carnivorous gastropod, is an important commercial shellfish in China, and is an ecological invader in the United States, Argentina, and France. However, information about the mechanism of its early development is still limited, because research in this area has long suffered from a lack of genomic resources. In this study, 15 digital gene expression (DGE) libraries from five developmental stages of *R. venosa* were constructed and sequenced on the IIIumina Hi-Sequation 2500 platform. Bioinformaticsanalysis identified numerous differentially and specifically expressed genes, which revealed that genes associated with growth, nervous system, digestive system, immune system, and apoptosis participate in important developmental processes. The functional analysis of differentially expressed genes was further implemented by gene ontology, and Kyoto encyclopedia of genes and genomes enrichment. DGE profiling provided a general picture of the transcriptomic activities during the early development of *R. venosa*, which may provide interesting hints for further study. Our data represent the first comparative transcriptomic information available for the early development of *R. venosa*, which is a prerequisite for a better understanding of the physiological traits controlling development.

*Rapana venosa* is a species of large predatory sea snail that is naturally distributed in the western Pacific, the Sea of Japan, the Yellow Sea, the Bohai Sea, and the East China Sea (Savini *et al.* 2004). Since Valenciennes first reported that *R. venosa* had invaded the northern Adriatic Sea in 1846, it has been established in many different localities around the world in the last few decades. *R. venosa* is found in countries such as the United States, Argentina, and France (Mann and Harding 2003; Mann *et al.* 2006; Giberto *et al.* 2006; Leppäkoski *et al.* 2013) as a biological invader because of its ecological flexibility, and its tolerance to low salinity, low oxygen and significantly polluted water (Giberto *et al.* 2006; Çulha *et al.* 2009). It feeds at night, and is often found in locations that are hard to sample, making early detection of newly established populations difficult (Harding *et al.* 2007a). *R. venosa* invasion can have a seriously negative impact on the environment. As *R. venosa* became more prevalent in the Chesapeake Bay region (VA), Harding *et al.* (2007a) found significant differences in predation strategies and prey species consumed between *R. venosa* and the native gastropod *Urosalpinx cinerea*, which could cause considerable disruption of the trophic structure (feeding relationships among species) in Chesapeake Bay. However, *R. venosa* is considered as an important commercial gastropod species in China (Liu *et al.* 2003), and, as a result of intensive research, the industrial aquaculture of *R. venosa* remains efficient. In recent years, research has been conducted on its reproductive biology (Harding *et al.* 2007b; Yang *et al.* 2007), invasion biology (Leppäkoski *et al.* 2013; Harding *et al.* 2008; Lanfranconi *et al.* 2009), and population genetics (Xue *et al.* 2014; Sun *et al.* 2014). However, information related to genomic and transcriptome resources, and the mechanism of development of *R. venosa*, are currently unavailable.

The veliger is a critical life cycle stage of the gastropod, bivalve, and scaphopod taxonomic classes. With the development of veliger larvae, larvae become competent for settlement and metamorphosis, which link free-swimming larva and benthic juveniles. Metamorphosis is a dramatic and irreversible developmental event involving physical, physiological, and behavioral transformations (Leise *et al.* 2009). Studies on the mechanism of mollusk early development can provide useful information for aquaculture, resource restoration and bio-antifouling. Studies on the mechanism of larval development, especially metamorphosis, have made great progress in recent years. Some genes and proteins involved in these events have been identified and characterized functionally. *Hox* and *paraHox* genes with different sequences and gene expression patterns in mollusks are considered to play an important role in shell formation, body plan evolution, and diversification (Biscotti *et al.* 2014). Nitric oxide synthase (NOS) and Hsp90 are required for metamorphosis of the mollusk *Haliotis asinina*: their RNA expression levels are upregulated at the initiation of settlement and metamorphosis, and their expression levels show marked differences between competent and precompetent larval stages (Ueda 2013). mRNA differential display of gene expression in the settlement metamorphosis process was performed in *Ruditapes philippinarum* to elucidate the potential mechanisms underlying larval development and metamorphosis (Lu *et al.* 2008). However, research in this field has been hampered by a long-term lack of genomic resources, especially useful transcriptome sequences.

Recently, next-generation sequencing (NGS) technologies have become the main method for genome-wide characterization and profiling of mRNA (Ansorge 2009). NGS offers an effective approach to obtaining sequence information for nonmodel species in which genomic sequences are limited. Deep-sequenced transcriptomes of several mollusks, such as oysters *Crassostrea virginica* (Quilang *et al.* 2007), *Crassostrea gigas* (Fleury *et al.* 2009), the abalone *Haliotis midae* (Franchini *et al.* 2011), the scallop *Patinopecten yessoensis* (Hou *et al.* 2011), the Antarctic bivalve *Laternula elliptica* (Clark *et al.* 2010), and the mussel *Mytilus galloprovincialis* (Craft *et al.* 2010) have been published recently. However, in mollusks, very few transcriptome sequences have been reported for their early developmental stages, especially for planktonic larvae. Digital gene expression (DGE) can effectively and efficiently detect the differential and specific gene expression of a specific organization in certain conditions using NGS technology and high performance computing analysis. DGE has been used in such fields as basic scientific research, medical research, and drug development. In mollusks, DGE was applied to detect immune responses to vibrio infection in the oyster *C. gigas* (De Lorgeril *et al.* 2011); DGE studies of molluskan development are still limited.

In this study, our objective was to establish a useful database of differentially expressed genes (DEGs) from different developmental stages of *R. venosa*. These data would provide a better understanding of the mechanism of veliger development. We performed a comparative analysis of the transcriptomes from five different developmental stages of *R. venosa* by using DGE. Our data should facilitate better understanding of the molecular mechanism of early development of *R. venosa* larva, and represents a valuable resource for genetic and genomic researches on *R. venosa* in the future.

## Materials and Methods

### Larva culture and sample preparation

*Rapana venosa* larvae were obtained from the Blue Ocean Co. Limited (Laizhou, Shandong, China). Parent culture, spawning, and larva rearing were performed according to the study of Yang *et al.* (2007). Planktonic larvae were cultured in 3 m × 5 m × 1.5 m cement pools at 24–26°, at a density of 0.5/ml. *Platymonas subcordiformis*, *Isochrysis galbana*, and *Chlorella vulgaris* were mixed as a diet (15 × 104 cell/ml, three or four times) for the Planktonic larvae. Larval samples from five major developmental stages were collected: the one-spiral whorl stage, the two-spiral whorls stage, the three-spiral whorls stage, the four-spiral whorls stage (competent larva), and the postlarval stage. The samples were monitored under a microscope to ensure developmental synchrony. Each sample was then washed with ddH_2_O, snap-frozen in liquid nitrogen, and stored at −80° until use.

### RNA extraction, library preparation, and Hi-sequation 2500 sequencing

Each stage had three replicates for RNA extraction, and each replicate contained approximately 500 mg of larvae. We carried out RNA extraction following the instructions of the TRIzol Kit (Invitrogen, CA). RNA quality was examined by electrophoresis through 1.2% agarose gels to check for possible degradation and contamination. RNA purity was monitored on a NanoPhotometer spectrometer (IMPLEN, CA) using default parameters. Integrity and concentration and of RNA was measured using a Qubit RNA Assay Kit in a Qubit 2.0 Flurometer (Life Technologies, CA), and the RNA 6000 Nano Assay Kit of the Bioanalyzer 2100 system (Agilent Technologies, CA), respectively. Fifteen RNA samples (five stages × three replicates) that satisfied all requirements of the quality test were selected for library preparation.

RNA (3 μg per sample) was used as the input material for the RNA sample preparations. Sequencing libraries were constructed according to the manufacturer’s instructions using a NEBNext Ultra Directional RNA Library Prep Kit for Illumina (NEB). After clustering of the index-coded samples, we performed RNA sequencing of each library on an Illumina HiSequation 2500 platform to generate 100-bp single-end (SE) reads. This procedure was carried out by Novogene Bioinformatics Technology Co. Ltd (Tianjing, China).

### Mapping and analysis of DGE reads

The raw data were deposited in the gene expression omnibus (GEO) database of NCBI with the accession number GSE70548. Raw reads were processed into clean reads by removing adapters, poly-N regions, and low quality reads. Moreover, the Q20, Q30, GC-content, and sequence duplication level of the clean data were calculated. The high quality clean data were aligned to the *R. venosa* reference transcriptome using RSEM v1.2.15 (Li and Dewey 2011) (the reference transcriptome was *de novo* sequenced and assembled in our previous work, based on a mixture of RNAs from different developmental larval stages, the raw data of which is available in the SRA with the accession number SRR2086477; the annotation has been deposited at DDBJ/EMBL/GenBank under accession no. GDIA00000000 (Song *et al.* 2016). To estimate the gene expression level, RSEM analysis was performed to obtain the readcount for each gene of each sample, based on the mapping results. Then readcount was converted into the number of fragments per kilobase per million (FPKM value) (Mortazavi *et al.* 2008). Differential expression analysis between five developmental stages was implemented using the DESeq R package v1.12.0 (Anders and Huber 2010). The P-value was adjusted using the q-value (or padj) (Anders and Huber 2010). There were three replicates of each samples; therefore, a q-value < 0.05 was set as the threshold to select genes with significant differential expression (Tan *et al.* 2015).

### Functional analysis of differentially expressed genes

Gene ontology (GO) enrichment analysis of DEGs was performed using GOseq v1.10.0, which can eliminate gene length bias in DEGs. The KEGG is a database resource used to aid understanding of the high-level functions and uses of biological systems (http://www.genome.jp/kegg/). Here, we used KOBAS software, version 2.0.12, to analyze the statistical enrichment of DEGs in KEGG pathways.

Based on the normalized, filtered sequences, cluster analysis of gene expression was implemented to identify genes of which the expression level were remarkably different among this five developmental stages of *R. venosa*. To ensure a more persuasive result, we chose those annotated genes with an average FPKM in all stages of more than 10, a q-value < 0.001 and |log_2_(foldchange)| > 2 in certain comparisons of two stages as candidates (1794 genes) for analysis using Cluster 3.0 software.

### Quantitative real-time reverse transcription PCR

To validate the mRNA expression levels indicated by the DGE analysis, 20 annotated DEGs (including the 10 most upregulated and 10 most downregulated DEGs) from the comparison of competent *vs.* post larva stages were selected. To make the result more reliable, genes without annotation or annotated with “hypothetical protein” were filtered out, and genes with an FPKM < 1 in these two stages were also filtered out. The DGE input RNA was used as the template for cDNA synthesis, following the instructions of the PrimeScript RT reagent Kit with gDNA Eraser (Takara, Japan). The quantitative analysis of mRNA expression levels by quantitative real-time reverse transcription PCR (qRT-PCR) was implemented using a SYBR PrimeScript RT-PCR Kit II (Takara, Japan), according to the instruction manual, in an Eppendorf Mastercycler ep realplex apparatus (Eppendorf, Hamburg, Germany). GAPDH was chosen as an internal reference gene to normalize the data (Badariotti *et al.* 2007; Qian *et al.* 2012), and three replicates were performed. The primers used (Supplemental Material, Table S2) were designed using Primer3 (http://primer3.sour-ceforge.net/r-eleases.php). The PCR reaction volumes comprised 10 × PCR buffer, 2.5 μl; dNTPs, 2 μl (2.5 mmol/l); forward primer, 1 μl (5 μmol/l); reverse primer, 1 μl (5 μmol/l); Ex Taq (TAKARA), 0.2 μl; cDNA template, 0.1 μl (containing 1 ng of cDNA); and ddH_2_O up to 25 μl. The PCR conditions were set as follows: 95° for 3 min; 95° for 15 sec, 60° for 25 sec, 72° for 15 sec for 40 cycles; 72° for 5 min, with a final 10-min extension step at 72°. The PCR products were detected by electrophoresis through a 1.2% agarose gel. The relative expression level was calculated by the 2^–△△Ct^ method. PASW Statistics 18 (SPSS Inc.) was used for statistical analysis. Significance was tested by one-way ANOVA using Tukey’s test. *P* < 0.05 was considered significant.

### Data availability

The authors state that all data necessary for confirming the conclusions presented in the article are represented fully within the article.

## Results

### General characteristics of the 15 DGE libraries

**Figure 1:**
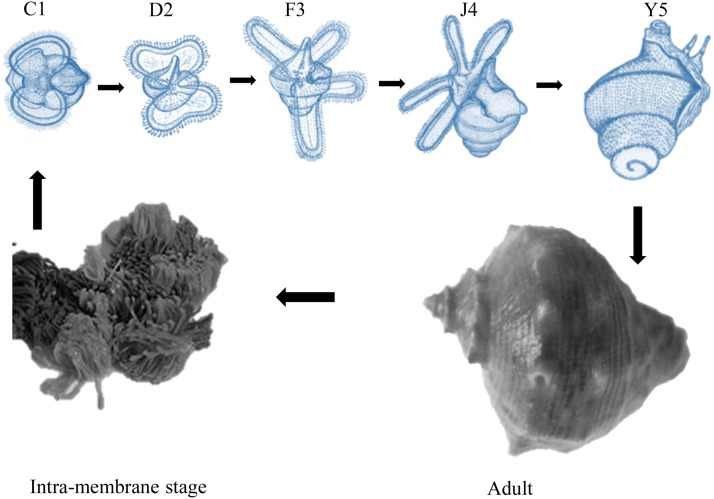
Five developmental stages of *R. venosa* that were sampled for transcriptome analysis (shown in blue). The one-spiral whorl stage (C1), the two-spiral whorls stage (D2), the three-spiral whorls stage (F3), the four-spiral whorls stage (competent larva, J4), and the postlarval stage (Y5).

To explore the genes and their networks which control the development of larva in *R. venosa*, we determined and compared the transcriptome of five larval stages (C1: the one-spiral whorl stage, D2: the two-spiral whorls stage, F3: the three-spiral whorls stage, J4: the four-spiral whorls stage, and Y5: the postlarval stage). As shown in Figure 1, there are visible morphological and body size differences between these five stages (Pan *et al.* 2013).

For each sample, the high-throughput RNA sequencing obtained 10.51–15.57 million single-ended reads (Table 1). After filtering for low quality reads and adapter sequences, 10.30–15.17 million clean reads (99.26–96.05% from raw data) were generated. Among the clean data of the 15 libraries, about 82.37–87.34% of clean sequences was successfully mapped to the entire reference transcriptome (Table 1).

**Table 1:** Statistics of DGE sequencing

Sample	Raw Reads	Clean Reads	Clean Bases (G)	Error (%)	Q20 (%)	Q30 (%)	GC Content (%)	Total Mapped (% of Clean Data)
C1_1	10,985,377	10,893,487	0.54	0.01	98.47	97.02	47.89	9,428,310 (86.55%)
C1_2	13,169,590	12,998,744	0.65	0.01	98.48	97.04	46.89	11,153,576 (85.81%)
C1_3	12,218,112	11,735,699	0.59	0.01	98.48	97.04	47.58	10,123,873 (86.27%)
D2_1	15,568,814	15,171,768	0.76	0.01	98.59	97.16	48.73	13,191,966 (86.95%)
D2_2	12,006,329	11,727,213	0.59	0.01	98.49	96.97	48.71	10,186,346 (86.86%)
D2_3	14,569,020	14,161,517	0.71	0.01	98.59	97.17	48.91	12,368,256 (87.34%)
F3_1	13,122,713	12,689,128	0.63	0.01	98.08	95.99	48.99	11,044,387 (87.04%)
F3_2	10,972,646	10,832,113	0.54	0.01	98.11	96.04	48.37	9,341,753 (86.24%)
F3_3	12,235,201	12,145,123	0.61	0.01	98.12	96.06	48.32	10,514,719 (86.58%)
G3_1	10,508,213	10,302,115	0.52	0.01	97.91	95.4	47.9	8,708,196 (84.53%)
G3_2	11,352,406	11,225,014	0.56	0.01	97.91	95.38	46.38	9,245,843 (82.37%)
G3_3	12,068,623	11,907,135	0.6	0.01	97.89	95.34	47.03	9,846,087 (82.69%)
J4_1	14,827,351	14,665,024	0.73	0.01	98.14	96.07	46.76	12,283,484 (83.76%)
J4_2	12,026,881	11,908,433	0.6	0.01	98.14	96.07	46.57	9,996,051 (83.94%)
J4_3	12,376,019	12,118,735	0.61	0.01	98.14	96.08	47.26	10,146,937 (83.73%)
Y5_1	13,520,738	13,375,042	0.67	0.01	98.27	96.53	46.72	11,220,158 (83.89%)
Y5_2	12,988,065	12,634,083	0.63	0.01	98.22	96.45	47	10,615,299 (84.02%)
Y5_3	12,780,985	12,628,348	0.63	0.01	98.24	96.48	47.27	10,597,512 (83.92%)

**Figure 2:**
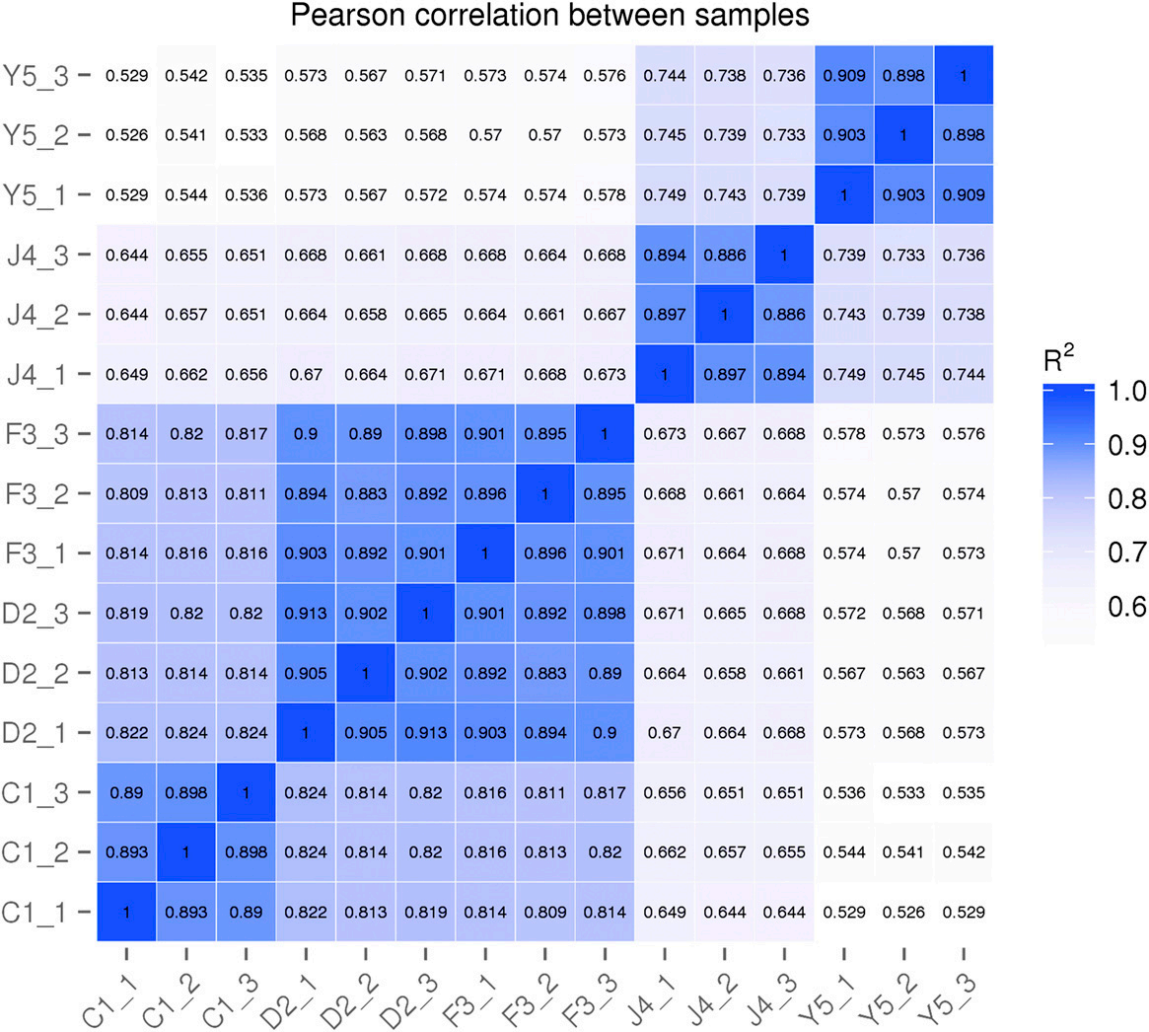
Pearson correlation coefficients between samples.

The Pearson correlation coefficients between each DGE library are presented in Figure 2. The Pearson correlation coefficients between replicates remained high, with an average coefficient of *r* = 0.894 for C1, *r* = 0.917 for D2, *r* = 0.897 for F3, *r* = 0.892 for J4, and *r* = 0.903 for Y5, which indicates the satisfactory reproducibility of the biological replicates. A volcano plot showing the number and relationship between fold-change and p-value of up/downregulated DEGs in each comparison can be found in Figure S1.

### Development dynamics of differential gene expressions

**Figure 3:**
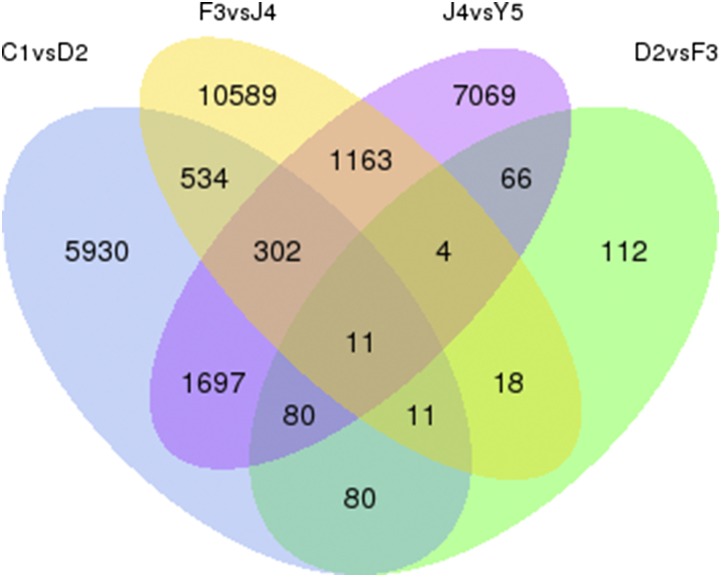
Venn diagram of differentially expressed genes. The sum of numbers in each big circle is the total number of differentially expressed genes in this comparison group, and the overlapping part is the number of common differentially expressed genes among the comparison groups.

A Venn diagram was drawn to illustrate the number of common and unique DEGs among the five stages (Figure 3). There were 11 common DEGs among the comparison groups, and 7069 specific DEGs in the J4 *vs.* Y5 comparison.

While comprehensive analysis of the expression level of these sequences still requires much work, a preliminary analysis revealed that some genes showed a rather different expression pattern among different larval stages (Table S3). Some of these data are shown in Table 2. The expression patterns of these genes varied widely. Different genes were abundantly expressed mainly in the precompetent larval stage (*e.g.*, SARP-19), competent larval stage (actin, and sulfotransferase 1A2 and 1C2), or expressed in the postlarval stage (*e.g.*, trypsin and defensin). Their functions were related to growth and development, immunity, signal transduction, *etc*. This information brings indications revealing their function.

**Table 2:** Some of the genes that were differentially expressed among the five developmental stages of *R. venosa*

	FPKM					
Gene_id	C1	D2	C3	J4	Y5	NR/Swissprot Description
Growth related genes						
c145901_g1	28.17 ± 4.11	4.89 ± 0.20	4.83 ± 0.74	1.63 ± 0.17	1.13 ± 0.38	Growth/differentiation factor 8
c137141_g1	1.58 ± 0.77	0.11 ± 0.19	0.10 ± 0.18	51.52 ± 6.43	145.47 ± 9.43	Fibropellin
c100045_g1	1.64 ± 0.16	0.65 ± 0.06	0.74 ± 0.41	0.17 ± 0.02	0.34 ± 0.07	Nodal
c145993_g2	3.98 ± 1.76	0.97 ± 0.60	0.32 ± 0.56	50.06 ± 2.90	1.41 ± 0.04	Actin
c146789_g2	9.02 ± 0.17	5.68 ± 0.64	5.68 ± 0.04	13.56 ± 0.67	13.01 ± 0.95	Epidermal growth factor receptor
Nervous system related genes						
c134668_g1	4.14 ± 0.32	2.12 ± 0.26	1.78 ± 0.24	2.32 ± 0.39	0.89 ± 0.29	5-Hydroxytryptamine receptor 1
c138434_g1	2.58 ± 0.42	3.09 ± 0.08	3.44 ± 0.63	0.28 ± 0.20	0.00 ± 0.00	Neuronal acetylcholine receptor
c156806_g2	4.76 ± 0.11	3.67 ± 0.14	3.39 ± 0.51	2.45 ± 0.04	1.22 ± 0.36	Nitric oxide synthase
c90120_g1	71.07 ± 2.83	50.29 ± 1.91	49.18 ± 1.55	46.61 ± 1.98	101.93 ± 4.53	GABA(A) receptor-associated protein
Digestive system related genes						
c124801_g1	2083.42 ± 193.38	8288.19 ± 320.86	9144.35 ± 631.79	2226.16 ± 100.74	0.13 ± 0.23	Conotoxin
c150903_g1	65.50 ± 5.18	70.21 ± 3.19	69.11 ± 7.10	2.64 ± 0.34	1.59 ± 0.09	Exoglucanase XynX
c154739_g1	35.47 ± 1.81	526.05 ± 13.37	595.43 ± 28.38	22.31 ± 0.91	0.05 ± 0.01	Endoglucanase E-4
c95470_g1	3989.92 ± 168.41	11536.75 ± 123.56	13857.77 ± 358.89	2519.76 ± 154.86	3.09 ± 1.45	vdg3
c105120_g1	1.95 ± 0.37	12.40 ± 0.24	17.84 ± 3.84	1695.11 ± 50.76	6247.54 ± 525.34	Developmentally-regulated vdg3
c128291_g1	0.04 ± 0.03	0.02 ± 0.03	0.06 ± 0.06	29.25 ± 1.51	221.63 ± 15.27	Trypsin
c147105_g1	0.30 ± 0.26	0.10 ± 0.10	0.00 ± 0.00	91.37 ± 7.91	187.18 ± 25.41	Carboxypeptidase B
Immune system related genes						
c115222_g1	4.27 ± 1.29	12.00 ± 0.68	12.61 ± 0.66	6.71 ± 0.37	36.95 ± 0.15	Tumor Necrosis Factor
c137778_g1	0.62 ± 0.27	0.95 ± 0.17	1.35 ± 0.78	4.70 ± 0.98	163.68 ± 6.87	Defensin
c149483_g1	3.29 ± 0.34	10.01 ± 2.01	10.32 ± 1.35	5.20 ± 0.89	24.37 ± 4.74	toll2
Apoptosis related genes						
c132048_g1	22.78 ± 0.90	30.65 ± 1.10	26.18 ± 0.92	42.81 ± 2.64	14.86 ± 0.34	Apoptosis-inducing factor 1
c135194_g1	0.76 ± 0.10	0.61 ± 0.06	0.78 ± 0.10	2.54 ± 0.25	1.25 ± 0.19	Caspase-7
c147256_g2	18.34 ± 0.12	15.66 ± 0.35	15.15 ± 0.55	15.41 ± 0.52	7.53 ± 0.73	Caspase-3
c151900_g1	2.52 ± 0.69	5.06 ± 0.56	4.23 ± 0.88	4.61 ± 0.65	10.52 ± 0.62	Apoptosis 2 inhibitor
Others						
c116117_g1	7.06 ± 1.78	64.45 ± 4.37	52.10 ± 2.14	15.95	99.14 ± 9.69	Calmodulin
c125727_g1	0.49 ± 0.12	0.37 ± 0.03	0.63 ± 0.24	62.61	3.70 ± 0.06	Sulfotransferase 1C2
c141012_g1	6.10 ± 0.55	6.37 ± 0.75	6.54 ± 0.97	70.58	8.93 ± 1.33	Sulfotransferase 1A2
c112229_g1	267.04 ± 8.16	223.74 ± 0.946	252.36 ± 11.47	53.19 ± 1.99	5.86 ± 0.62	SARP-19

**Figure 4:**
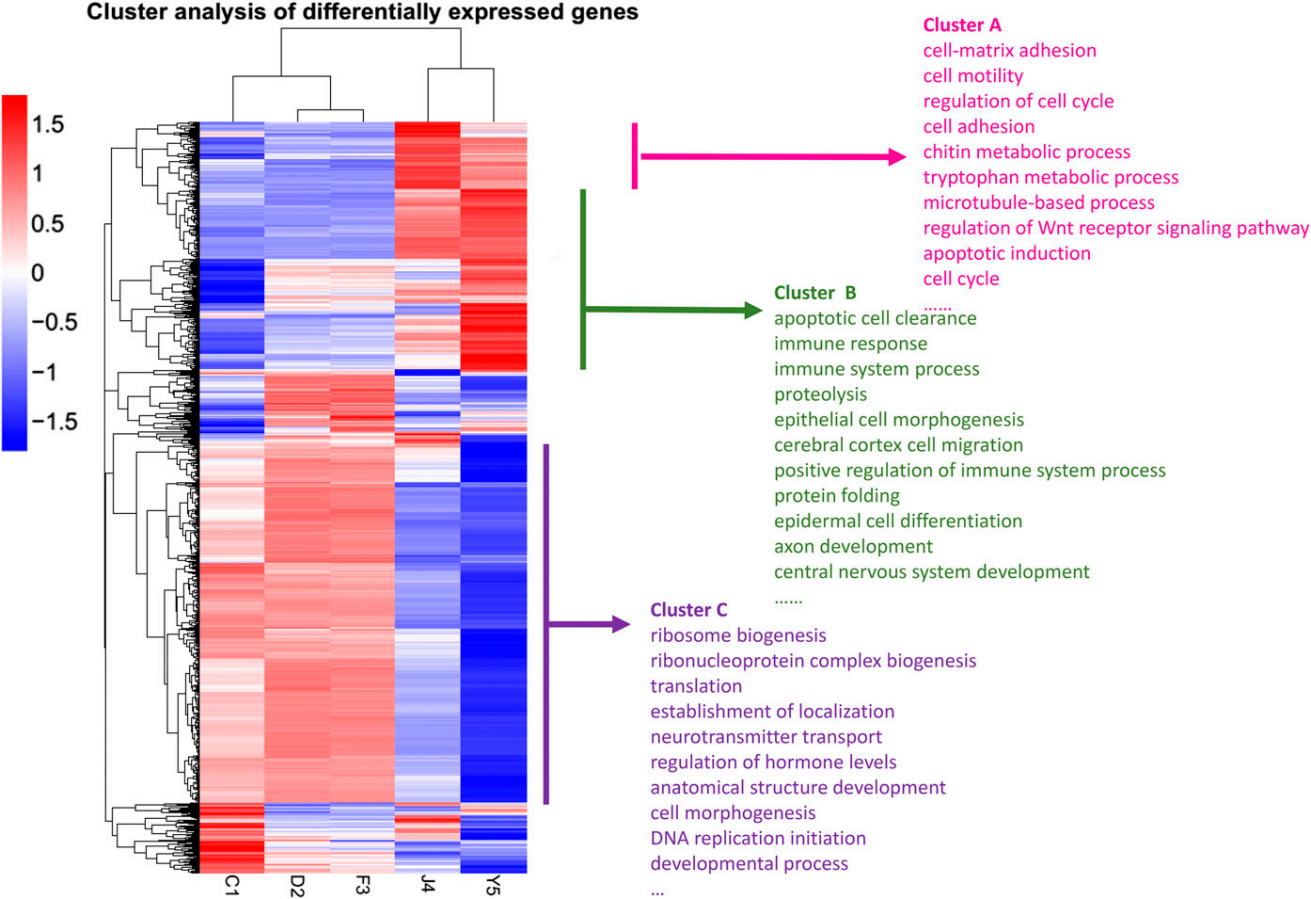
Clusters of expression levels of the candidate genes. Dendrograms showing the gene expression patterns for 1794 genes. The clustering indicates similar expression patterns among the developmental stages (*x*-axis) and among the genes (*y*-axis). The bar color reflects the gene expression level from blue (low) to white (medium) to red (high). For each cluster, some of the enriched terms of biological process in GO enrichment are listed.

To estimate expression patterns of DEGs under different developmental conditions, 1794 genes selected according to criteria detailed in *Materials and Methods* were clustered hierarchically. The expression profiles of the 1794 genes are displayed in Figure 4, and were considered reliable by strict control of the p-value and FPKM. The information from the horizontal axis of the gene clustering dendrogram indicated that the developmental stages were aggregated into two distinct clusters. The one-, two- and three-spiral whorls stages formed one cluster; and the four-spiral whorls stage (competent larva), and postlarval stage formed another. The vertical axis of the dendrogram showed that each developmental stage contained genes that were specifically and abundantly expressed. These stage-specific genes predominantly distributed in the two- or three-spiral whorls stages, four-spiral whorls stages, and postlarval stages. For example, expression of genes in cluster A remained quite low during the precompetent larval stage, but increased rapidly in the competent larval stage, and then reduced after metamorphosis. And genes were abundantly expressed in the postlarval stage in cluster B. In cluster C, genes were abundantly expressed during the precompetent larval stage (one-, two- and three-spiral whorl stage), but decreased significantly in the following developmental stages (four-spiral whorl stage, and postlarval stage). More detailed information on these genes of different clusters is available in Table S1. This analysis of gene expression patterns will help discover genes playing a pivotal role in controlling early developmental processes in *R. venosa*.

### GO enrichment and KEGG enrichment of upregulated DEGs after metamorphosis

**Figure 5:**
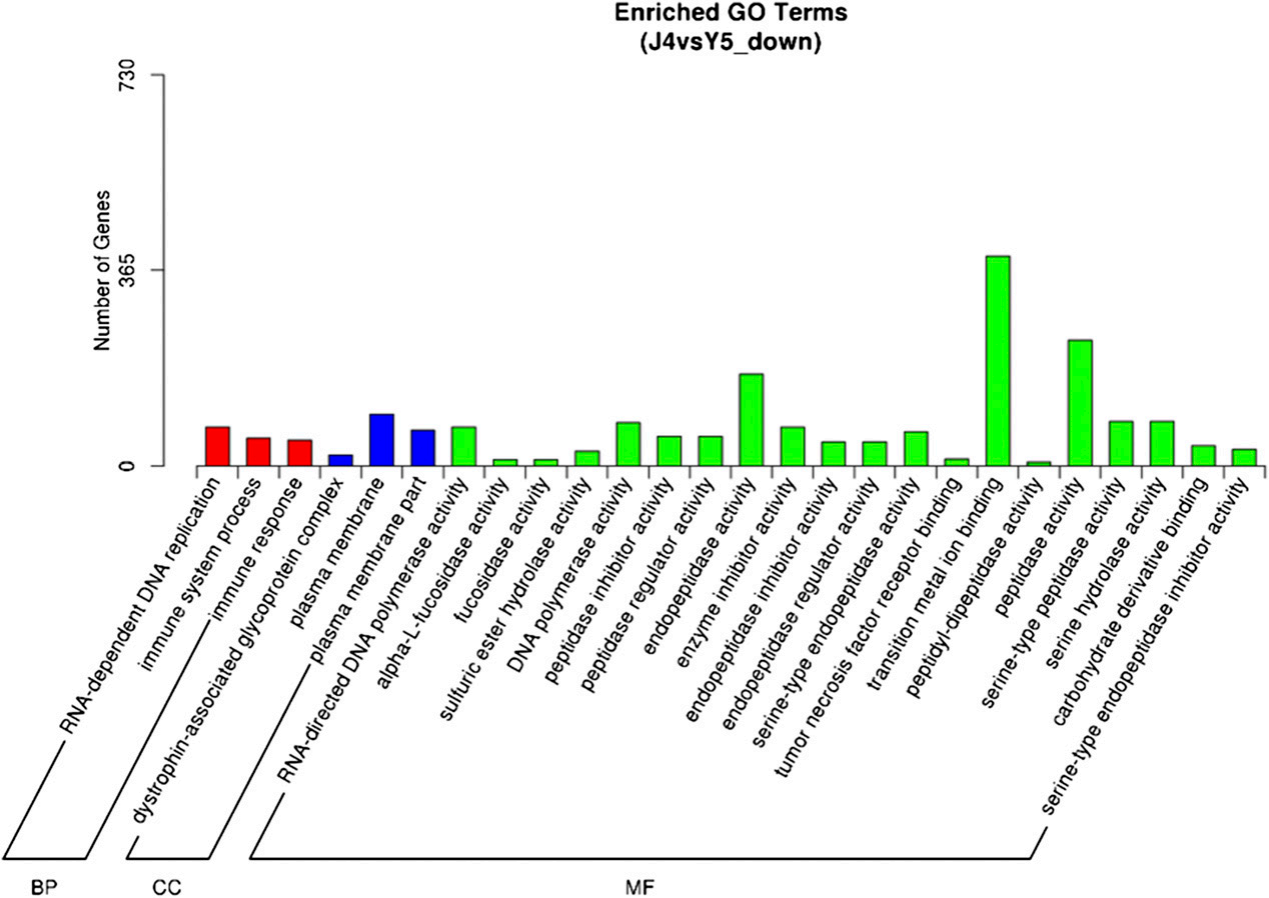
Bar graph of GO enrichment of genes that were upregulated after metamorphosis. The horizontal axis shows the GO term in the next level of the three main GO classifications; the vertical axis shows the number of the differentially expressed genes annotated in the GO term, and the total number of annotated differentially expressed genes. The three main GO classifications are (from left to right): biological process, cell composition, and molecular function.

To further understand the function of DEGs, GO term enrichment analysis, and Kyoto Encyclopedia of Genes and Genomes (KEGG) enrichment were performed. We focused mainly on the DEGs of the J4 and Y5 comparison, because metamorphosis, which involves velum degeneration, diet transformation, growth of the secondary shell, and other morphological and ecological habit changes, is the critical point of the *R. venosa* life cycle. For genes that were upregulated in Y5, “RNA-dependent DNA replication” (72 DEGs), “immune system process” (51 DEGs), and “immune response” were most enriched in the biological process (GOBP) group (red in Figure 5). GO enrichment analysis of the other developmental comparison groups is shown in Figure S2.

**Figure 6:**
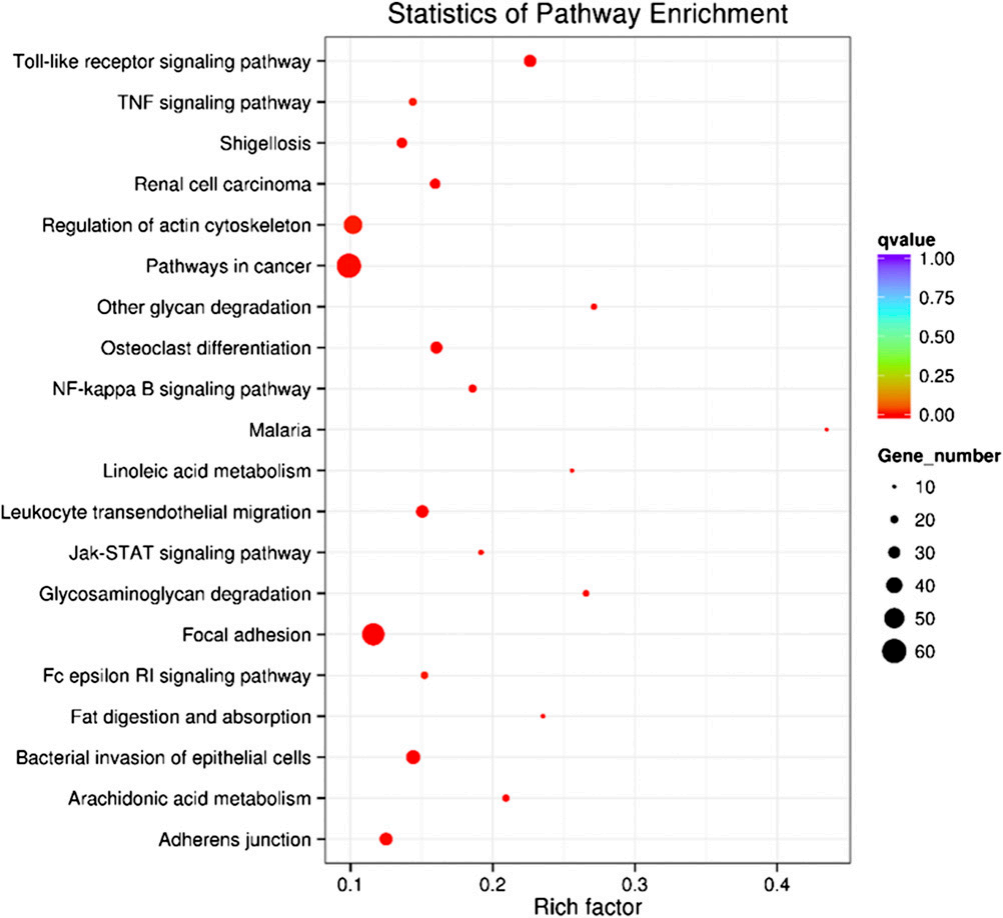
KEGG enrichment scatterplots of the upregulated genes after metamorphosis. The vertical axis represents the pathway name; the horizontal axis represents the corresponding enrichment factor. The q-value is corresponds to a points’ color: the redder the color, the smaller the q-value. Finally, a points’ size indicates the number of DEG in each pathway.

Gene products usually interact with each other *in vivo* to play roles in certain biological functions. KEGG pathway enrichment was carried out to identify DEGs involved in the main biochemical pathways and signal transduction pathways. Scatterplots of DEGs showed the analysis results of KEGG pathway enrichment. We display the top 20 significantly enriched pathways of the J4 *vs.* Y5 comparison in Figure 6. KEGG enrichment analyses of other developmental comparison groups are shown in Figure S3. Some immune-related pathways, including “Toll-like receptor (TLR) signaling,” “bacterial invasion of epithelial cells,” and “Leukocyte transendothelial migration,” were highly enriched as upregulated pathways after metamorphosis.

### qPCR validation

**Figure 7:**
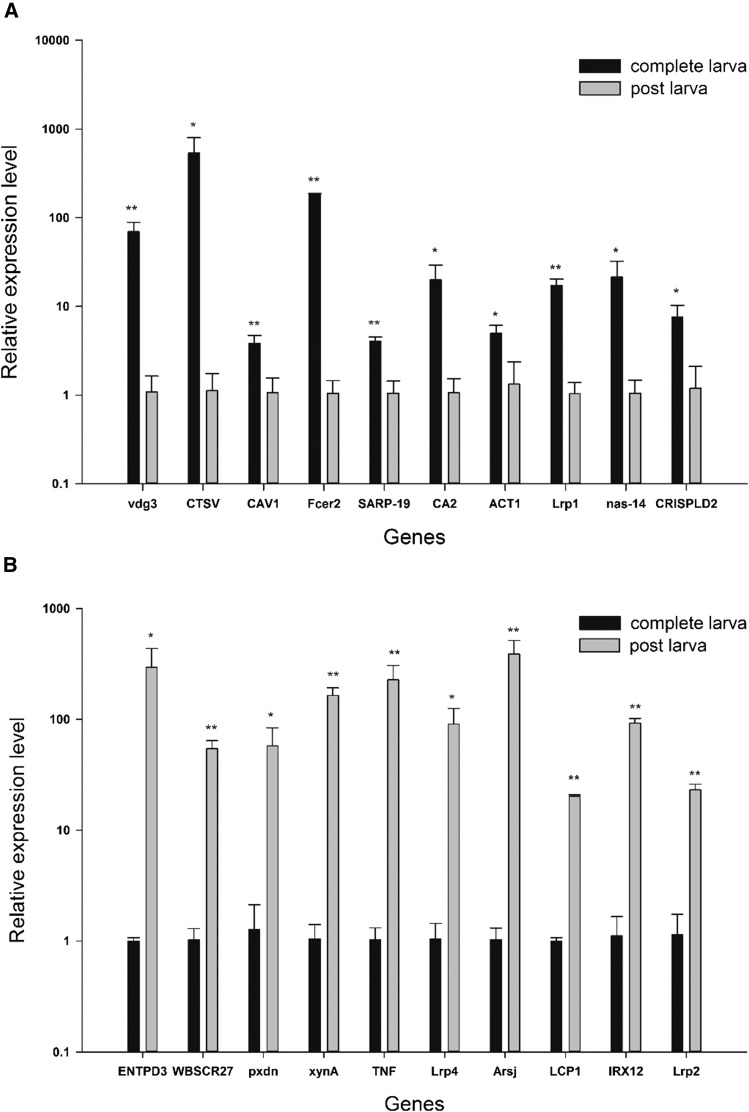
Relative expression levels for 20 differentially expressed genes in competent larva and postlarval stages, as determined by qRT-PCR. (A) Ten genes that were downregulated after metamorphosis. vdg3, vdg3; CTSV, cathepsin L2; CAV, caveolin-1; Fcer2, low affinity immunoglobulin epsilon Fc receptor; SARP-19, SARP-19; CA2, carbonic anhydrase 2; ACT1, actin; Lrp1, prolow-density lipoprotein receptor-related protein 1; nas-14, zinc metalloproteinase nas-14; and CRISPLD2, cysteine-rich secretory protein LCCL domain-containing 2. (B) Ten genes that were upregulated after metamorphosis. ENTPD3, ectonucleoside triphosphate diphosphohydrolase 3; WBSCR27, Williams-Beuren syndrome chromosomal region 27 protein; pxdn, peroxidasin; xynA, beta-1,4-xylanase; TNF, tumor necrosis factor; Lrp4, low-density lipoprotein receptor-related protein 4; Arsj, arylsulfatase J; LCP1, digestive cysteine proteinase 1; IRX12, Laccase-4; Lrp2, low-density lipoprotein receptor-related protein 2. Values are the mean ± SD (*n* = 3). * *P* < 0.05, ** *P* < 0.01.

To validate the RNA-seq results, 20 DEGs between J4 and Y5 were selected and analyzed by qRT-PCR, including the 10 most upregulated DEGs, and the 10 most downregulated DEGs. Results in Figure 7 show that the DGE tag profiling had a good consistency with the qRT-PCR results, although the fold changes in expression differed substantially in some cases, likely caused by differences in the sensitivity of estimating gene expression (Zhao *et al.* 2014).

## Discussion

A knowledge of the developmental biology of *R. venosa* is essential for scientific research, invasion control, and aquaculture. To promote work in this field, we constructed a digital gene expression profile on the different larval stages of *R. venosa*, and thus could globally identify genes participating in important early developmental bioprocesses.

There are visible morphological and body size differences between these five stages. We observed that planktonic larvae (C1, D2, F3, and J4) could swim freely and feed on microalgae via their ciliary structure velum. The competent larvae (J4) of *R. venosa* quickly undergo metamorphosis when they find suitable habitats, with specific cues being most mollusk-like. Thus, we speculated that the nervous system participates in reacting with chemical cues, and activating metamorphosis. This pelagic-to-benthic transition took only 3 d, but involved complicated morphological and physiological changes. First, the velum for swimming and ingestion was degenerated and reabsorpbed during metamorphic transition. Second, secondary shell, foot, and extensile tentacles with eyes develop further in postlarvae. Third, phytophagous pelagic larvae develop into carnivorous juveniles. Based on these morphological and physiological changes, DEGs related to growth, nervous system, digestive system, immune system, and apoptosis were considered to play important roles in *R. venosa* development, especially in metamorphosis. Thus, a preliminary differentiated analysis of these interesting DEGs was performed, revealing that several genes had quite different expression patterns at different stages, as displayed in Table 3. Some useful information can be obtained as follows.

**Table 3:** Genes selected for real-time PCR validation and the fold change in the postlarval stage (Y5) compared with the four-spiral whorls stage (competent larvae J4) for both RNA-seq and RT-PCR analysis

			log2.fold_change (Y5 *vs.* J4)		
Gene ID	Gene Name	Exp	RNA-Seq	RT-PCR	Same Trend
c95470_g1	vdg3	↓	−9.55**	−6.00**	Y
c131243_g1	CTSV	↓	−8.40**	−8.90*	Y
c139260_g1	CAV1	↓	−7.39**	−1.84**	Y
c129336_g1	Fcer2	↓	−6.30**	−7.46**	Y
c125484_g1	SARP-19	↓	−5.75**	−2.01**	Y
c128565_g1	CA2	↓	−5.63**	−4.23*	Y
c145993_g2	ACT1	↓	−5.02**	−1.91*	Y
c127784_g1	Lrp1	↓	−4.71**	−4.06**	Y
c145412_g1	nas-14	↓	−4.59**	−4.35*	Y
c119967_g1	CRISPLD2	↓	−4.29**	−2.67*	Y
c150152_g1	ENTPD3	↑	4.56**	8.20*	Y
c113545_g2	WBSCR27	↑	4.50**	5.73**	Y
c94349_g1	pxdn	↑	4.49**	5.50*	Y
c156029_g2	xynA	↑	4.43**	7.30**	Y
c117375_g2	TNF	↑	4.38**	7.79**	Y
c147483_g1	Lrp4	↑	4.34**	6.44*	Y
c149073_g1	Arsj	↑	4.31**	8.56**	Y
c143535_g1	LCP1	↑	3.89**	4.33**	Y
c135964_g1	IRX12	↑	3.85**	6.37**	Y
c118662_g1	Lrp2	↑	3.81**	4.34**	Y

Genes with the same trend (up or downregulation) as shown by both RNA-seq and RT-PCR analysis are indicated by “Y” (yes). * *P* < 0.05, ** *P* < 0.01 (significant difference between J4 and Y5 stages). Exp, expression trend; “↑”upregulated expression; “↓” downregulated expression.

### Growth

The abundance levels of Actin (c145993_g2) increased by nearly 150-fold when the larvae transformed from precompetent larvae into competent larva. Expression dropped by 36-fold from the competent larval stage to the postlarval stage. The actin protein forms microfilaments, and functions in retraction and resorption of the sand dollar larval arms during metamorphosis (Burke 1985). The competent stage-specific expression of these mRNAs might be important for the initiation of metamorphosis. The expression of fibropellin-1 was detected at a low level in precompetent larvae, but increased significantly in competent larvae, and was further upregulated after metamorphosis. Fibropellin-1 was first identified in the sea urchin, and is also called epidermal growth factor-related protein 1. It is thought to regulate species-specific signal transduction pathways in the early development of sea urchin, and loss of fibropellin-1 results in development defects (Kamei *et al.* 2000; Yang *et al.* 1989).

### Nervous system

The expression level of 5-hydroxytryptamine receptor, neuronal acetylcholine receptor, and nitric oxide synthase were found declined after metamorphosis. Serotonin and its receptors are involved in neuronal functions in mollusks, including the circadian rhythm of *Aplysia californica* (Levenson *et al.* 1999), locomotion of *Lymnaea stagnalis* (Filla *et al.* 2004), feeding of *L. stagnalis* (Kawai *et al.* 2011), and development of *Haliotis rubra* (Panasophonkul *et al.* 2009). Pharmacological and ecological experiments suggested that the 5-hydroxytryptamine receptor mediates settlement and metamorphosis of many mollusks, such as *Ilyanassa obsolete* (Leise *et al.* 2001) and *Helisoma trivolvis* (Glebov *et al.* 2014). The expression level of the 5-HT receptor was downregulated in *H. trivolvis* during the transition from the premetamorphic stage to the metamorphic stage (Glebov *et al.* 2014). Nitric oxide synthase, and its synthetic product nitric oxide (NO), were purported to play a part in metamorphosis of mollusks (Laudet *et al.* 2013). It was also observed in the mud snail *I. obsolete* that expression of NOS was downregulated after the initiation of metamorphosis (Froggett and Leise 1999). The suppression of NOS activity by NOS-inhibiting pharmacological agents has been reported to induce the initiation of metamorphosis, leading to the hypothesis that NOS plays a negative regulatory role (Froggett and Leise 1999). Against that, NOS and NO were considered to be positive regulators for the initiation of metamorphosis in some gastropods, such as *H. asinina* (Ueda and Degnan 2014).

### Digestive system

Consistently, we also identified genes that are involved in the histogenesis and digestion processes of the digestive system. The expression level of developmentally regulated *vdg3* (c105120_g1) increased rapidly during the development of *R. venosa*, and was significantly upregulated after metamorphosis. This expression pattern was similar to its counterparts in *H. diversicolor* and *H. asinina*, which are specifically expressed in the digestive tract and glands, and increased by over 10-fold when larvae undergo the transition from pelagic to benthic lifestyle, suggesting that the gene is involved in gut morphogenesis and digestion (He *et al.* 2014; Jackson *et al.* 2005). Interestingly, expression pattern of *vdg3* (c95470_g1) is the complete opposite of the developmentally regulated *vdg3*. We speculated that these two genes have antagonistic effects on the regulation of development and metamorphosis in *R. venosa*. The expression pattern of *vdg3* was also contrary to its counterpart in *H. diversicolor* (He *et al.* 2014); therefore, this DGE profile is an interesting phenomenon that requires further study.

The carnivorous digestive enzymes trypsin (c128291_g1) and carboxypeptidase B (c147105_g1) had an extremely low expression level in the precompetent larval stage, a slight increase in the competent larva stage, and reached a very high level in the postlarva stage. Trypsin is a type of serine protease. In the digestive system of many vertebrates, trypsin participates in the hydrolysis of protein. Carboxypeptidase B is known as a protease enzyme that can cleave a peptide bond at the C-terminal end of proteins or peptides. A high level of these enzymes in the post larval stage indicates that the digestion process is active. Instead, the gene expression level of some phytophagous digestive enzymes such as exoglucanase (c150903_g1) and endoglucanase (c154739_g1) remained at a high level in precompetent larvae, but obviously dropped when larvae went through metamorphic transition. The pelagic larvae of *R. venosa* (precompetent and competent) feed on microalgae, while the juveniles prey on bivalve mollusks. We hypothesized that the upregulation of these proteases is responsible for the change in diet after metamorphosis, and that competent larvae is an important interim period during which the expression of different kinds of digestive enzymes is regulated.

Conotoxin (c124801_g1) was found highly expressed in the pelagic larval stage, and quickly declined to almost zero. Conotoxin is one of a group of neurotoxic peptides isolated from the venom of mollusks of the genus *Conus*, which enables the marine cone snail to carry out its unique predatory lifestyle (Olivera *et al.* 1991). Conotoxins have promising applications as drugs (Terlau and Olivera 2004). The abundant expression of conotoxin in this study indicated that the pelagic larvae of *R. venosa* and *Conus* were homologous, but conotoxin in *R. venosa* might degenerate after metamorphosis. The conotoxin of *R. venosa* warrants further research, because there is no report of conotoxins in this species.

### Immune system

Many immune-related genes, such as toll 2 (c149483_g1), defensin (c137778_g1), and tumor necrosis factor (c115222_g1), were also abundantly expressed during post larval stage. We speculated that the benthic larvae are more exposed to pathogens when acquiring food in their environment, therefore a stronger immune response is stimulated (Huang *et al.* 2012). Furthermore, “immune system process” and “immune response” items were significantly upregulated in the GOBP group of GO enrichment, and “alpha-l-fucosidase activity” and “fucosidase activity” were significantly upregulated in the GOMF group. Fucosidase and peptidase were reported to function in the digestive system, as well as playing an important role in host/parasite interactions in *Biomphalaria glabrata* (Perrella *et al.* 2015). This may explain the changes in the immune response after the metamorphic conversion from microalgae diet to the bivalve diet of *R. venosa*. We estimated that the immune response changes before and after metamorphosis were significantly different because the benthic post larva feeds on bivalves, which are more susceptible to parasites than planktonic competent larva feeding on microalgae. Besides, the KEGG enrichment also showed that the TLR signaling pathway was the most significantly enriched (q-value = 7.39e–07). The TLR is a member of pattern-recognition receptors (PRRs), and TLR, together with its signaling pathway, play a critical role in recognizing various pathogen-associated molecular patterns, and represent the front line of defense against invading pathogens (Wang *et al.* 2011). Interestingly, other immune-related pathways, including “bacterial invasion of epithelial cells,” and “leukocyte transendothelial migration,” were highly enriched in the postlarval stage, indicating that the changes in the immune system were an important first-line defense in the response against the transformation of habitat and predatory feeding habit after metamorphosis. Metamorphosis of *M. galloprovincialis* was suggested as a pivotal stage when postlarvae respond to environmental signals and upregulated the expression level of immune system-related genes (Balseiro *et al.* 2013). The expression of most genes involved in immune response in the postlarval stage was significantly upregulated compared to oocytes, trochophores and veligers, suggesting active immune activity in the mussel postlarvae stage. This pattern of gene expression is always followed by the expression of some immune or nonimmune related proteins, based on previous ontogeny studies in mollusks (Ge *et al.* 2011; Tirapé *et al.* 2007). In the ascidian *Boltenia villosa*, immune-related genes were also upregulated during metamorphosis, highlighting the specific role of innate immunity during metamorphosis (Davidson and Swalla 2002). This observed upregulation during metamorphosis might simply reflect immune system maturation, but might also involve the resorption and reorganization of larval tissues, or involve the larvae’s ability to detect and respond to bacterial cues for settlement and metamorphosis (Balseiro *et al.* 2013).

### Apoptosis

As a distinguishing feature of metamorphosis in *R. venosa*, the velum of competent larvae will be lost by programmed cell death. Therefore, we investigated the expression pattern of some apoptosis genes, as shown in Table 2. The apoptosis inducer (c132048_g1) and executors (c135194_g1, c147256_g2) were found to be downregulated when switched from competent to postlarvae. And the apoptosis 2 inhibitor (c151900_g1) was upregulated after metamorphosis. This conformed well to the morphological transformation during metamorphosis in *R. venosa*. Many studies have shown that caspases are significantly expressed in larvae such as those of *C. angulate* (Yang *et al.* 2012), *M. galloprovincialis* (Romero *et al.* 2011), and *Ilyanassa obsolete* (Gifondorwa and Leise 2006), prior to metamorphosis, suggesting caspases play an important role in larval settlement and metamorphosis of mollusks.

### Others

The expression of calmodulin (c116117_g1) was at a low level in competent larval stages, but increased significantly after metamorphosis. It is considered a multifunctional metabolic regulator that can interact with diverse signaling proteins, including phosphatases, kinases, and membrane receptors. Calmodulin and its associated binding proteins play important roles in many biological processes, such as cell growth, neuronal development, and bacterial pathogenesis (O’Day 2003). This protein showed high expression in 12 hr postmetamorphic juveniles of *Hydroides elegans*, and remained at a high level in adults. *In situ* hybridization revealed that, in putative growth zones, the calmodulin gene was continuously expressed, indicating its function in tissue differentiation and development (Chen *et al.* 2012). It also was reported to participate in the biomineralization process in *Pinctada fucata* (Fang *et al.* 2008).

c112229_g1 is highly similar to SARP 19, which was discovered in the marine snail *Littorina littorea* as a protein function in the anoxia response of the snail (Larade and Storey 2004). In contrast to this study, SARP 19 increased during the development of *H. diversicolor*, and its expression showed a sharp increase after settlement (He *et al.* 2014).

In addition, c125727_g1 and c141012_g1, which were highly similar to Sulfotransferase 1C2 and Sulfotransferase 1A2, respectively, had a stage-specific high expression at competent larval stages. Using 3′-phosphoadenosine-5′-phosphosulfate as the most common sulfo group donor, the sulfotransferase 1 family functions in detoxification of xenobiotics, and modulation of endogenous hormones, bile acids, and neurotransmitter activities, such as estrogens, iodothyronines, and catecholamines (Blanchard *et al.* 2004; Gamage *et al.* 2006). These proteins are suggested to participate in development and metamorphosis in the bullfrog, *Rana catesbeiana* (Rahman and Yamauchi 2010); however, there is little information on their function in gastropods.

To estimate expression patterns of DEGs under different developmental conditions, 1794 genes selected according to the criteria detailed above were clustered hierarchically. Through clustering genes with similar expression patterns, groups of genes with similar patterns but unknown functions could be recognized, because transcripts with similar expression patterns might have similar roles in the same cellular pathways or metabolic processes. The information from the horizontal axis of the gene clustering dendrogram indicated that the developmental stages were aggregated into two distinct clusters. The one-, two- and three-spiral whorls stages formed one cluster, and the four-spiral whorls stage (competent larva) and postlarval stage formed another. This clustering pattern is in accordance with *Haliotis diversicolor* (Huang *et al.* 2012) and *H. asinina* (Williams *et al.* 2009). This also explained the anticipatory development phenomenon that some postlarval structures and organs were actually formed before metamorphosis (Degnan and Morse 1995), and also indicated that the later competent larvae require more specific transcripts preparing for settlement and metamorphosis than precompetent larvae.

### Conclusions

Research on the larval development and metamorphosis mechanisms of *R. venosa* remains scarce due to limited genomic resources. Thus, in a previous work, we constructed a comprehensive transcriptome database by *de novo* sequencing of a mixed RNA pool from pelagic larvae and juveniles (Song *et al.* 2016). This work identified 212,049 unigenes, of which 70,877 were functionally annotated. However, it did not reveal the dynamic changes and expression patterns of different genes.

Using the previous transcriptome database as reference, in this study we generated a transcriptomic DGE sequencing strategy with a careful design and strict quality controls, and constructed a global gene expression profile of *R. venosa* during early development, from planktonic larval stages to the post larval stage. Our global DGE profiles provided the useful temporal dynamics of the expressions of thousands of genes. It is the first digital gene profile to cover the early developmental stages of *R. venosa*. By cluster analysis, we also identified a series of candidate genes that might play an important role in early development of *R. venosa*, especially in metamorphosis regulation. Interestingly, we observed that some DEGs associated with growth, nervous system, digestive system, immune system, and apoptosis participate in important developmental processes. These data provide significant clues for a better understanding of the regulatory mechanisms of development in *R. venosa*, and identified some key genes worthy of further research.

## Supplementary Material

Supplemental Material
