# Supplementary material for: Transcriptomic Analysis of Differentially Expressed Genes During Larval Development of Rapana venosa by Digital Gene Expression Profiling
Source: G3 (Bethesda). 2016 May 18;6(7):2181–93. doi: 10.1534/g3.116.029314 (PMC4938671; doi:10.1534/g3.116.029314)
Supplement: Supplemental Material [file supp_6_7_2181__index.html]

Transcriptomic Analysis of Differentially Expressed Genes During Larval Development of Rapana venosa by Digital Gene Expression Profiling — Supplemental Material 

# Transcriptomic Analysis of Differentially Expressed Genes During Larval Development of *Rapana venosa* by Digital Gene Expression Profiling

## Supplemental Material for Song *et al.*, 2016

**Files in this Data Supplement:**

- Figure S1 - Volcano plots show the up/down-regulation of DEGs in each comparison. (.zip, 734 KB)
- Figure S2 - Gene ontology (GO) enrichment analysis of developmental comparison groups. (.zip, 179 KB)
- Figure S3 - Kyoto encyclopedia of gene and genomes (KEGG) enrichment analysis of other developmental comparison groups. (.zip, 182 KB)
- Table S1 - Gene list for the three clusters shown in Figure 6. (.xlsx, 30 KB)
- Table S2 - Primers for qPCR. (.xlsx, 12 KB)
- Table S3 - Differentially expressed genes among five developmental stages of *R.venosa*. (.xlsx, 31 MB)
