# Supplementary figures and images for: Transcriptomic Analysis of Differentially Expressed Genes During Larval Development of Rapana venosa by Digital Gene Expression Profiling
Source: G3 (Bethesda). 2016 May 18;6(7):2181–93. doi: 10.1534/g3.116.029314 (PMC4938671; doi:10.1534/g3.116.029314)

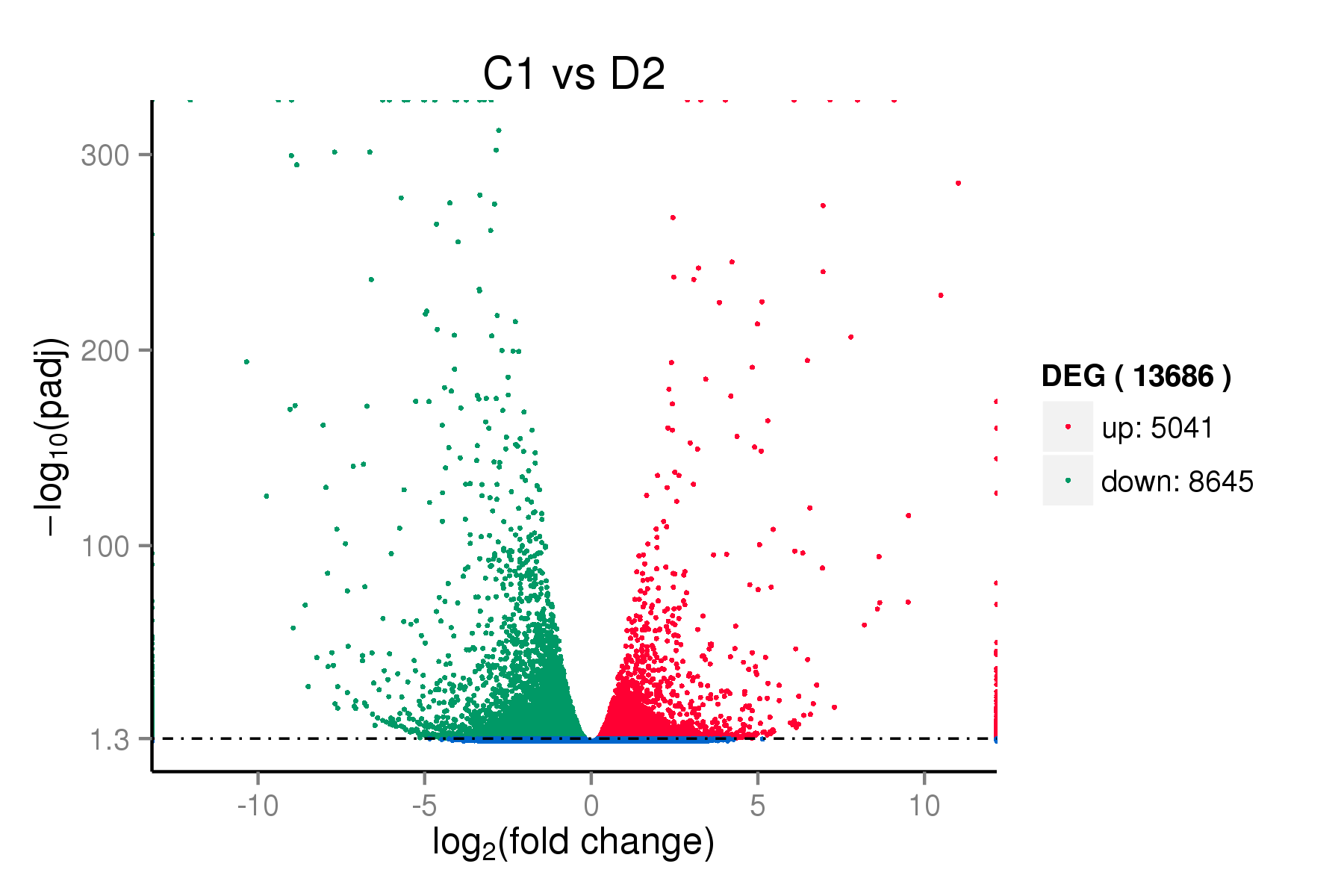

Supplement: Supplemental Material [file supp_g3.116.029314_FigureS1.zip › Figure S1. Volcano plots show the up and down regulation of DEGs in each comparison/C1vsD2.Volcanoplot.png]

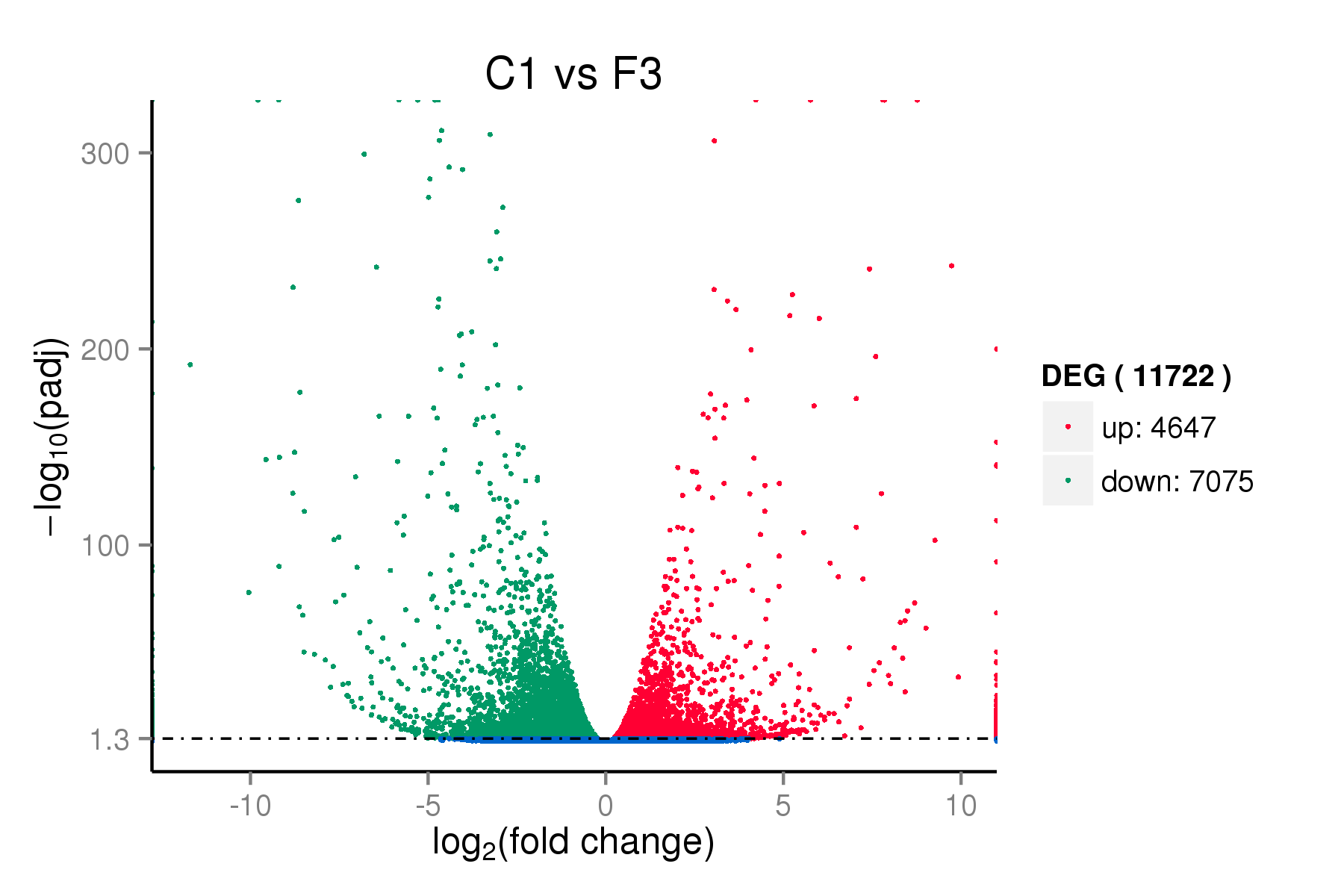

Supplement: Supplemental Material [file supp_g3.116.029314_FigureS1.zip › Figure S1. Volcano plots show the up and down regulation of DEGs in each comparison/C1vsF3.Volcanoplot.png]

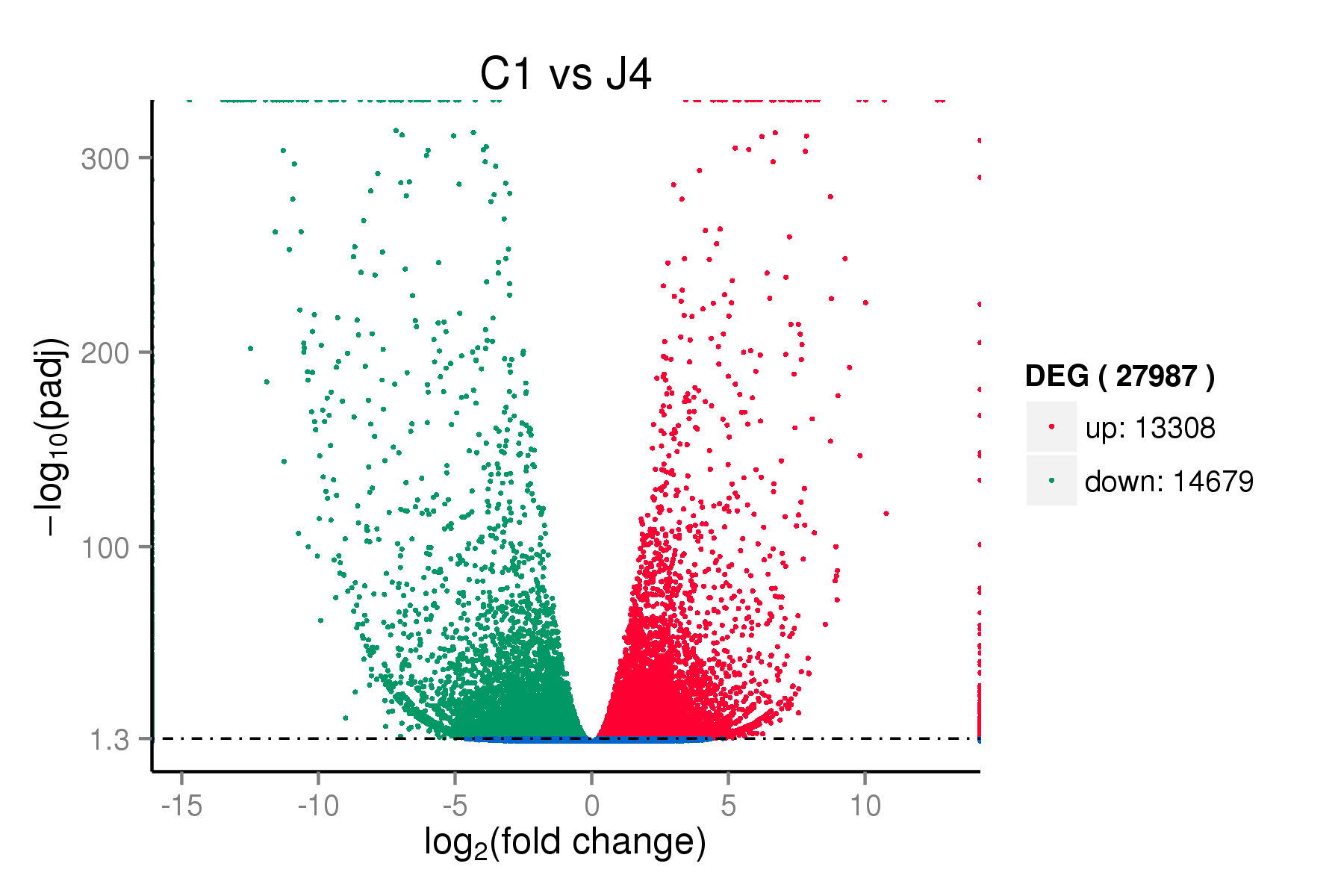

Supplement: Supplemental Material [file supp_g3.116.029314_FigureS1.zip › Figure S1. Volcano plots show the up and down regulation of DEGs in each comparison/C1vsJ4.Volcanoplot.png]

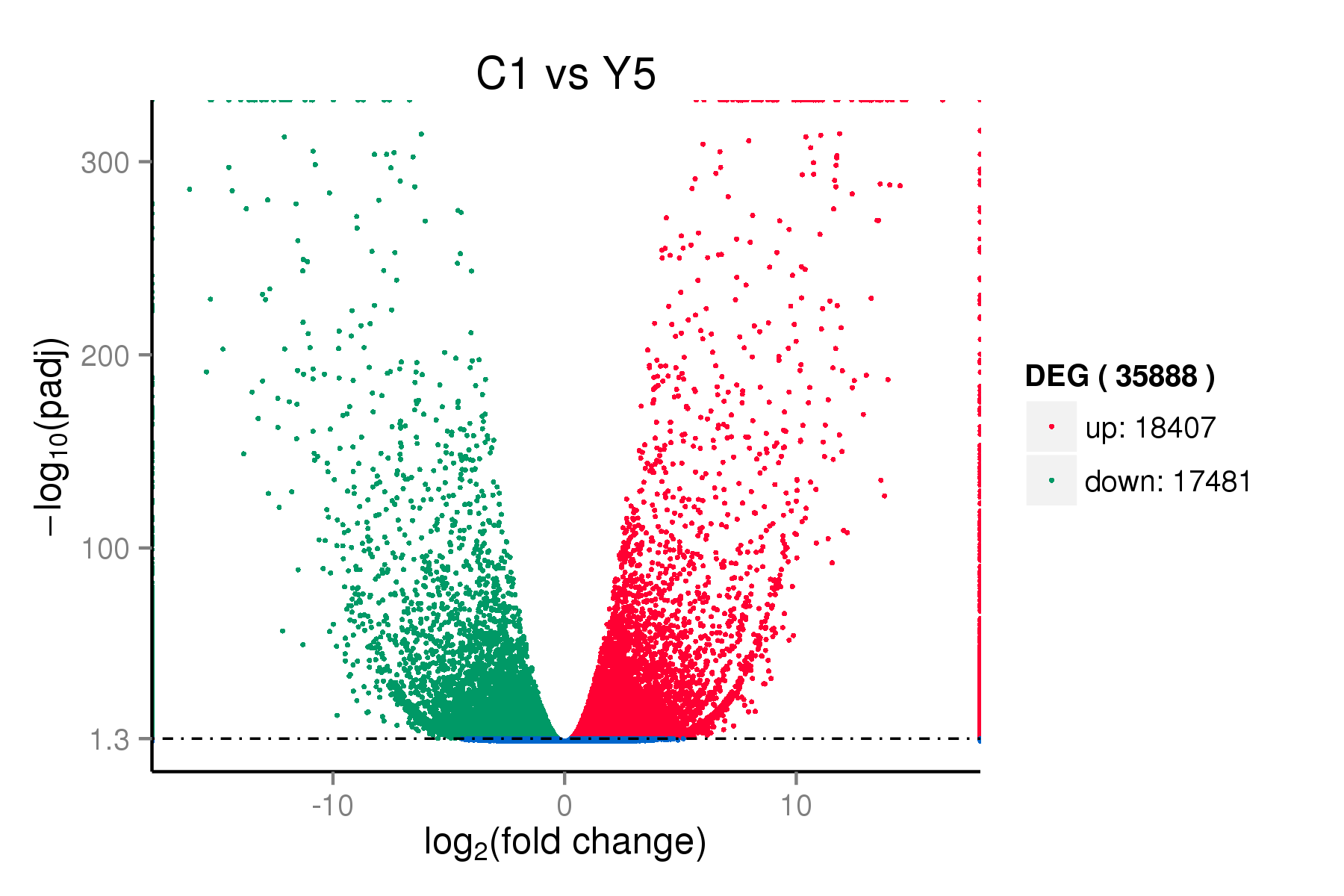

Supplement: Supplemental Material [file supp_g3.116.029314_FigureS1.zip › Figure S1. Volcano plots show the up and down regulation of DEGs in each comparison/C1vsY5.Volcanoplot.png]

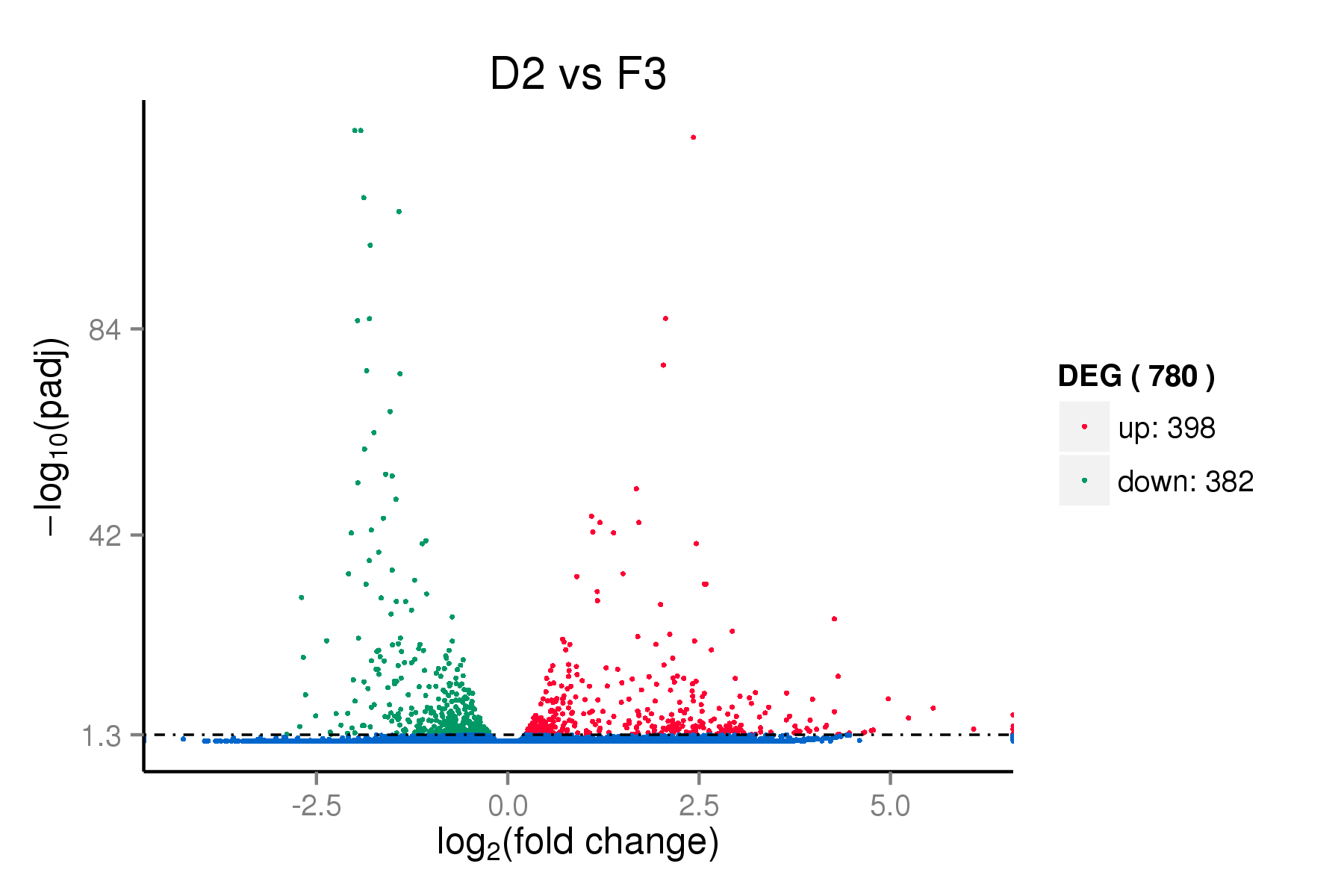

Supplement: Supplemental Material [file supp_g3.116.029314_FigureS1.zip › Figure S1. Volcano plots show the up and down regulation of DEGs in each comparison/D2vsF3.Volcanoplot.png]

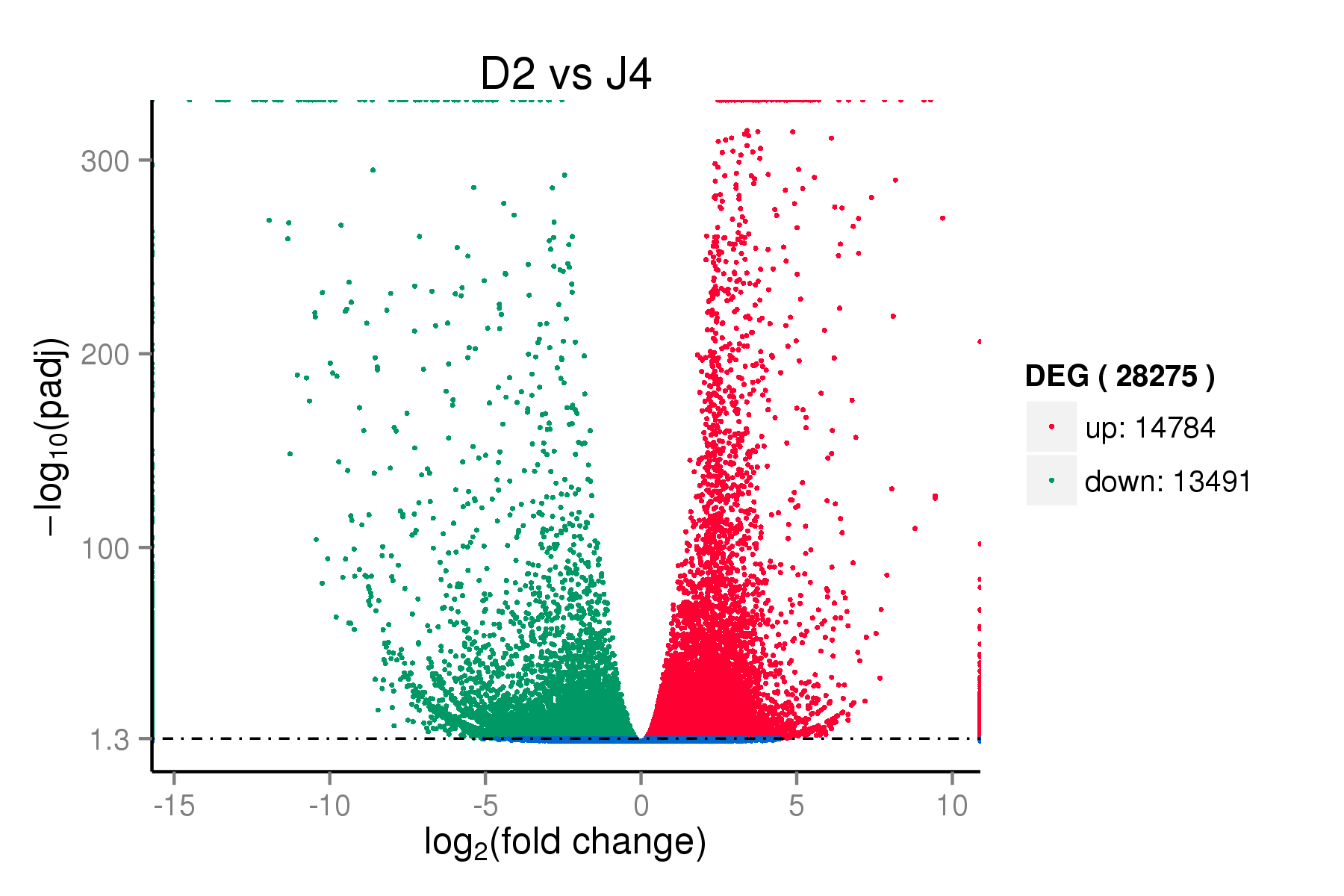

Supplement: Supplemental Material [file supp_g3.116.029314_FigureS1.zip › Figure S1. Volcano plots show the up and down regulation of DEGs in each comparison/D2vsJ4.Volcanoplot.png]

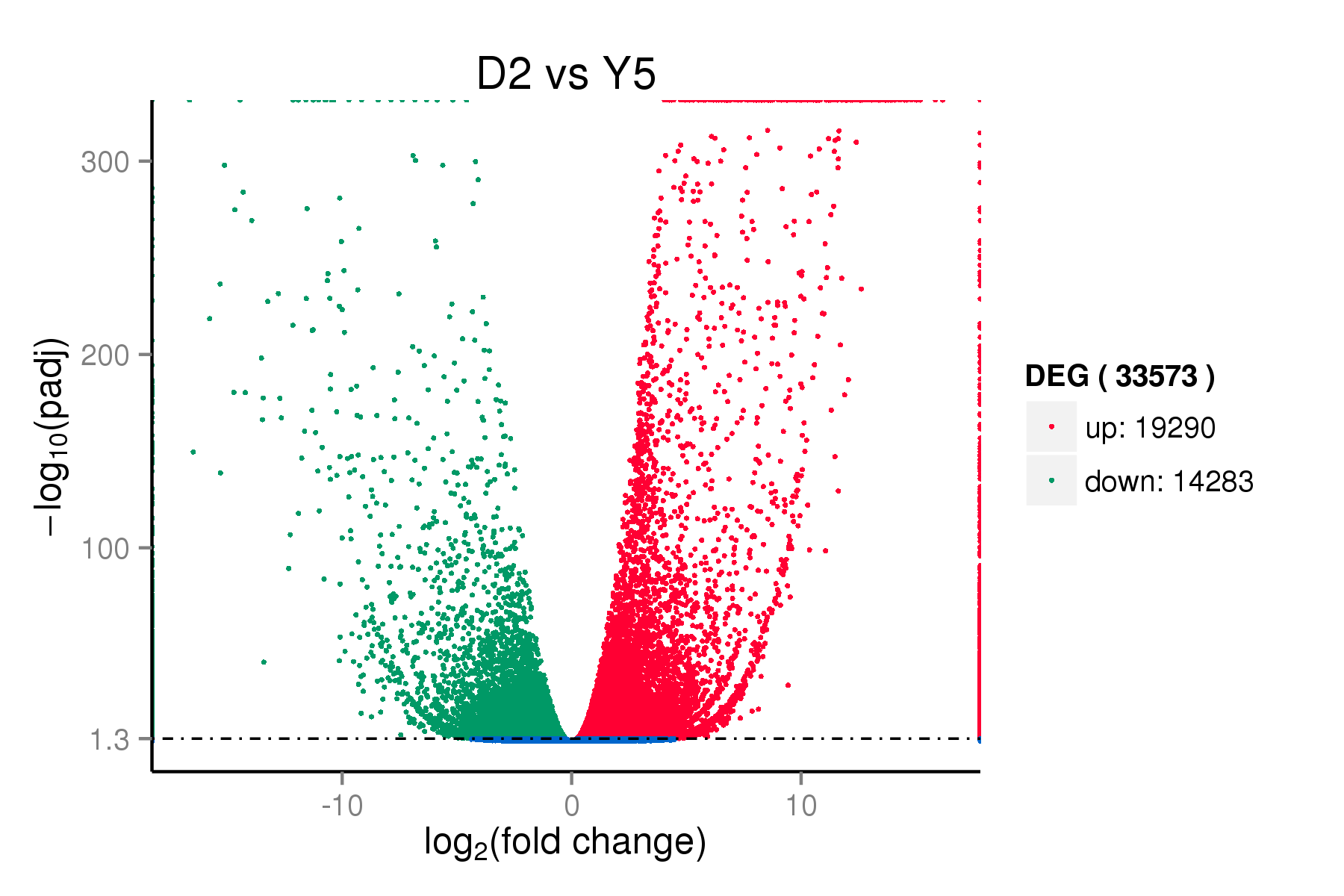

Supplement: Supplemental Material [file supp_g3.116.029314_FigureS1.zip › Figure S1. Volcano plots show the up and down regulation of DEGs in each comparison/D2vsY5.Volcanoplot.png]

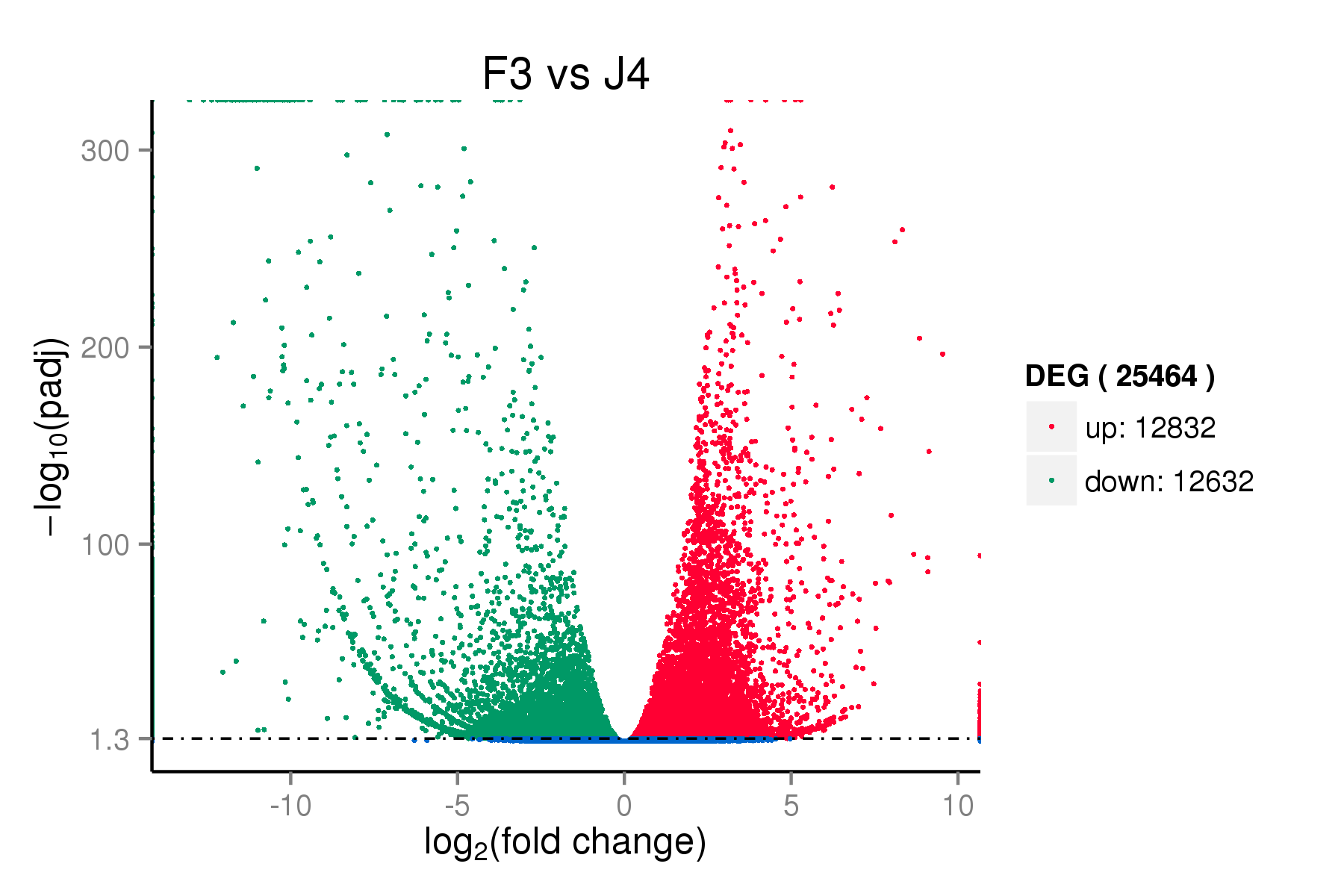

Supplement: Supplemental Material [file supp_g3.116.029314_FigureS1.zip › Figure S1. Volcano plots show the up and down regulation of DEGs in each comparison/F3vsJ4.Volcanoplot.png]

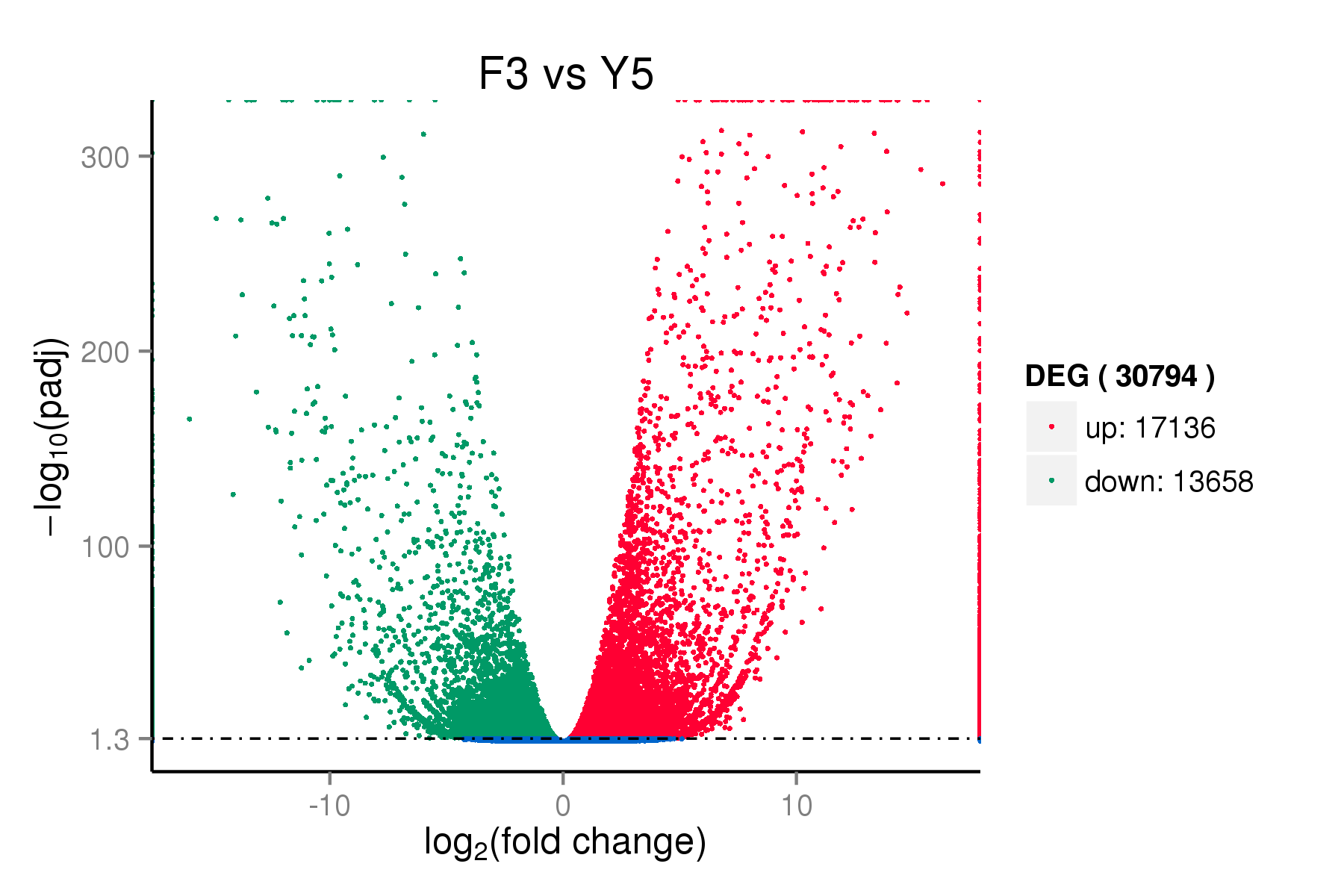

Supplement: Supplemental Material [file supp_g3.116.029314_FigureS1.zip › Figure S1. Volcano plots show the up and down regulation of DEGs in each comparison/F3vsY5.Volcanoplot.png]

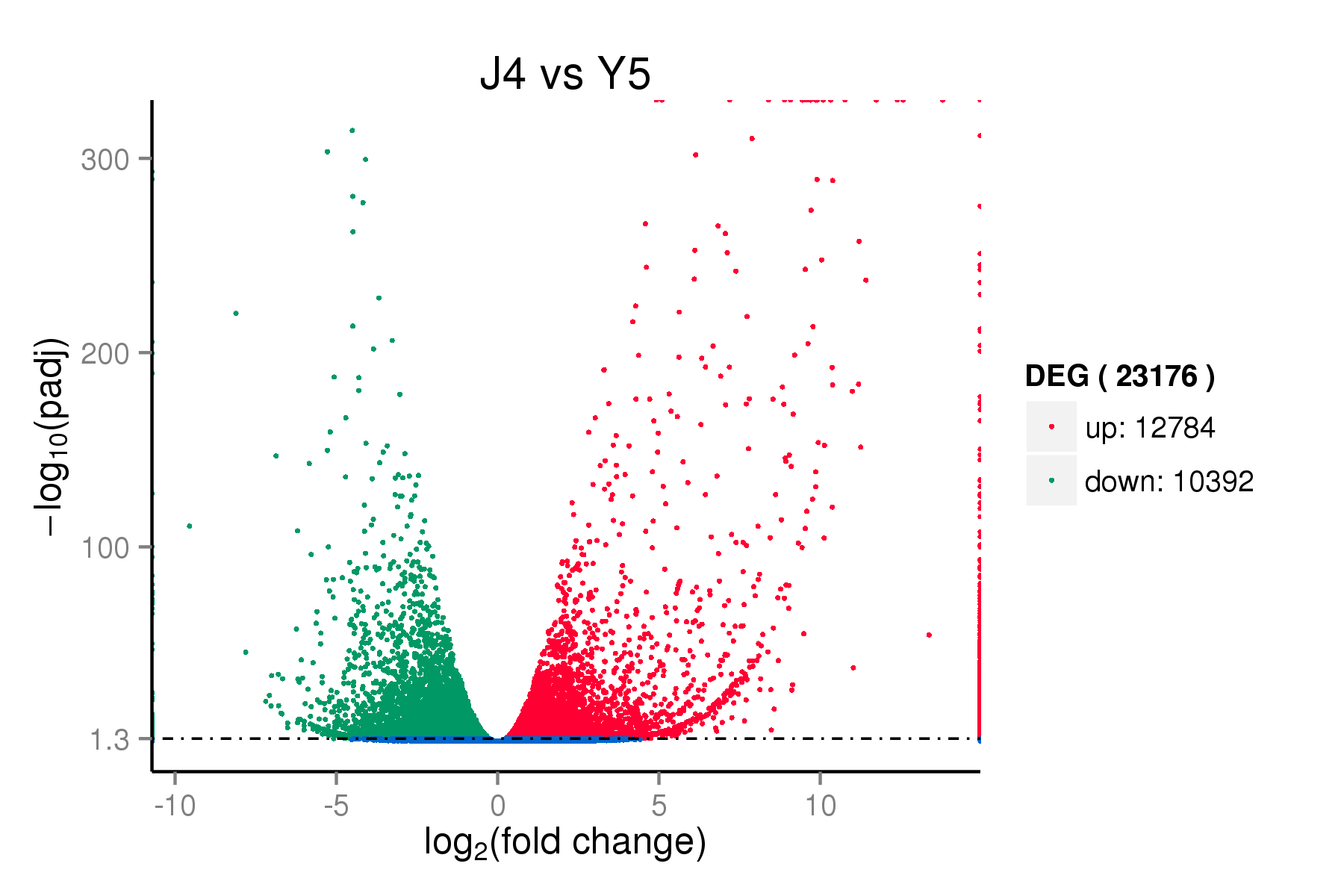

Supplement: Supplemental Material [file supp_g3.116.029314_FigureS1.zip › Figure S1. Volcano plots show the up and down regulation of DEGs in each comparison/J4vsY5.Volcanoplot.png]

Enriched GO Terms  
(C1vsD2)

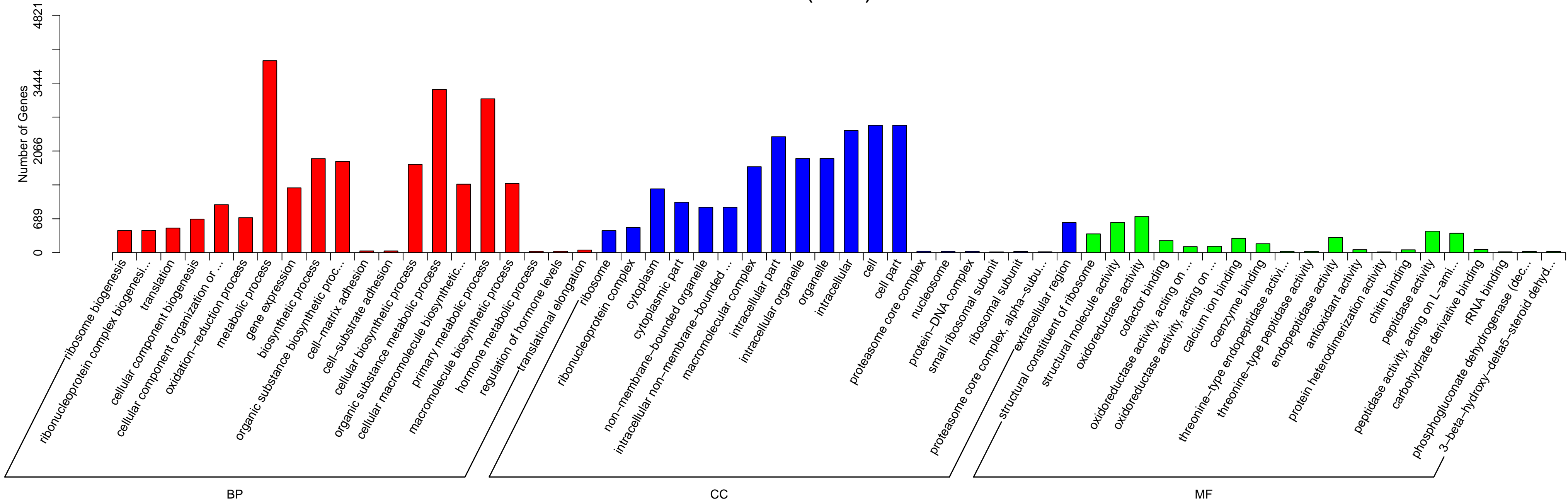

Supplement: Supplemental Material [file supp_g3.116.029314_FigureS2.zip › Figure S1. GO enrichment analysis of developmental comparison groups/C1vsD2.DEG_Enriched_GO_classification.pdf]

Enriched GO Terms  
(C1vsD2\_down)

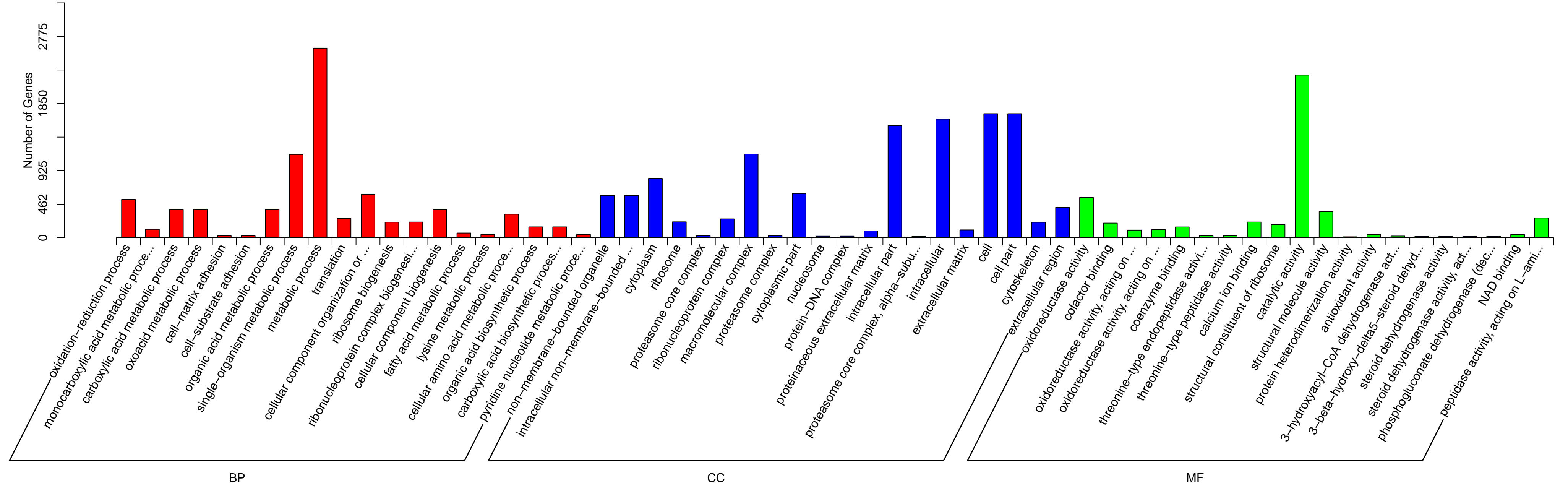

Supplement: Supplemental Material [file supp_g3.116.029314_FigureS2.zip › Figure S1. GO enrichment analysis of developmental comparison groups/C1vsD2_down.DEG_Enriched_GO_classification.pdf]

Enriched GO Terms  
(C1vsD2\_up)

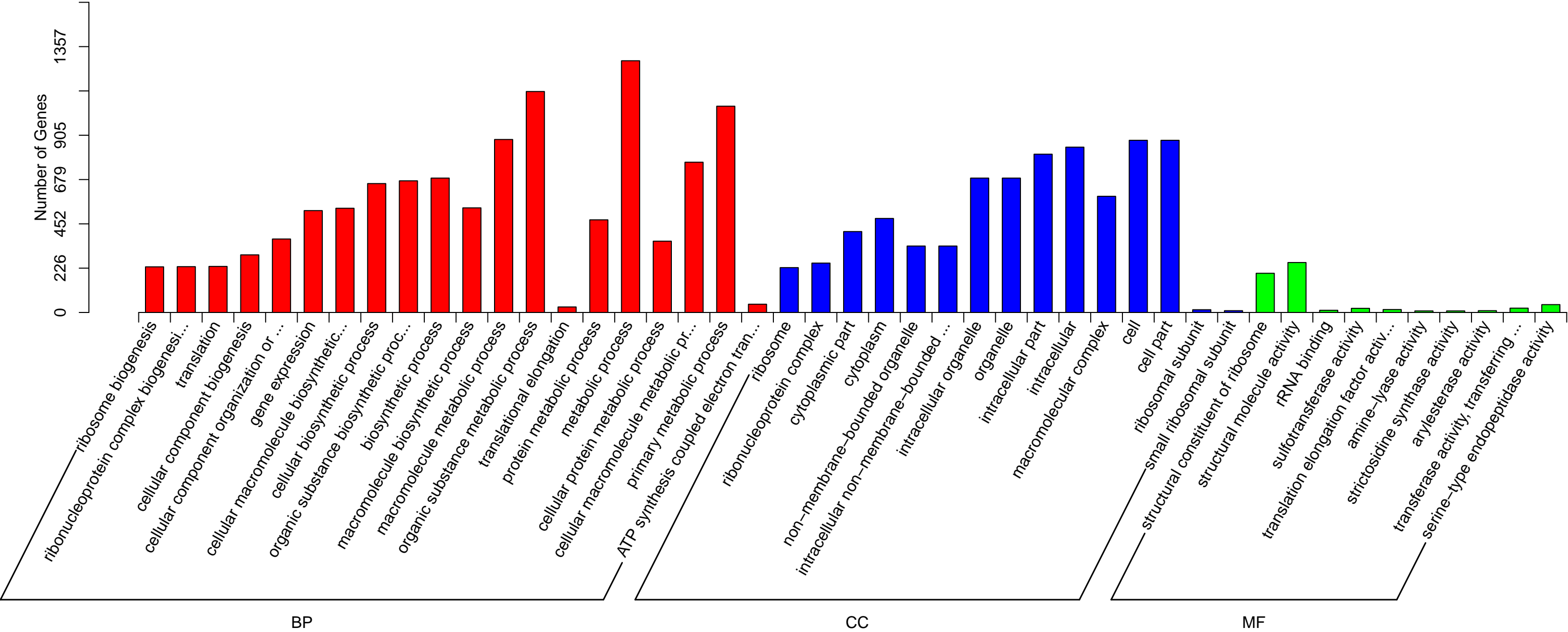

Supplement: Supplemental Material [file supp_g3.116.029314_FigureS2.zip › Figure S1. GO enrichment analysis of developmental comparison groups/C1vsD2_up.DEG_Enriched_GO_classification.pdf]

# Enriched GO Terms (C1vsF3)

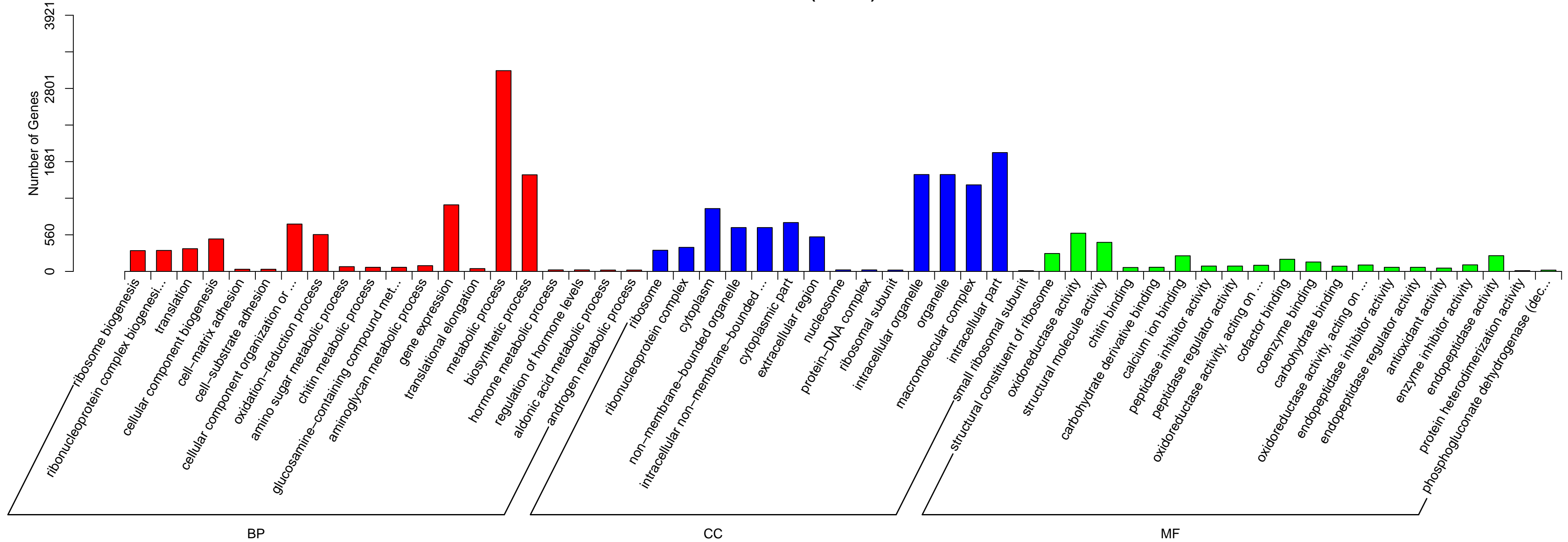

Supplement: Supplemental Material [file supp_g3.116.029314_FigureS2.zip › Figure S1. GO enrichment analysis of developmental comparison groups/C1vsF3.DEG_Enriched_GO_classification.pdf]

Enriched GO Terms  
(C1vsF3\_down)

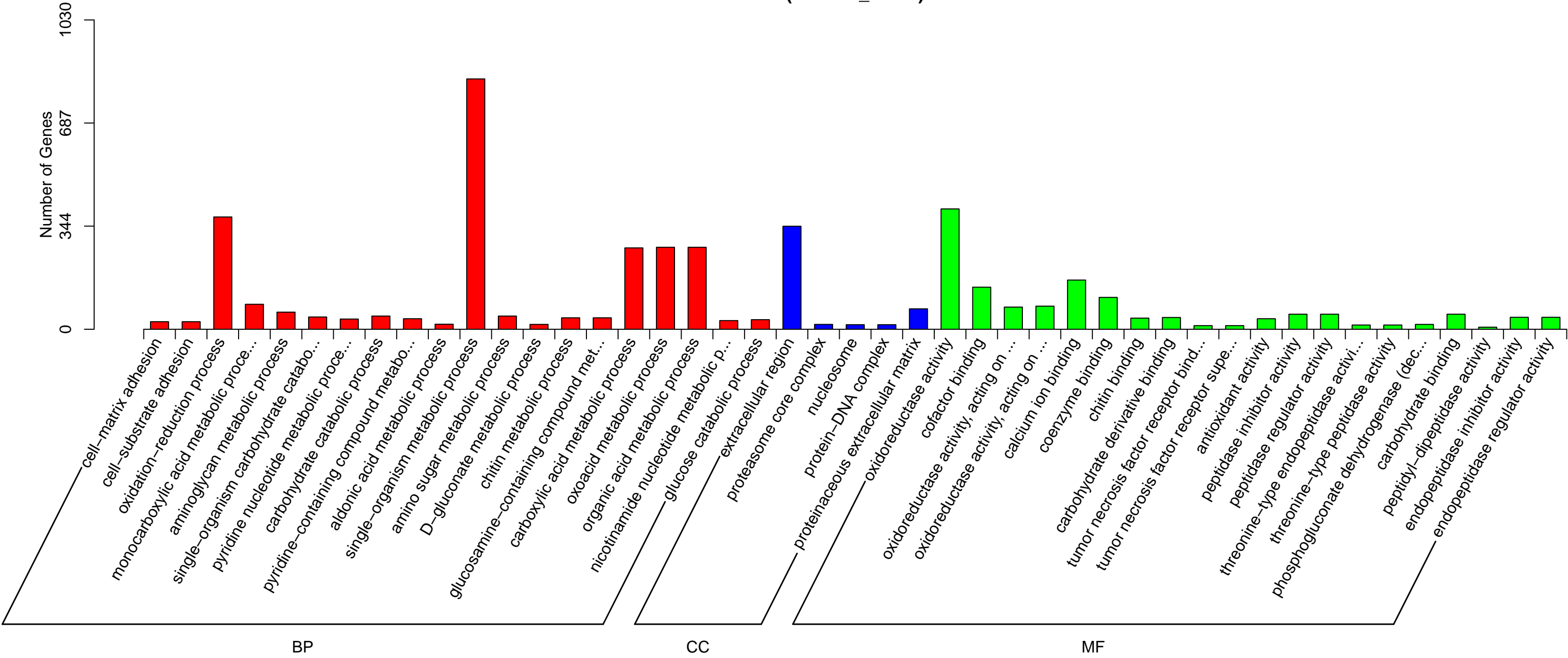

Supplement: Supplemental Material [file supp_g3.116.029314_FigureS2.zip › Figure S1. GO enrichment analysis of developmental comparison groups/C1vsF3_down.DEG_Enriched_GO_classification.pdf]

Enriched GO Terms  
(C1vsF3\_up)

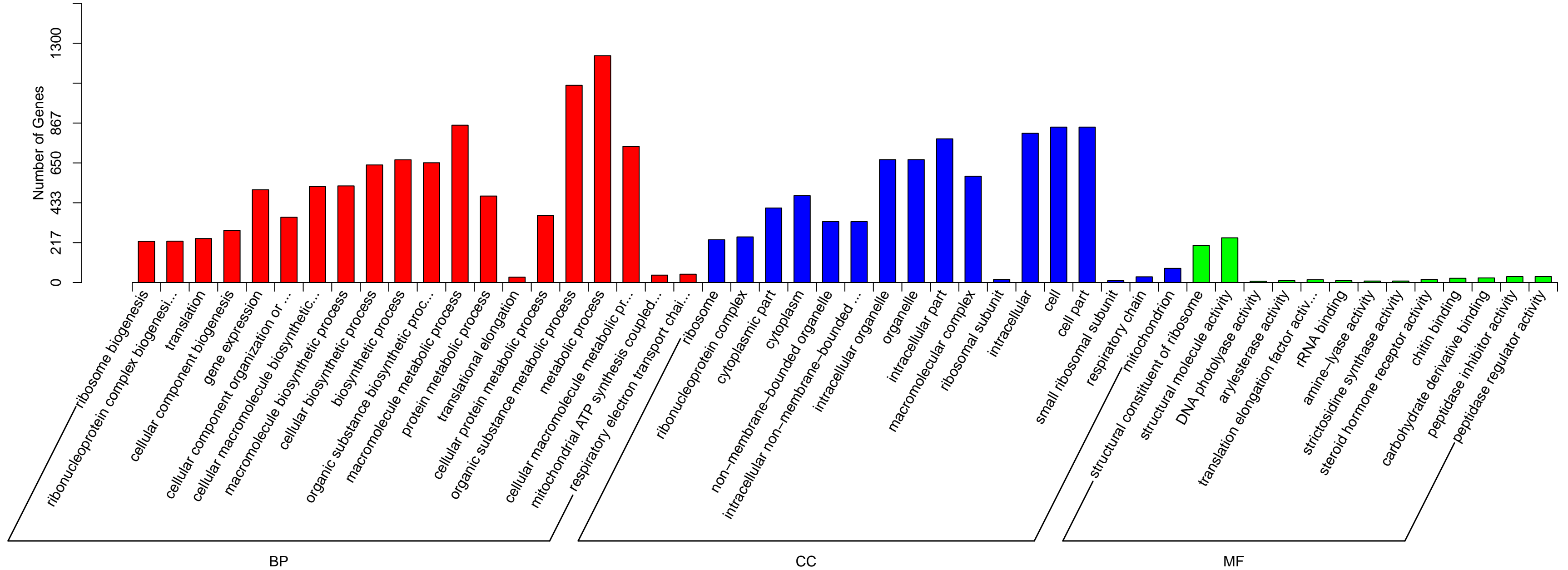

Supplement: Supplemental Material [file supp_g3.116.029314_FigureS2.zip › Figure S1. GO enrichment analysis of developmental comparison groups/C1vsF3_up.DEG_Enriched_GO_classification.pdf]

Enriched GO Terms  
(C1vsJ4)

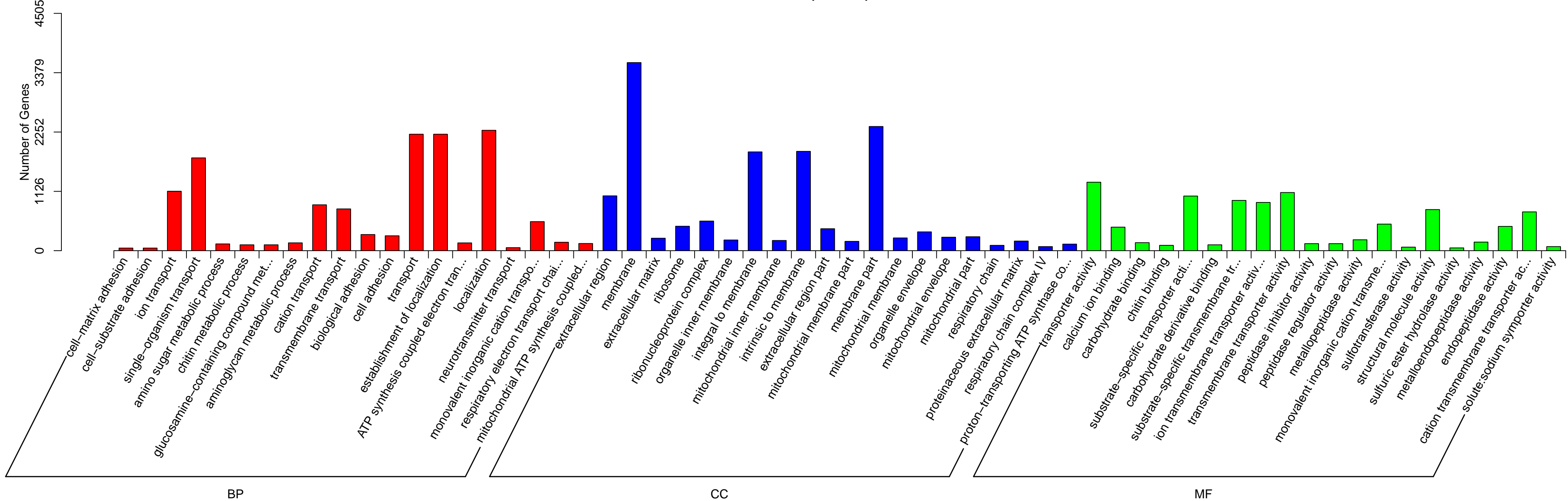

Supplement: Supplemental Material [file supp_g3.116.029314_FigureS2.zip › Figure S1. GO enrichment analysis of developmental comparison groups/C1vsJ4.DEG_Enriched_GO_classification.pdf]

Enriched GO Terms  
(C1vsJ4\_down)

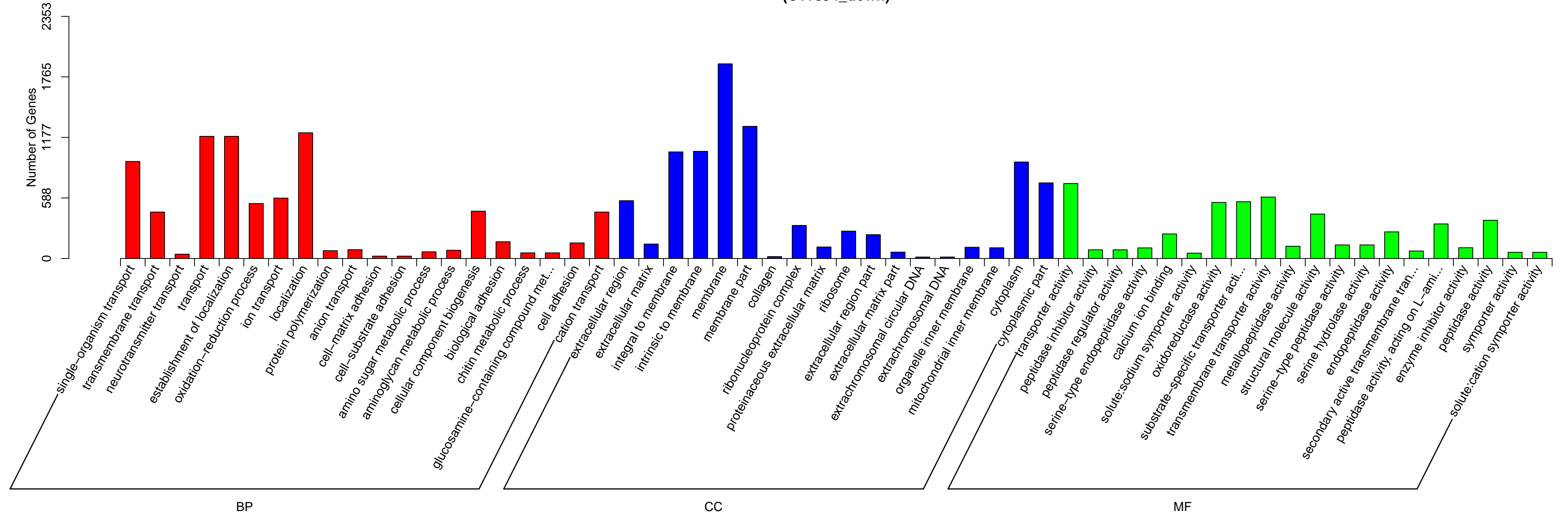

Supplement: Supplemental Material [file supp_g3.116.029314_FigureS2.zip › Figure S1. GO enrichment analysis of developmental comparison groups/C1vsJ4_down.DEG_Enriched_GO_classification.pdf]

Enriched GO Terms  
(C1vsJ4\_up)

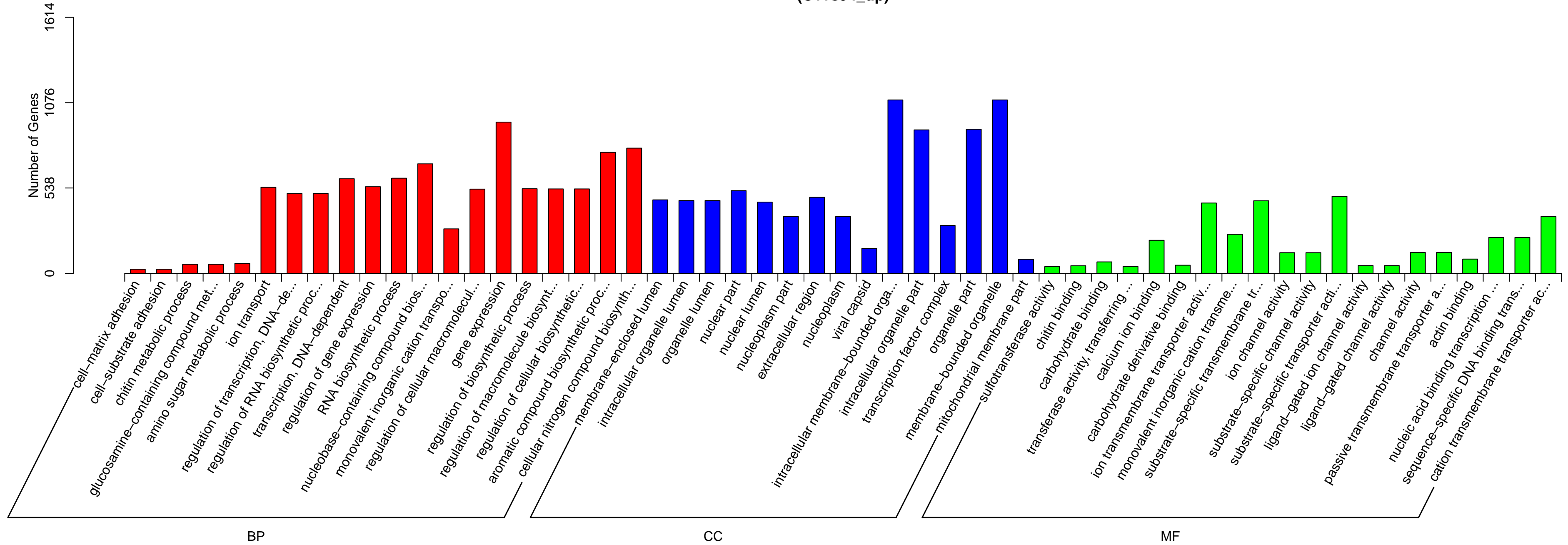

Supplement: Supplemental Material [file supp_g3.116.029314_FigureS2.zip › Figure S1. GO enrichment analysis of developmental comparison groups/C1vsJ4_up.DEG_Enriched_GO_classification.pdf]

Enriched GO Terms  
(C1vsY5)

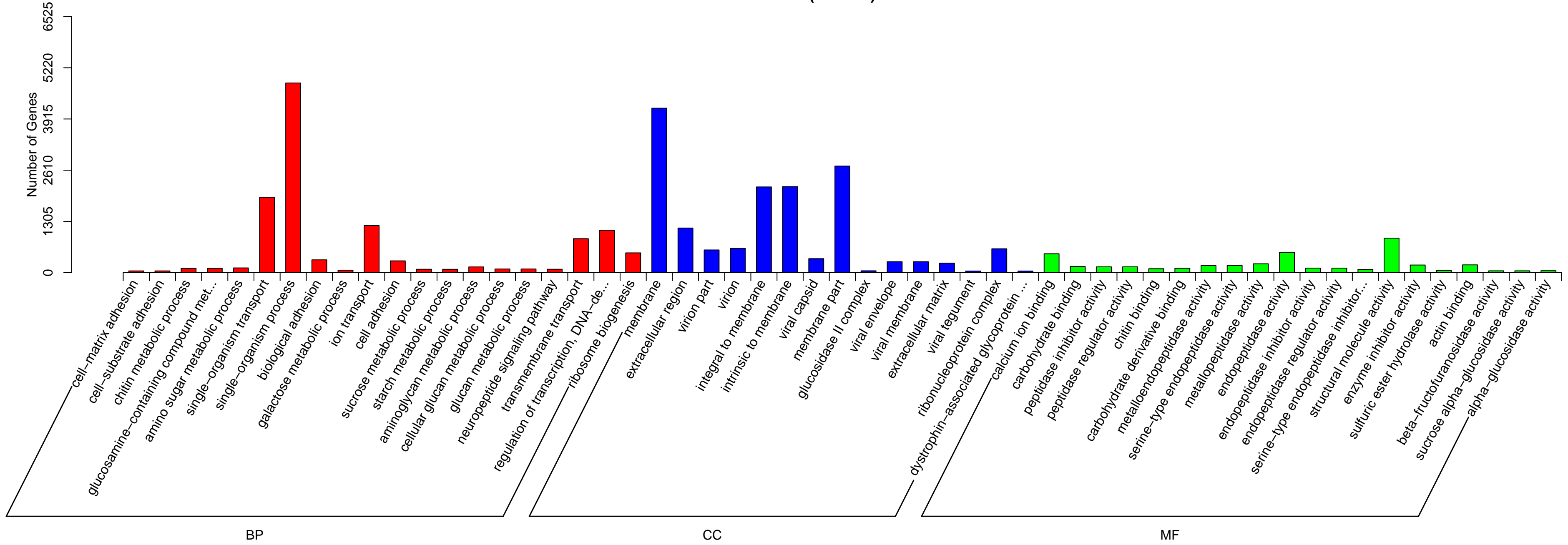

Supplement: Supplemental Material [file supp_g3.116.029314_FigureS2.zip › Figure S1. GO enrichment analysis of developmental comparison groups/C1vsY5.DEG_Enriched_GO_classification.pdf]

Enriched GO Terms  
(C1vsY5\_down)

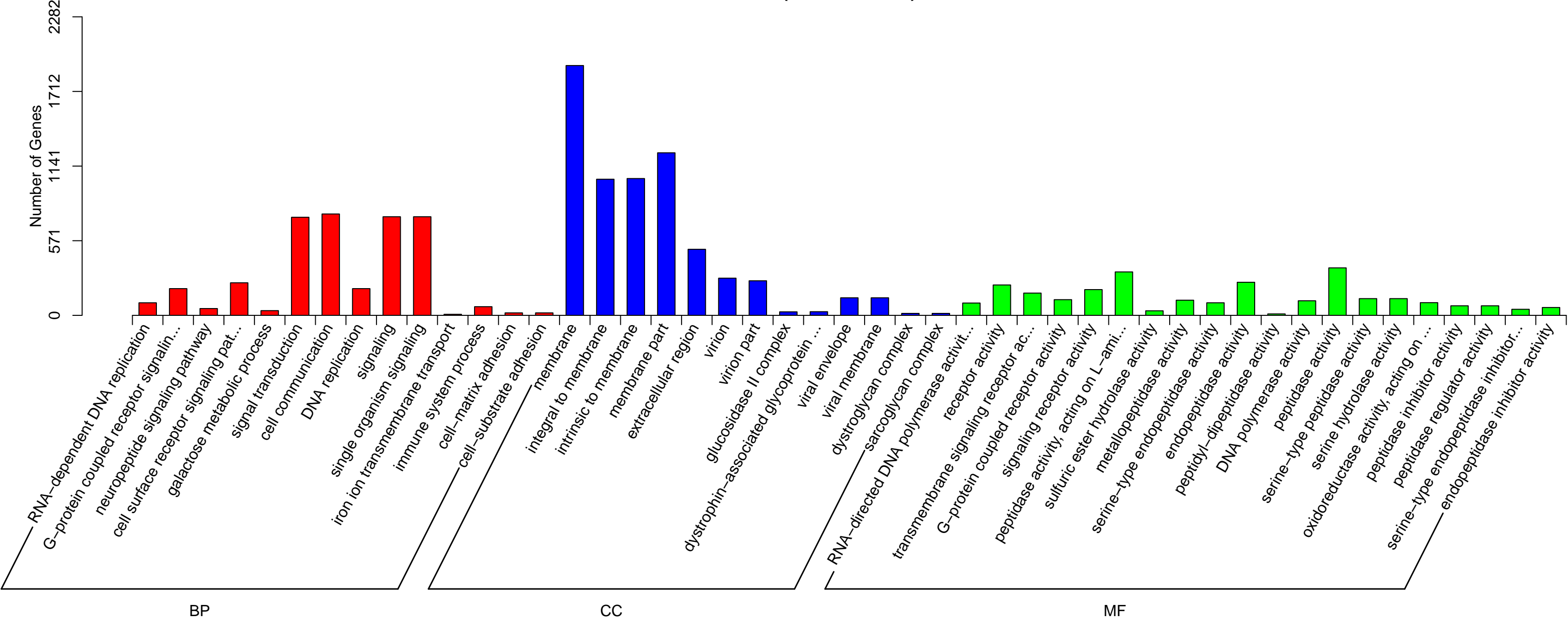

Supplement: Supplemental Material [file supp_g3.116.029314_FigureS2.zip › Figure S1. GO enrichment analysis of developmental comparison groups/C1vsY5_down.DEG_Enriched_GO_classification.pdf]

### Enriched GO Terms (C1vsY5\_up)

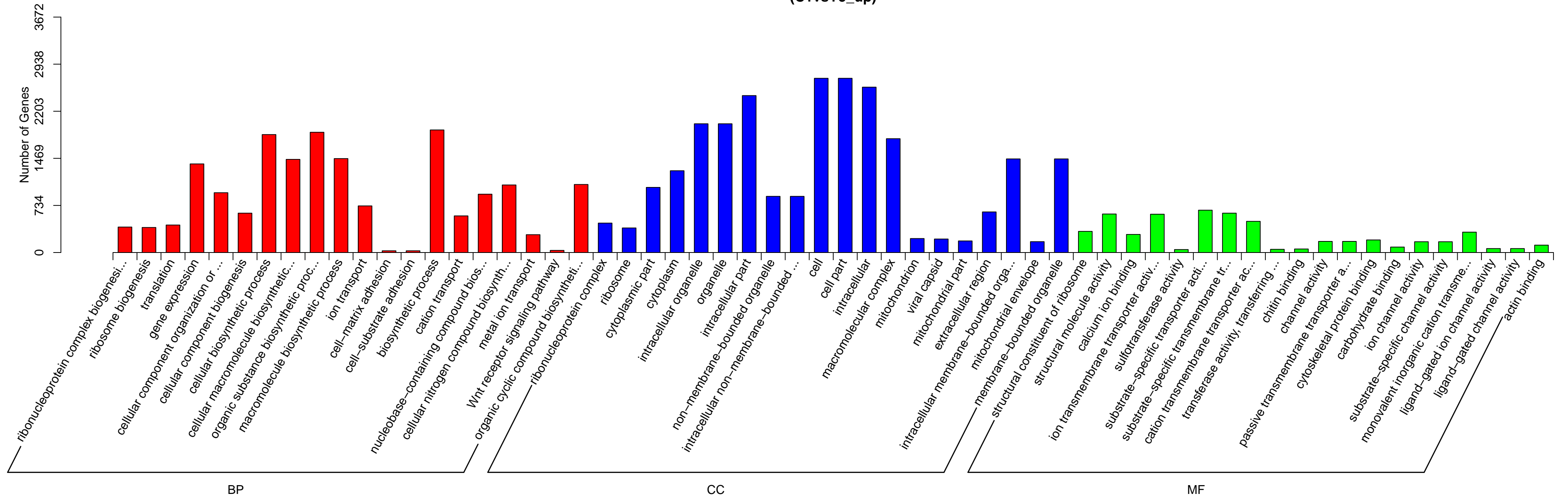

Supplement: Supplemental Material [file supp_g3.116.029314_FigureS2.zip › Figure S1. GO enrichment analysis of developmental comparison groups/C1vsY5_up.DEG_Enriched_GO_classification.pdf]

Enriched GO Terms  
(D2vsF3)

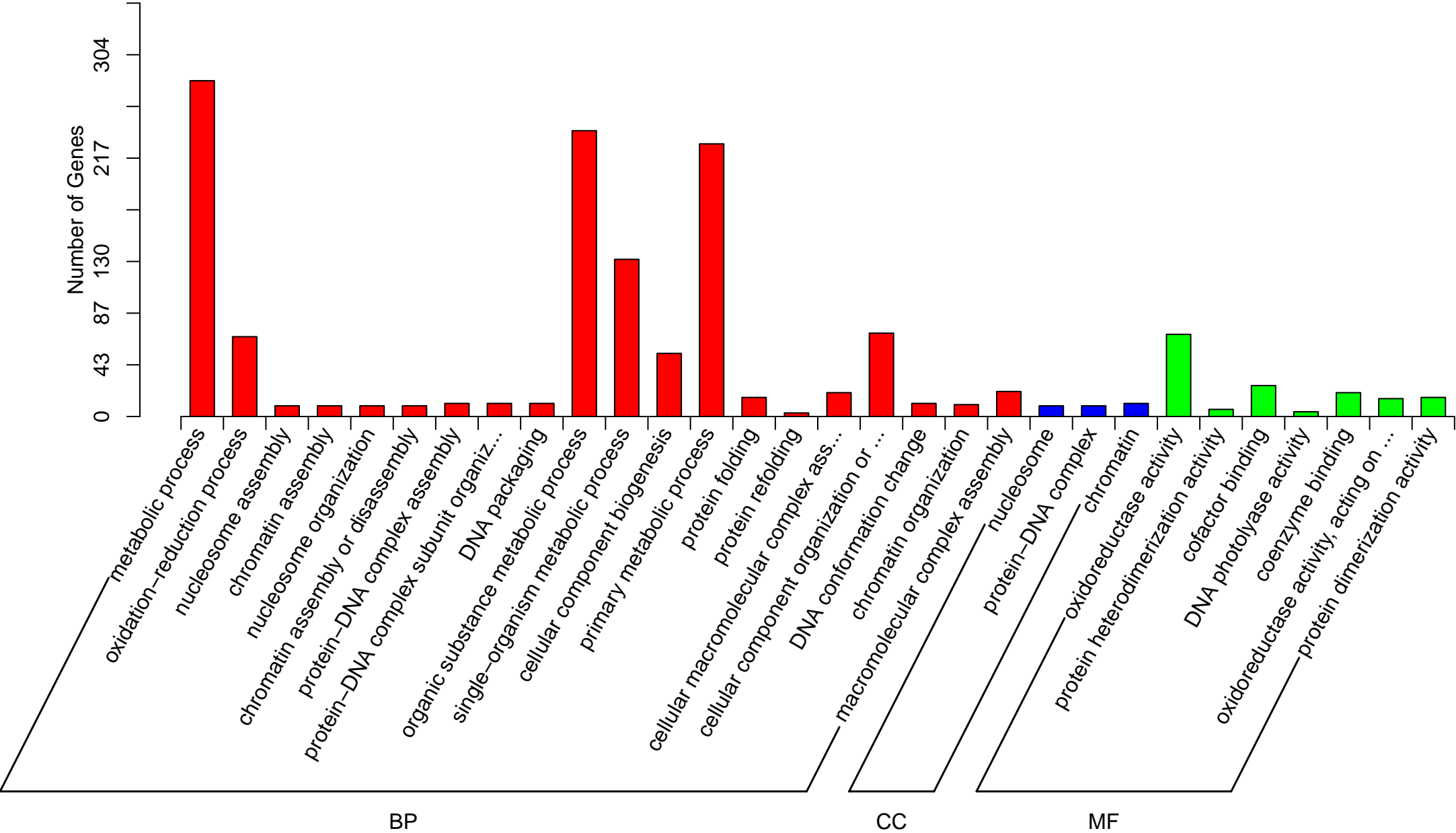

Supplement: Supplemental Material [file supp_g3.116.029314_FigureS2.zip › Figure S1. GO enrichment analysis of developmental comparison groups/D2vsF3.DEG_Enriched_GO_classification.pdf]

Enriched GO Terms  
(D2vsF3\_up)

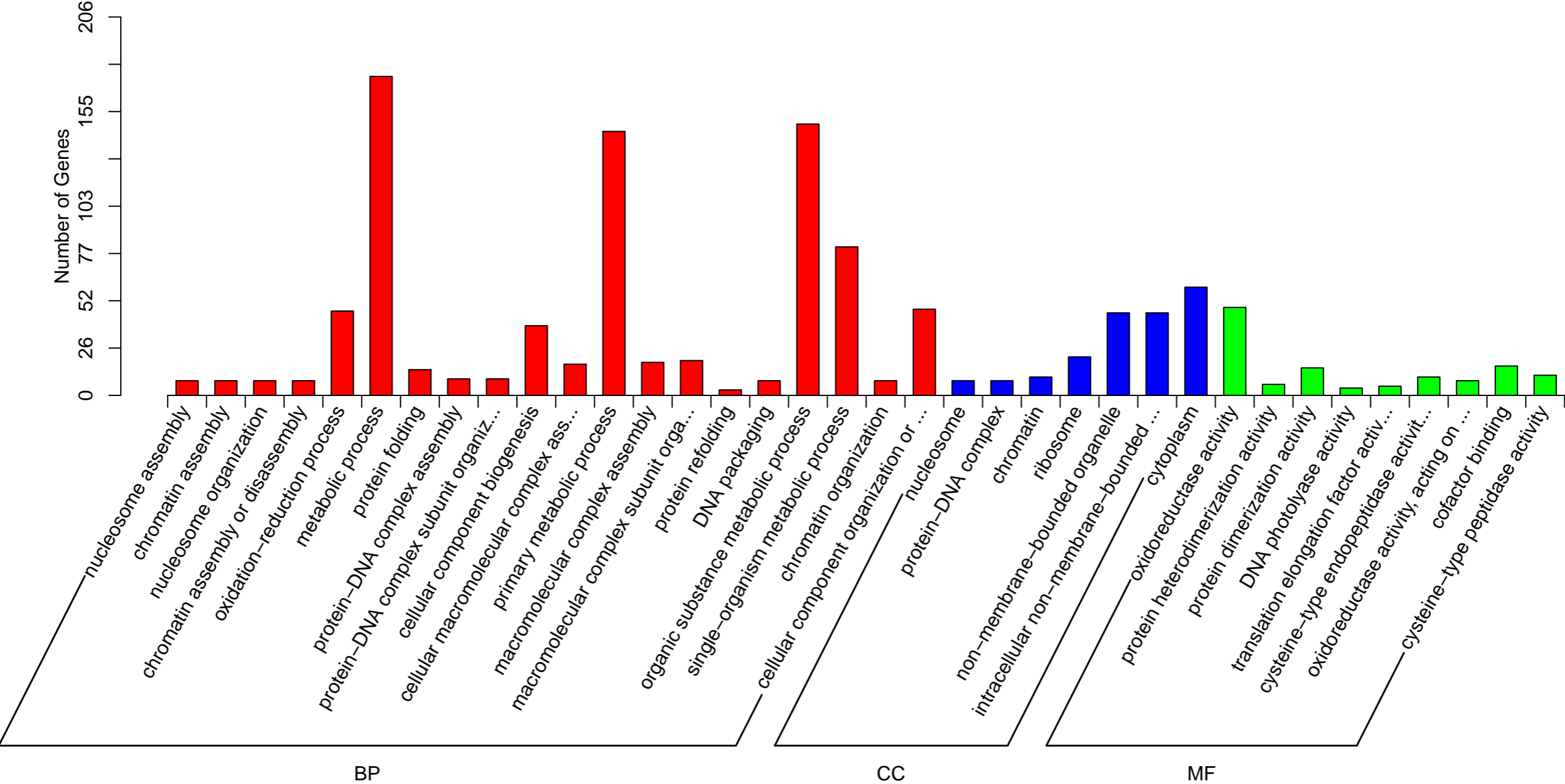

Supplement: Supplemental Material [file supp_g3.116.029314_FigureS2.zip › Figure S1. GO enrichment analysis of developmental comparison groups/D2vsF3_up.DEG_Enriched_GO_classification.pdf]

Enriched GO Terms  
(D2vsJ4)

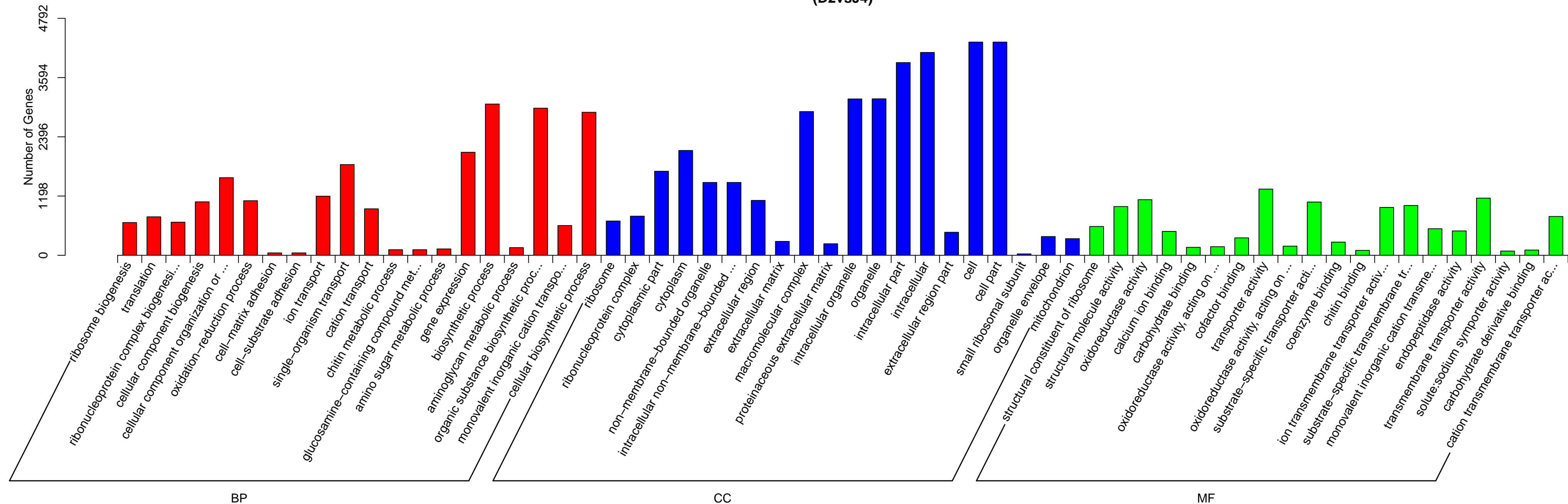

Supplement: Supplemental Material [file supp_g3.116.029314_FigureS2.zip › Figure S1. GO enrichment analysis of developmental comparison groups/D2vsJ4.DEG_Enriched_GO_classification.pdf]

Enriched GO Terms  
(D2vsJ4\_down)

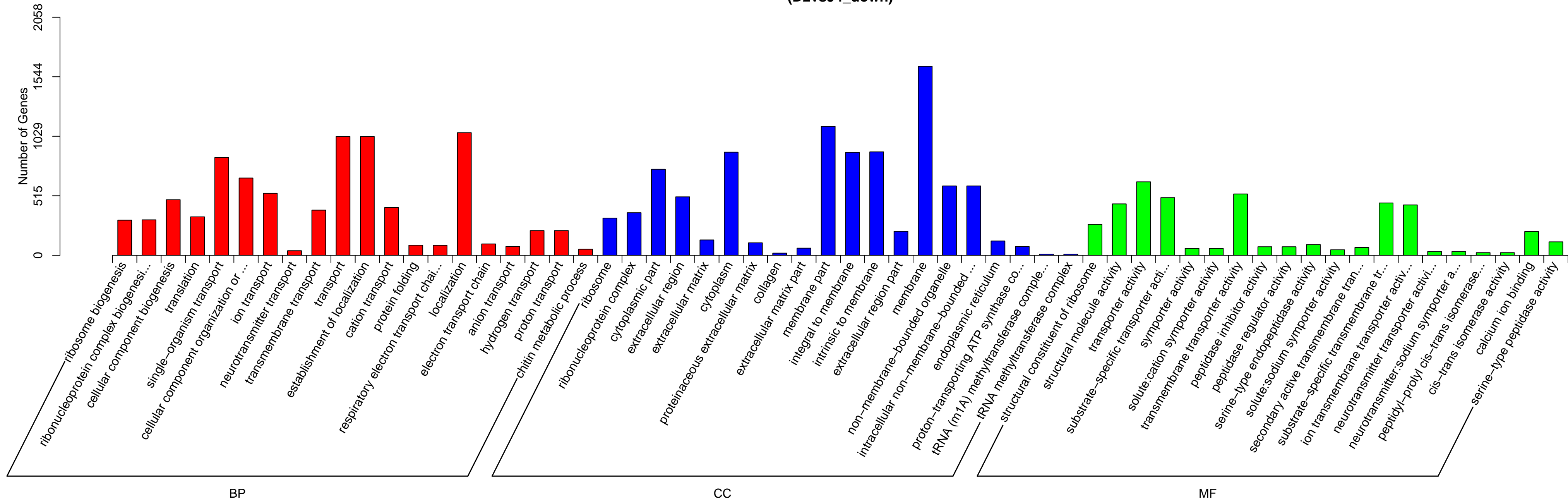

Supplement: Supplemental Material [file supp_g3.116.029314_FigureS2.zip › Figure S1. GO enrichment analysis of developmental comparison groups/D2vsJ4_down.DEG_Enriched_GO_classification.pdf]

Enriched GO Terms  
(D2vsJ4\_up)

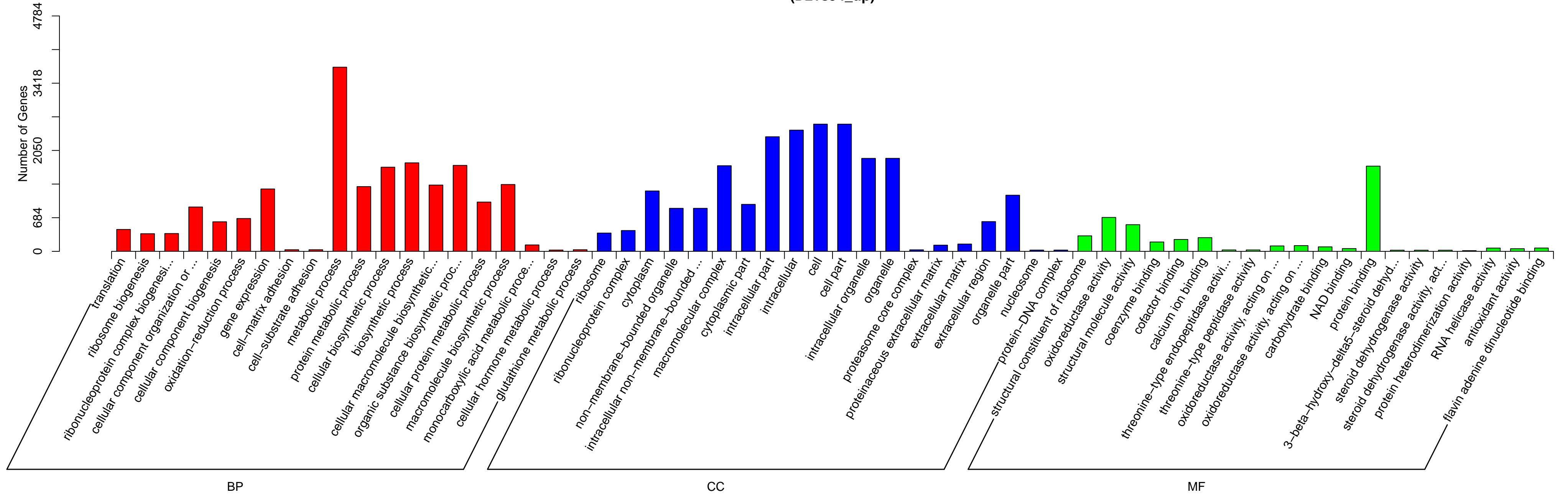

Supplement: Supplemental Material [file supp_g3.116.029314_FigureS2.zip › Figure S1. GO enrichment analysis of developmental comparison groups/D2vsJ4_up.DEG_Enriched_GO_classification.pdf]

Enriched GO Terms  
(D2vsY5)

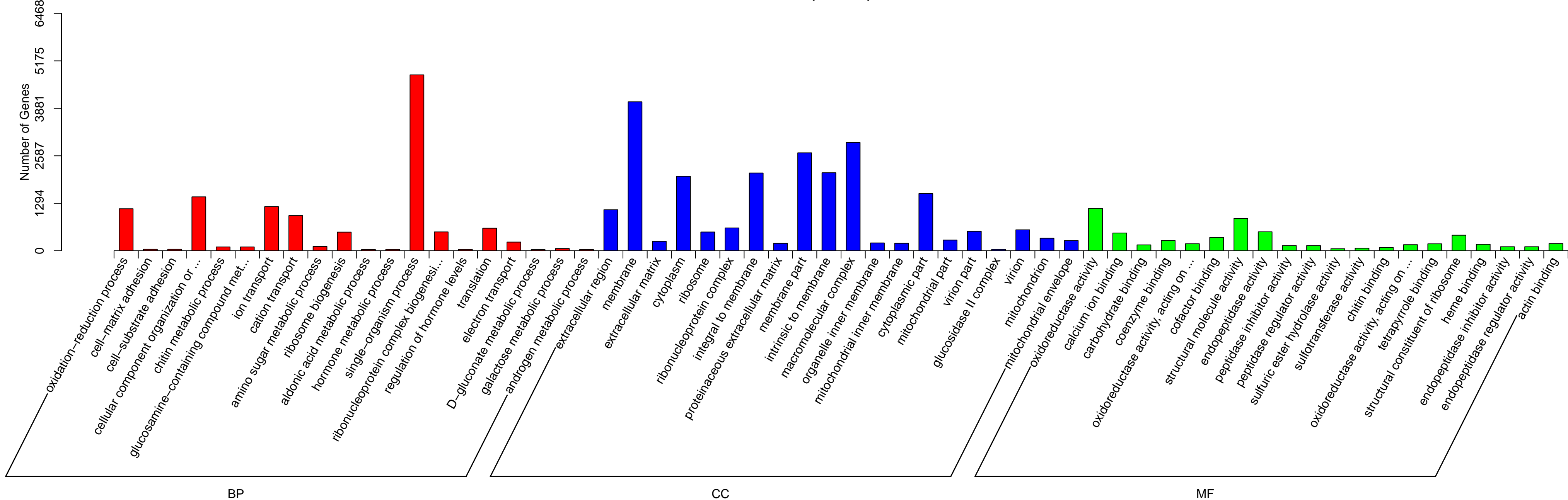

Supplement: Supplemental Material [file supp_g3.116.029314_FigureS2.zip › Figure S1. GO enrichment analysis of developmental comparison groups/D2vsY5.DEG_Enriched_GO_classification.pdf]

Enriched GO Terms  
(D2vsY5\_down)

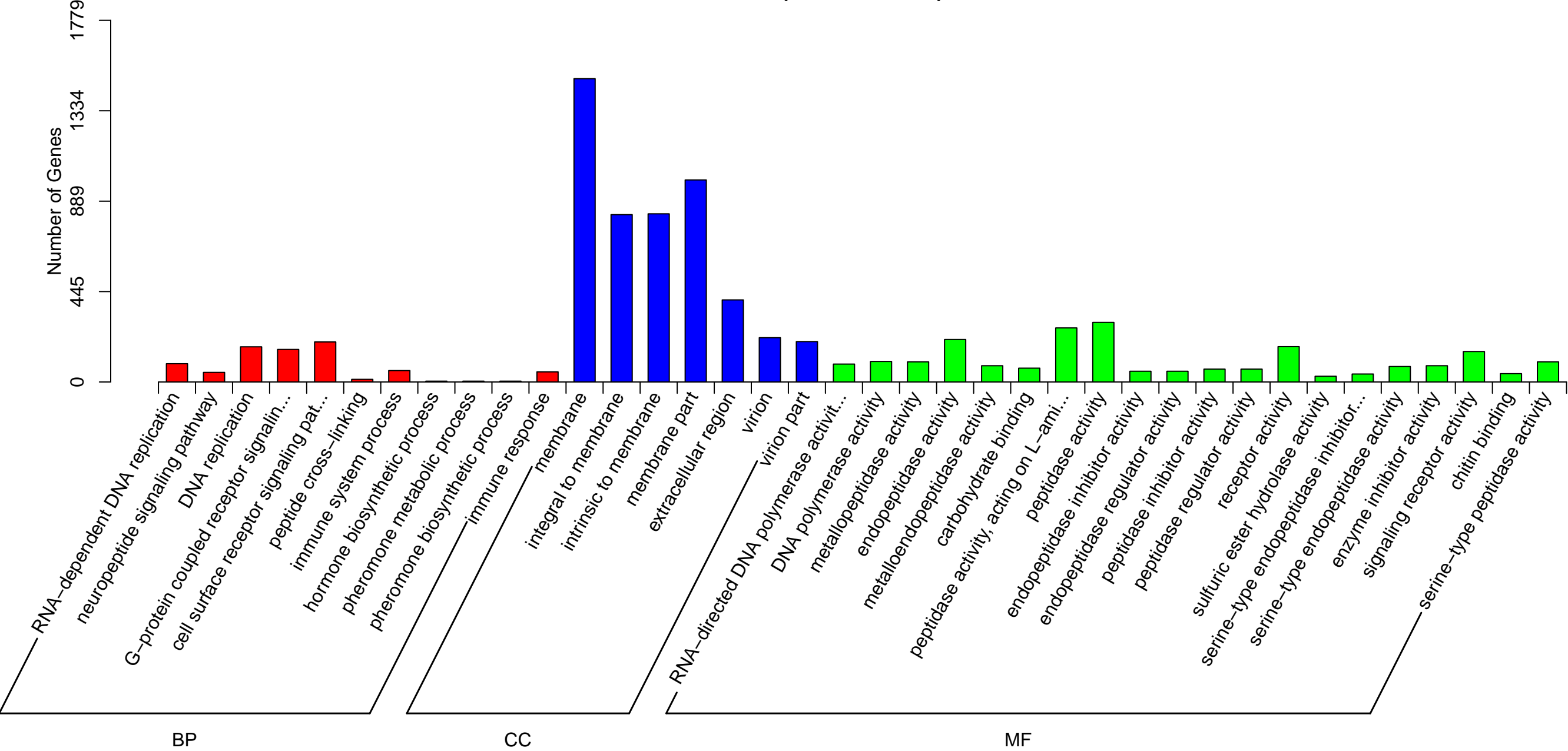

Supplement: Supplemental Material [file supp_g3.116.029314_FigureS2.zip › Figure S1. GO enrichment analysis of developmental comparison groups/D2vsY5_down.DEG_Enriched_GO_classification.pdf]

Enriched GO Terms  
(D2vsY5\_up)

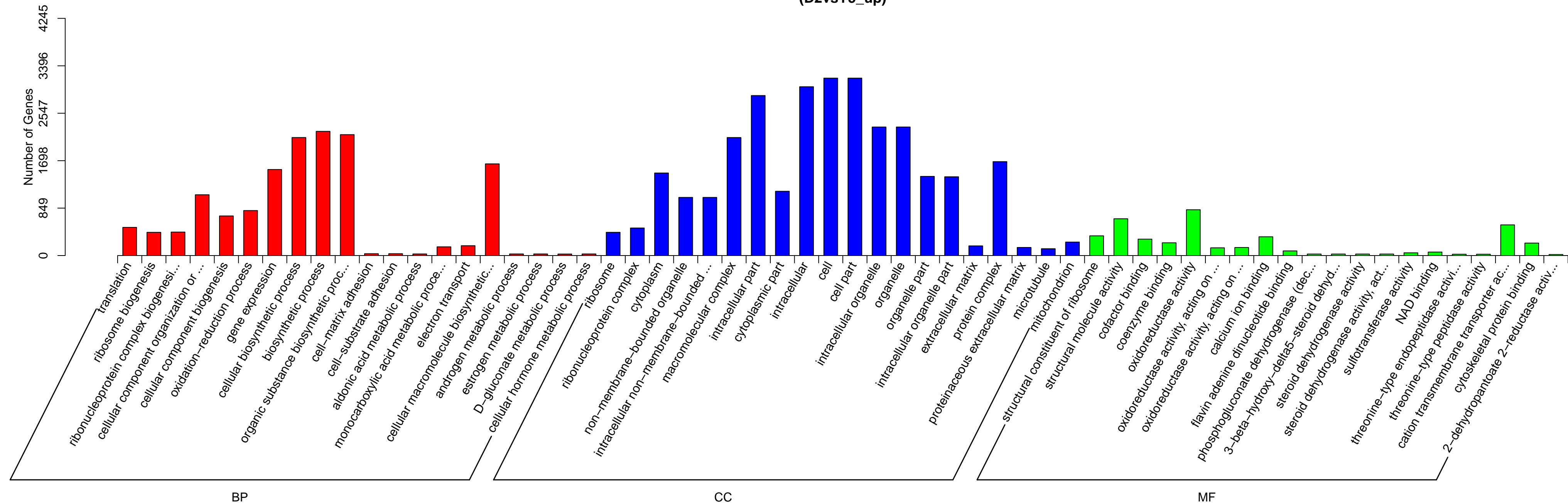

Supplement: Supplemental Material [file supp_g3.116.029314_FigureS2.zip › Figure S1. GO enrichment analysis of developmental comparison groups/D2vsY5_up.DEG_Enriched_GO_classification.pdf]

Enriched GO Terms  
(F3vsJ4)

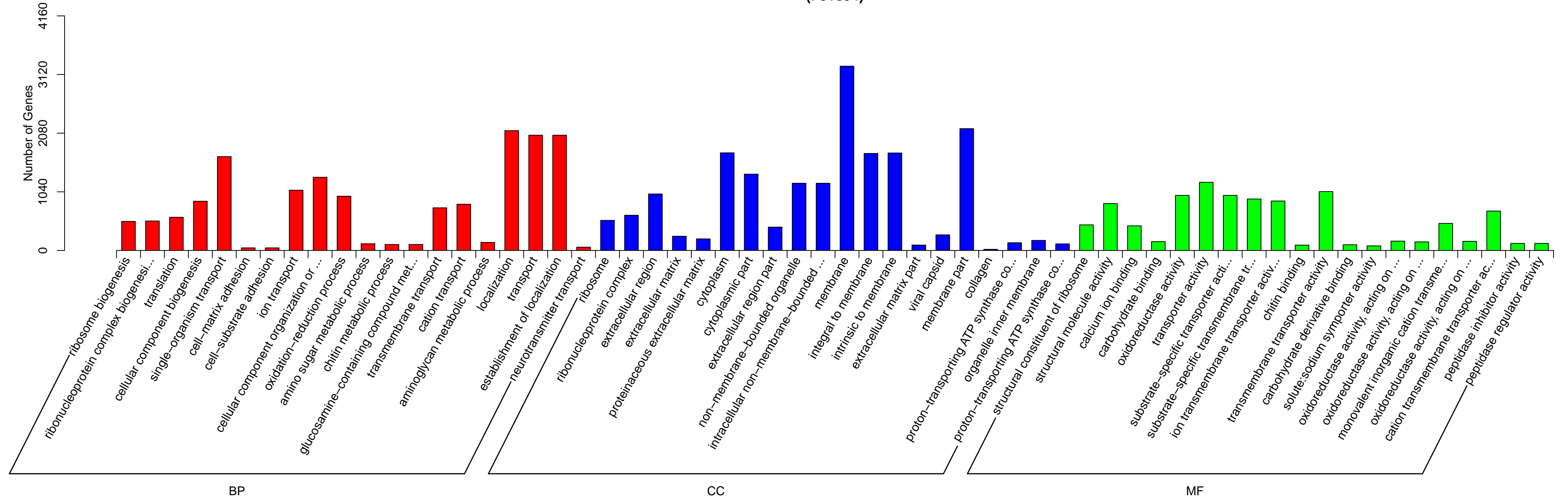

Supplement: Supplemental Material [file supp_g3.116.029314_FigureS2.zip › Figure S1. GO enrichment analysis of developmental comparison groups/F3vsJ4.DEG_Enriched_GO_classification.pdf]

Enriched GO Terms  
(F3vsJ4\_down)

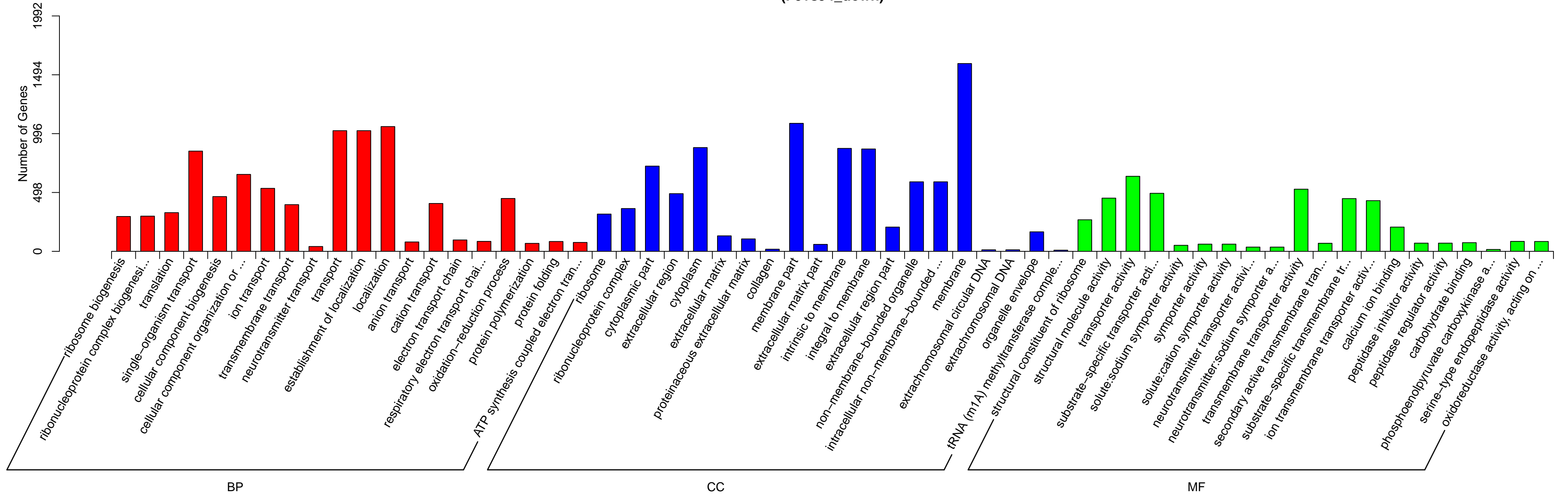

Supplement: Supplemental Material [file supp_g3.116.029314_FigureS2.zip › Figure S1. GO enrichment analysis of developmental comparison groups/F3vsJ4_down.DEG_Enriched_GO_classification.pdf]

Enriched GO Terms  
(F3vsJ4\_up)

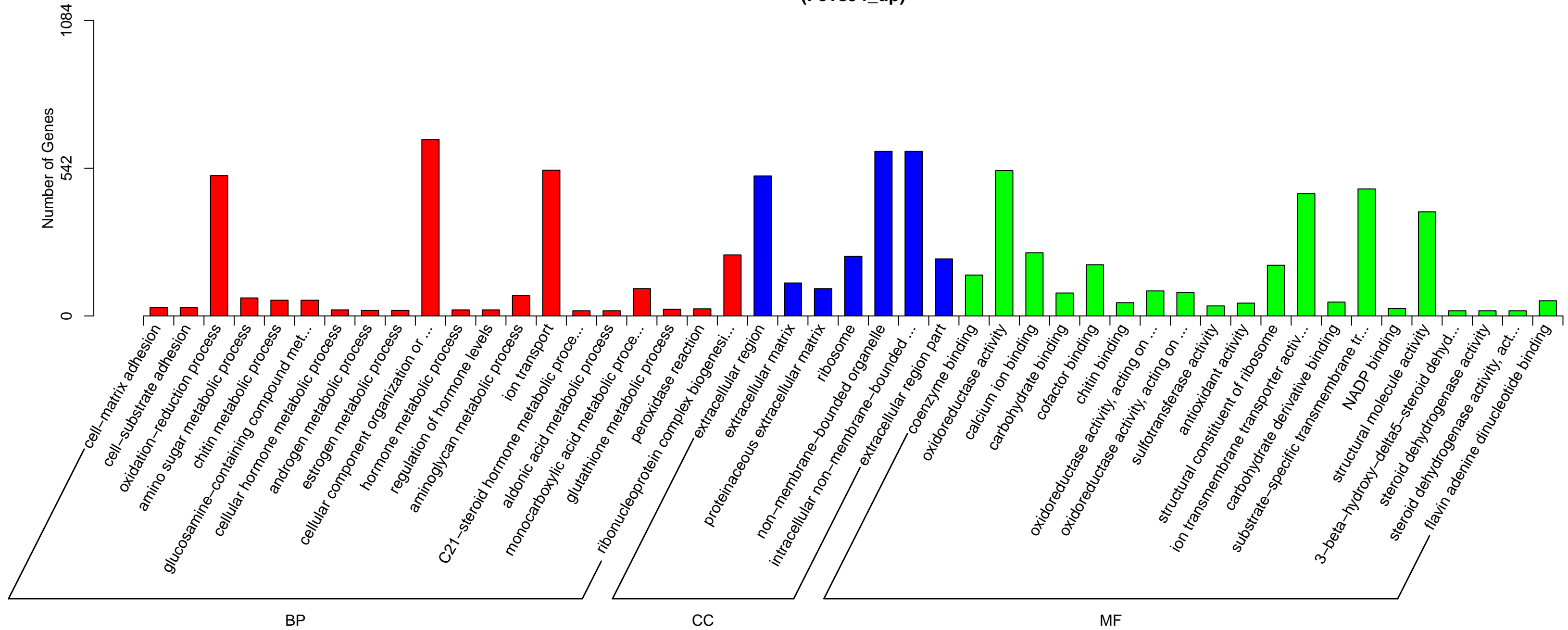

Supplement: Supplemental Material [file supp_g3.116.029314_FigureS2.zip › Figure S1. GO enrichment analysis of developmental comparison groups/F3vsJ4_up.DEG_Enriched_GO_classification.pdf]

Enriched GO Terms  
(F3vsY5)

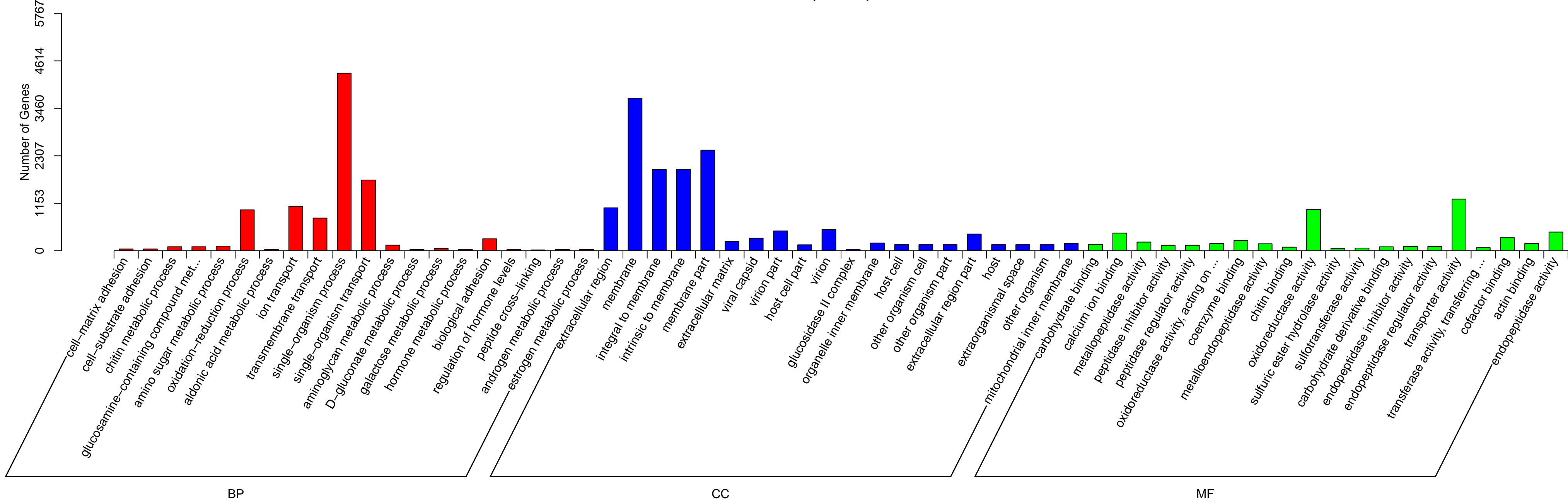

Supplement: Supplemental Material [file supp_g3.116.029314_FigureS2.zip › Figure S1. GO enrichment analysis of developmental comparison groups/F3vsY5.DEG_Enriched_GO_classification.pdf]

Enriched GO Terms  
(F3vsY5\_down)

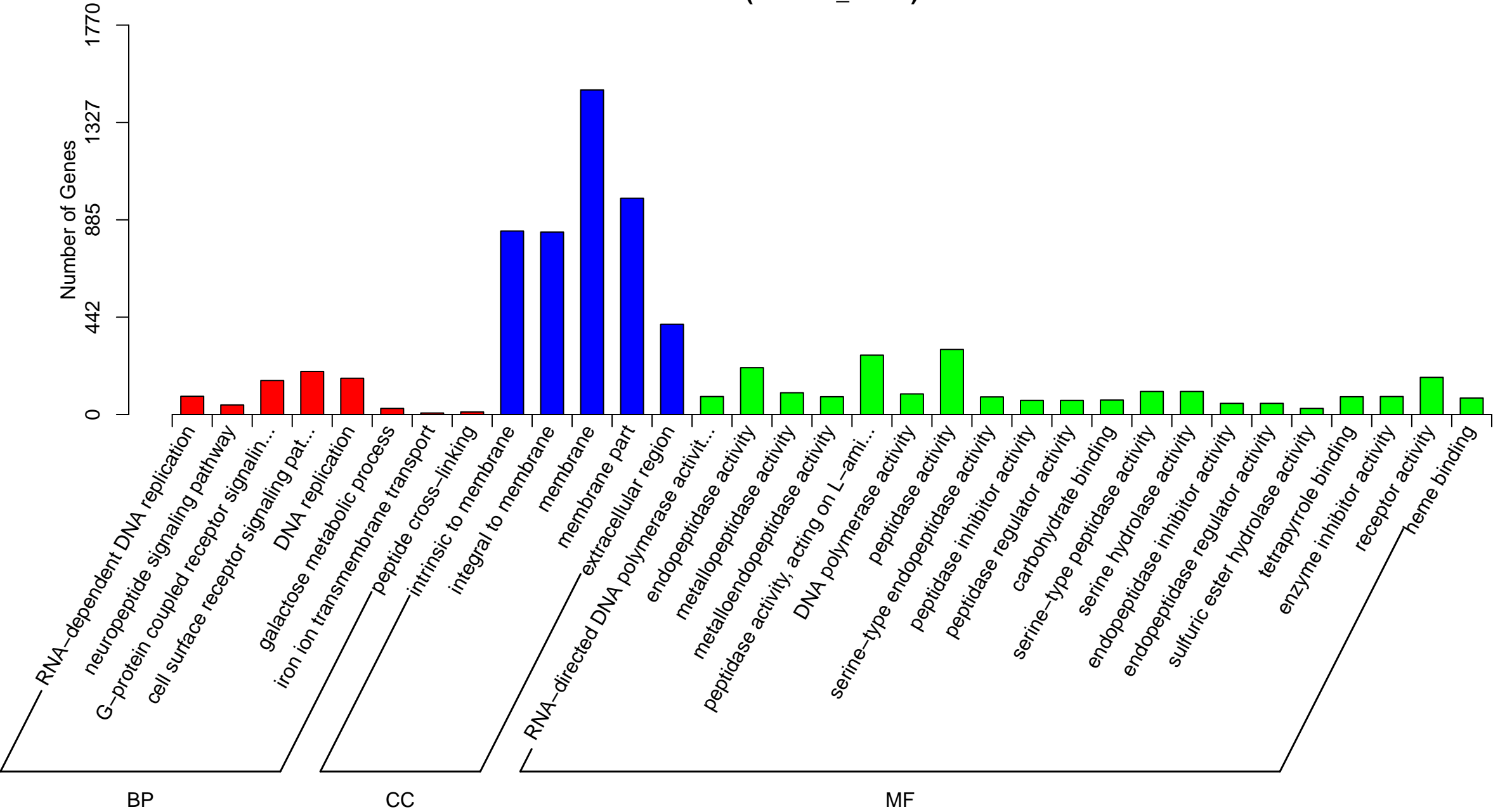

Supplement: Supplemental Material [file supp_g3.116.029314_FigureS2.zip › Figure S1. GO enrichment analysis of developmental comparison groups/F3vsY5_down.DEG_Enriched_GO_classification.pdf]

Enriched GO Terms  
(F3vsY5\_up)

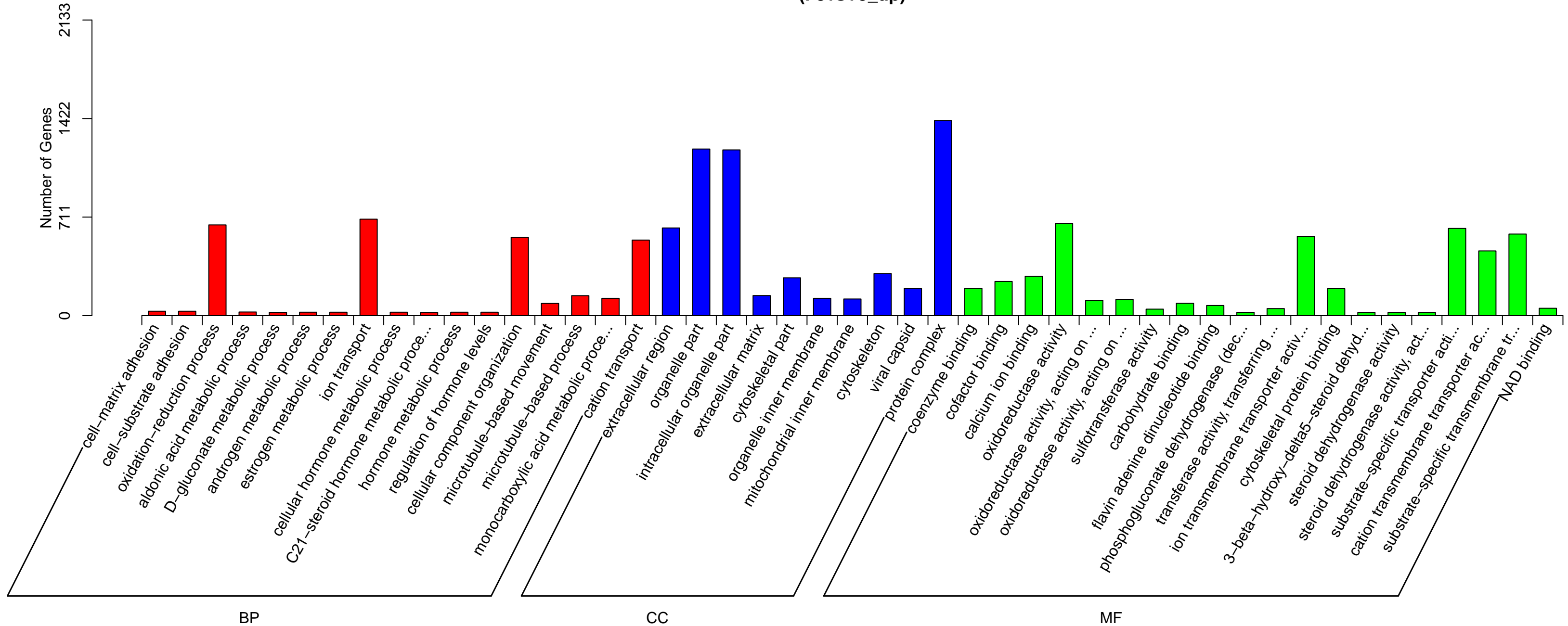

Supplement: Supplemental Material [file supp_g3.116.029314_FigureS2.zip › Figure S1. GO enrichment analysis of developmental comparison groups/F3vsY5_up.DEG_Enriched_GO_classification.pdf]

Enriched GO Terms  
(J4vsY5)

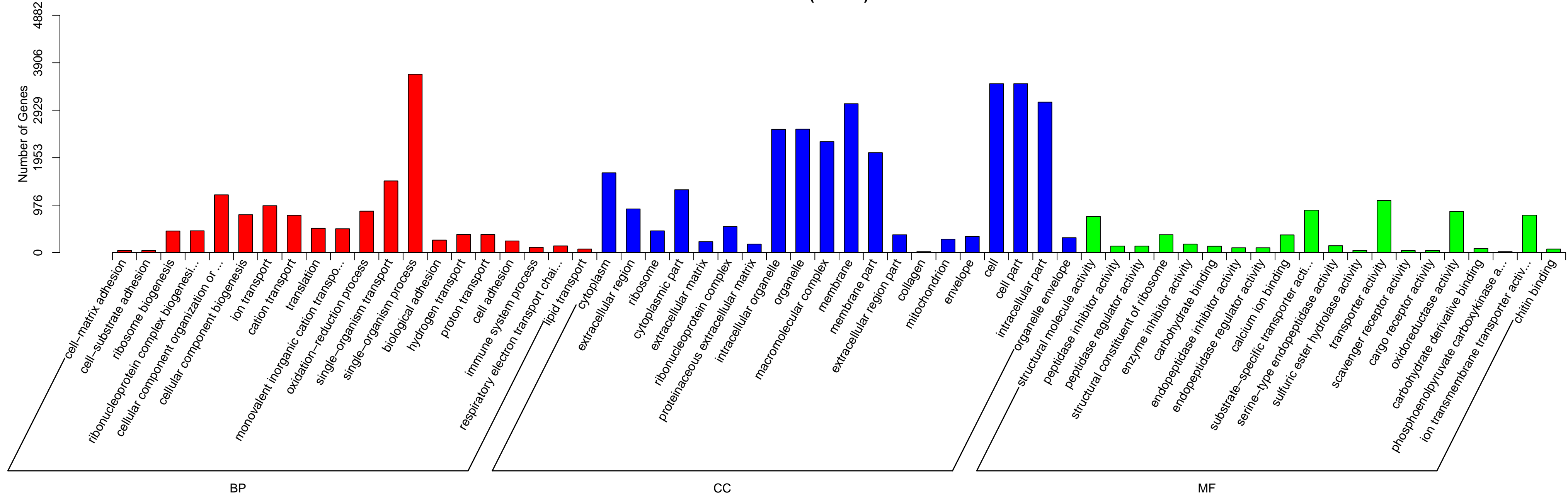

Supplement: Supplemental Material [file supp_g3.116.029314_FigureS2.zip › Figure S1. GO enrichment analysis of developmental comparison groups/J4vsY5.DEG_Enriched_GO_classification.pdf]

Enriched GO Terms  
(J4vsY5\_down)

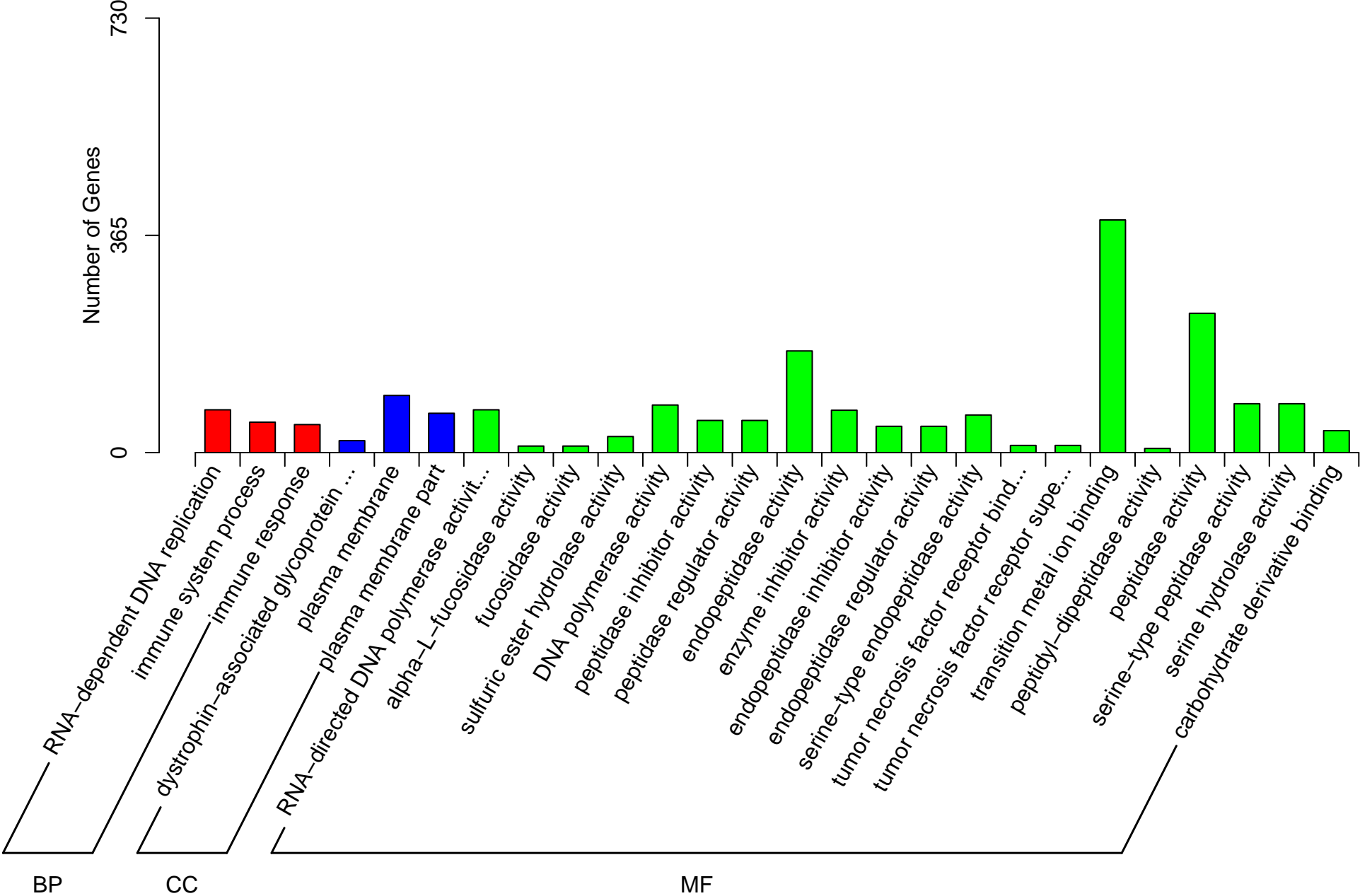

Supplement: Supplemental Material [file supp_g3.116.029314_FigureS2.zip › Figure S1. GO enrichment analysis of developmental comparison groups/J4vsY5_down.DEG_Enriched_GO_classification.pdf]

Enriched GO Terms  
(J4vsY5\_up)

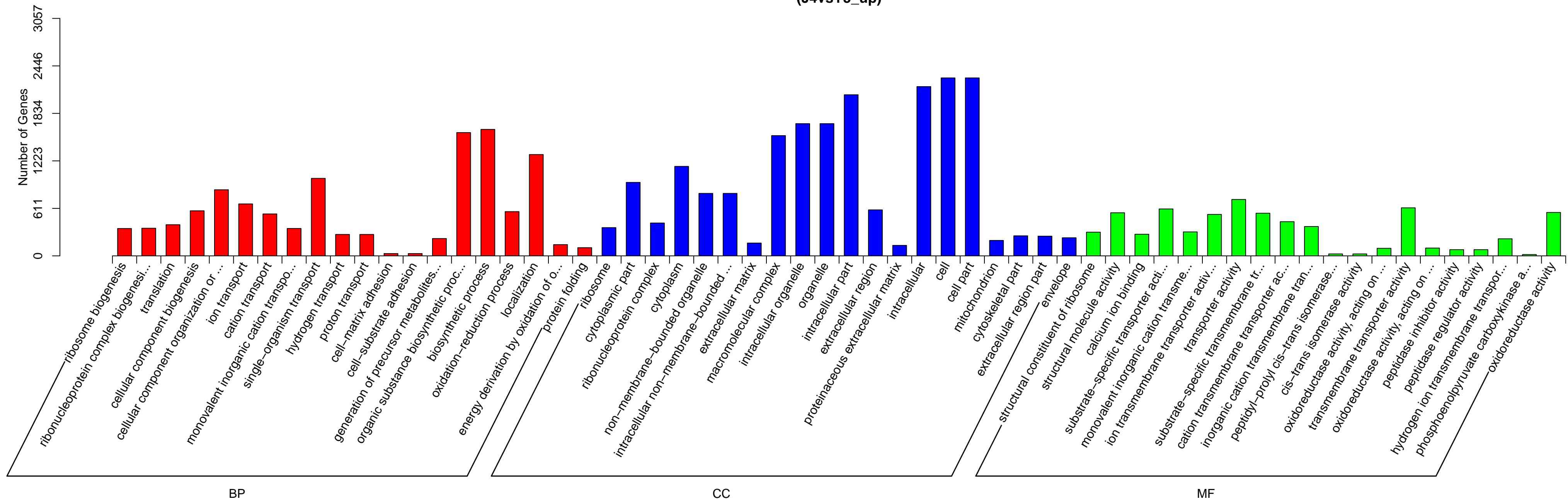

Supplement: Supplemental Material [file supp_g3.116.029314_FigureS2.zip › Figure S1. GO enrichment analysis of developmental comparison groups/J4vsY5_up.DEG_Enriched_GO_classification.pdf]

# Statistics of Pathway Enrichment

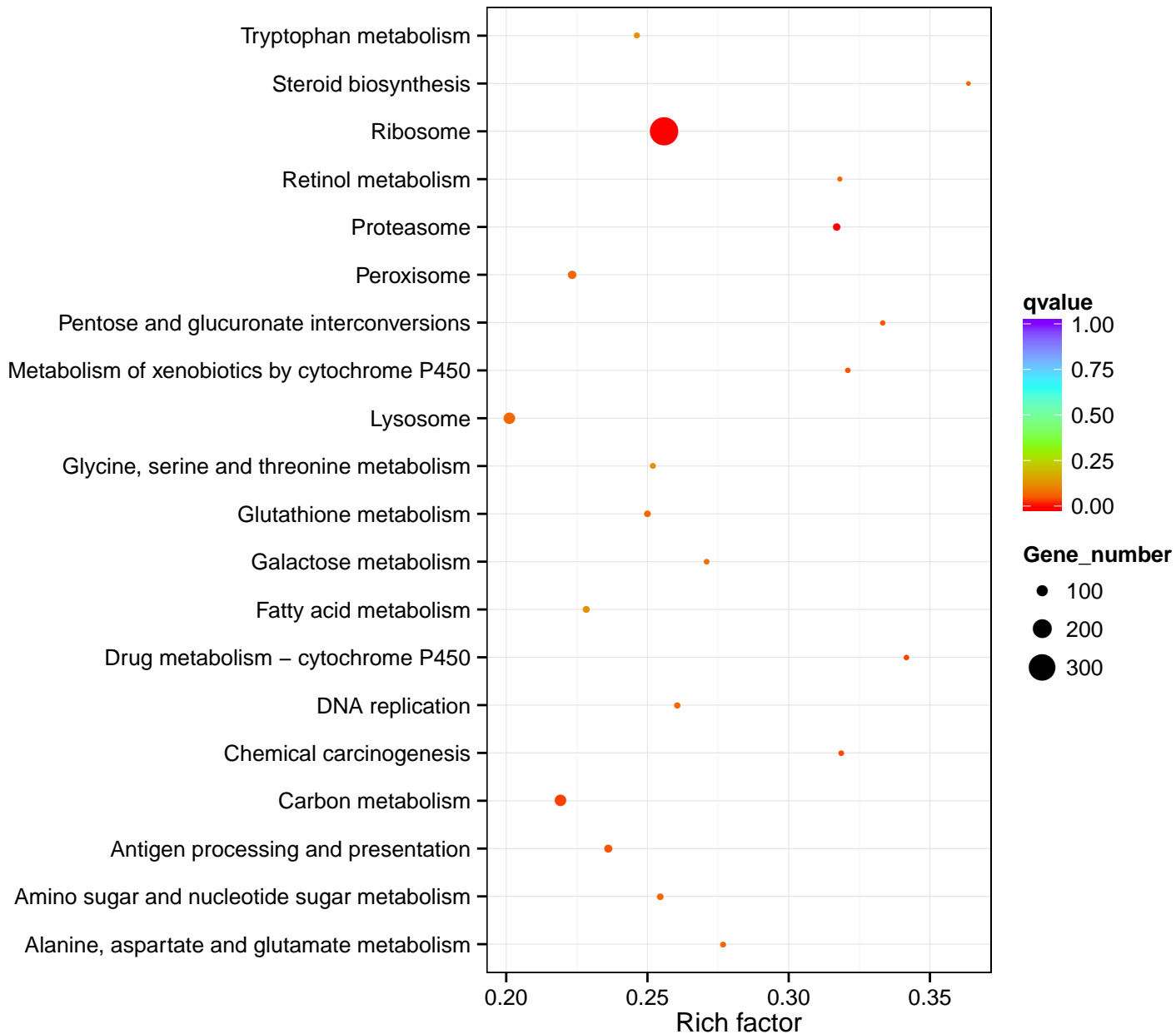

Supplement: Supplemental Material [file supp_g3.116.029314_FigureS3.zip › Figure S2. KEGG enrichment analysis of other developmental comparison groups/C1vsD2.DEG_enriched_KEGG_pathway_scatterplot.pdf]

# Statistics of Pathway Enrichment

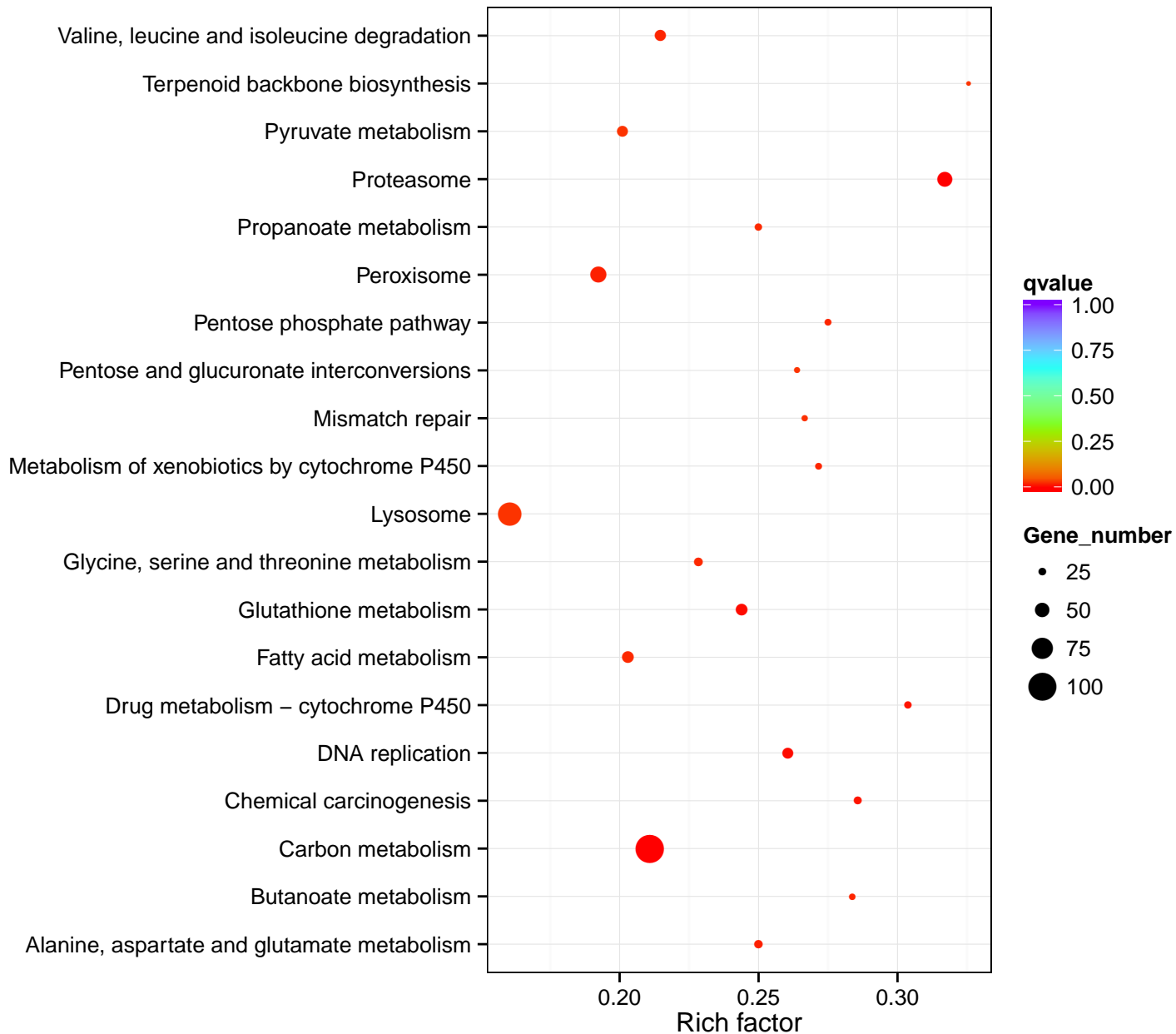

Supplement: Supplemental Material [file supp_g3.116.029314_FigureS3.zip › Figure S2. KEGG enrichment analysis of other developmental comparison groups/C1vsD2_down.DEG_enriched_KEGG_pathway_scatterplot.pdf]

# Statistics of Pathway Enrichment

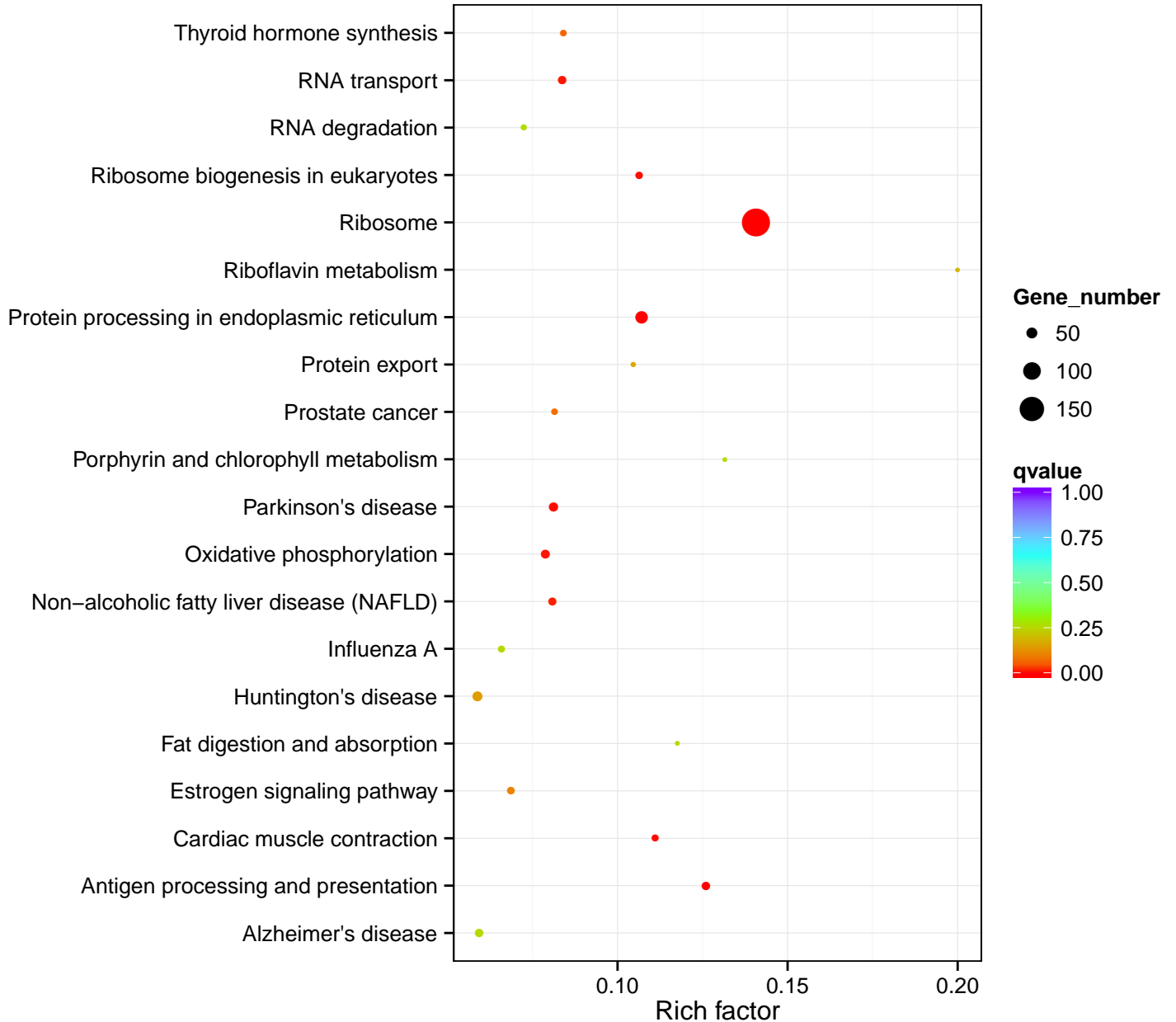

Supplement: Supplemental Material [file supp_g3.116.029314_FigureS3.zip › Figure S2. KEGG enrichment analysis of other developmental comparison groups/C1vsD2_up.DEG_enriched_KEGG_pathway_scatterplot.pdf]

# Statistics of Pathway Enrichment

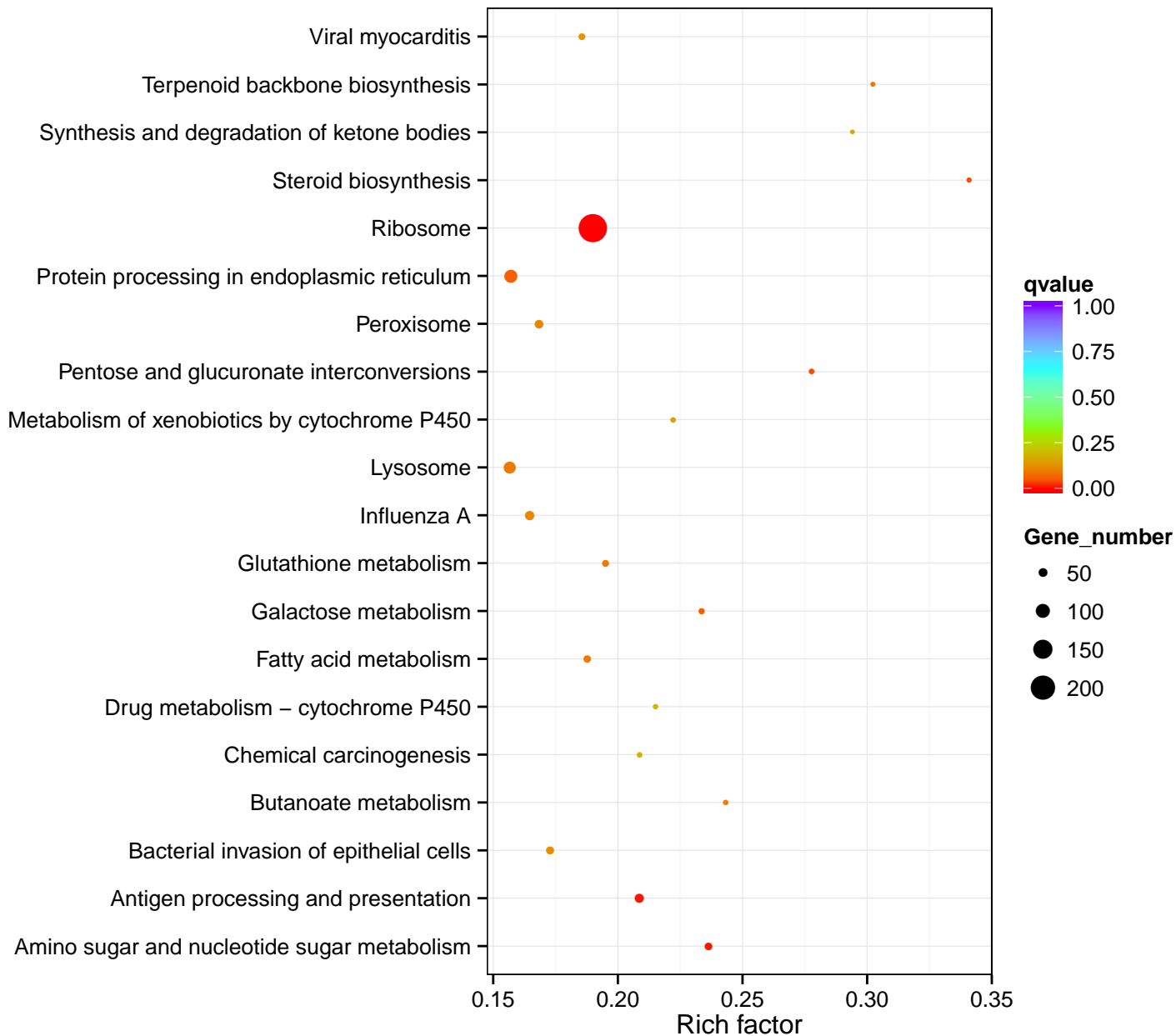

Supplement: Supplemental Material [file supp_g3.116.029314_FigureS3.zip › Figure S2. KEGG enrichment analysis of other developmental comparison groups/C1vsF3.DEG_enriched_KEGG_pathway_scatterplot.pdf]

# Statistics of Pathway Enrichment

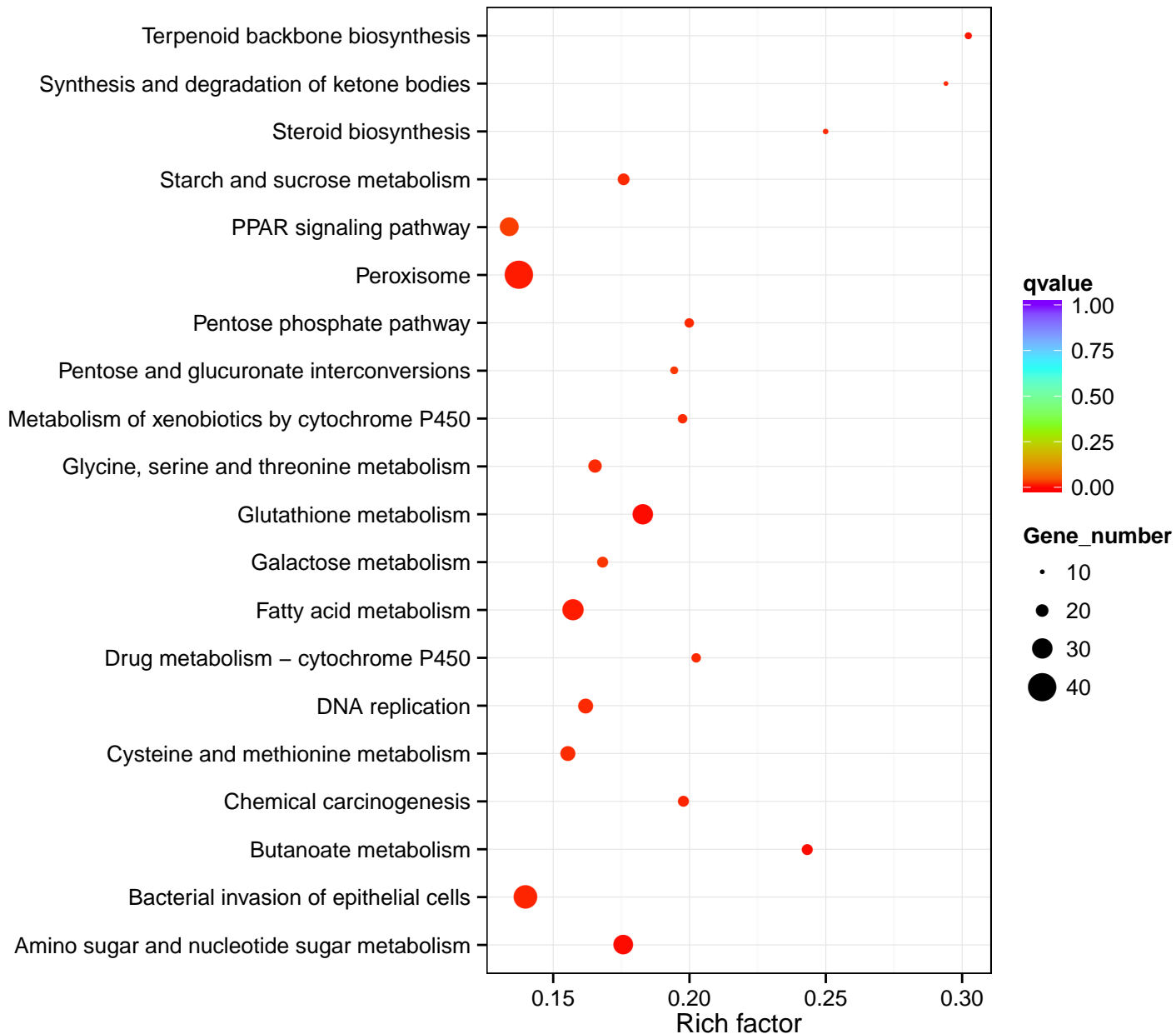

Supplement: Supplemental Material [file supp_g3.116.029314_FigureS3.zip › Figure S2. KEGG enrichment analysis of other developmental comparison groups/C1vsF3_down.DEG_enriched_KEGG_pathway_scatterplot.pdf]

# Statistics of Pathway Enrichment

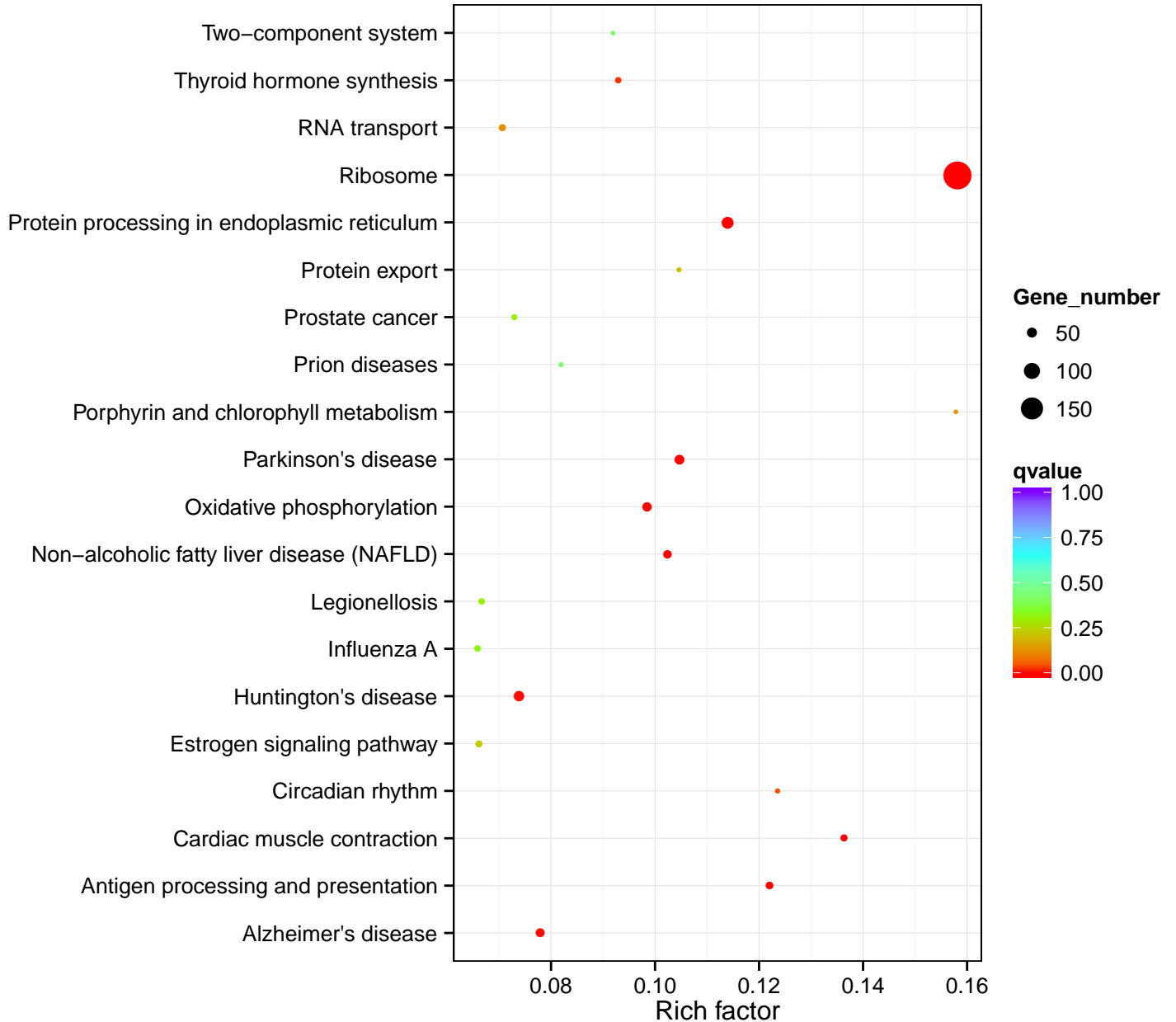

Supplement: Supplemental Material [file supp_g3.116.029314_FigureS3.zip › Figure S2. KEGG enrichment analysis of other developmental comparison groups/C1vsF3_up.DEG_enriched_KEGG_pathway_scatterplot.pdf]

# Statistics of Pathway Enrichment

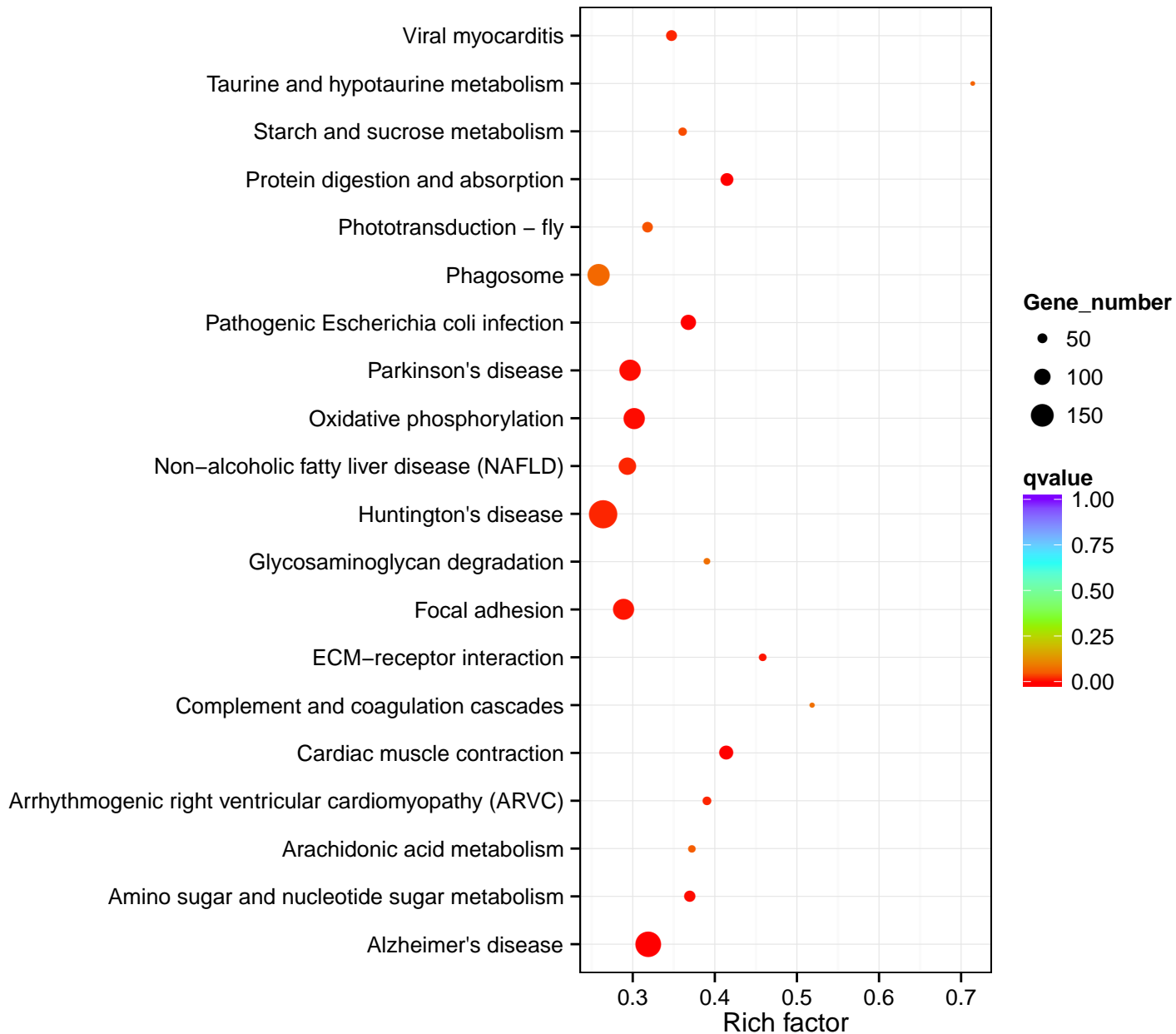

Supplement: Supplemental Material [file supp_g3.116.029314_FigureS3.zip › Figure S2. KEGG enrichment analysis of other developmental comparison groups/C1vsJ4.DEG_enriched_KEGG_pathway_scatterplot.pdf]

# Statistics of Pathway Enrichment

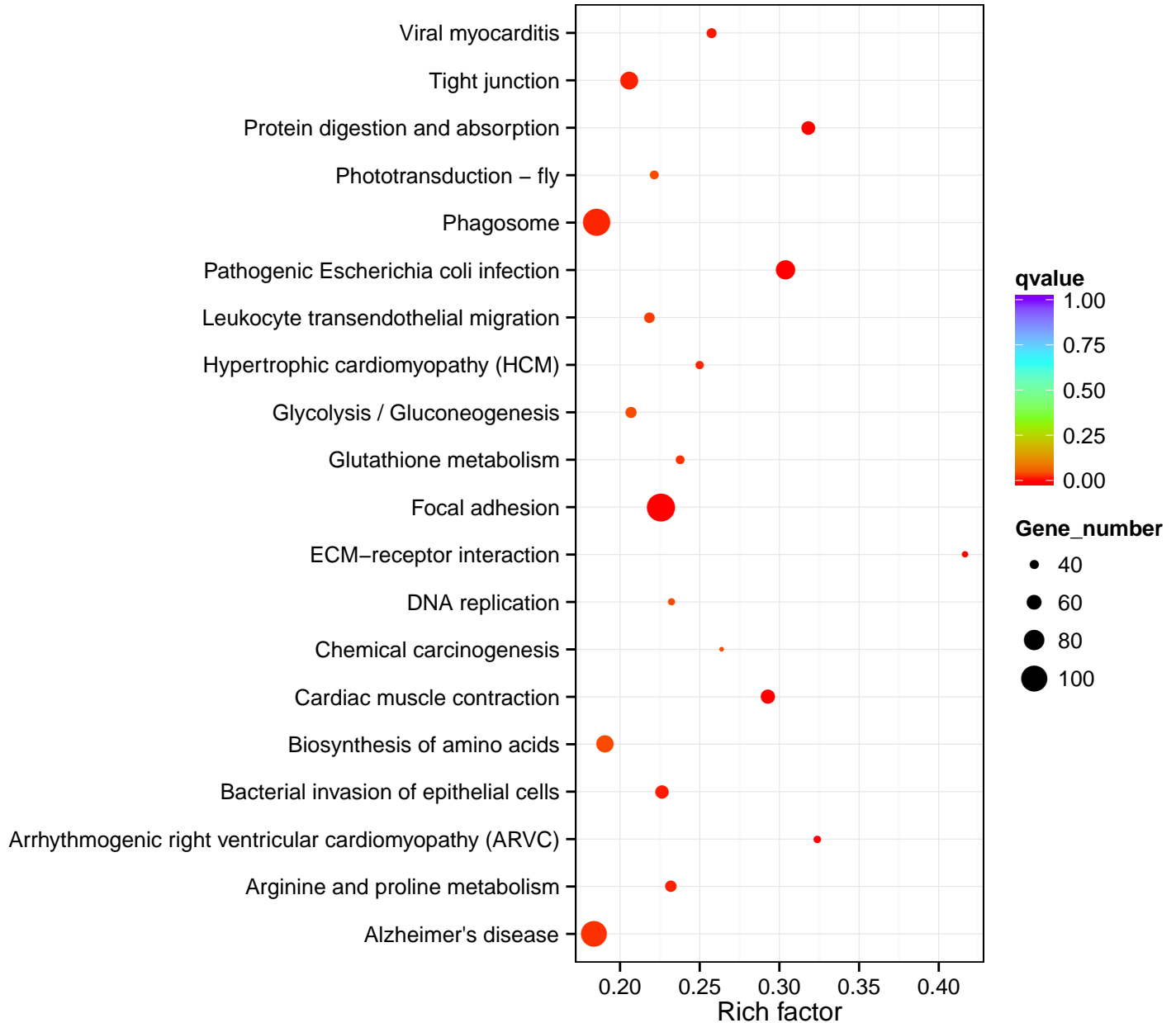

Supplement: Supplemental Material [file supp_g3.116.029314_FigureS3.zip › Figure S2. KEGG enrichment analysis of other developmental comparison groups/C1vsJ4_down.DEG_enriched_KEGG_pathway_scatterplot.pdf]

# Statistics of Pathway Enrichment

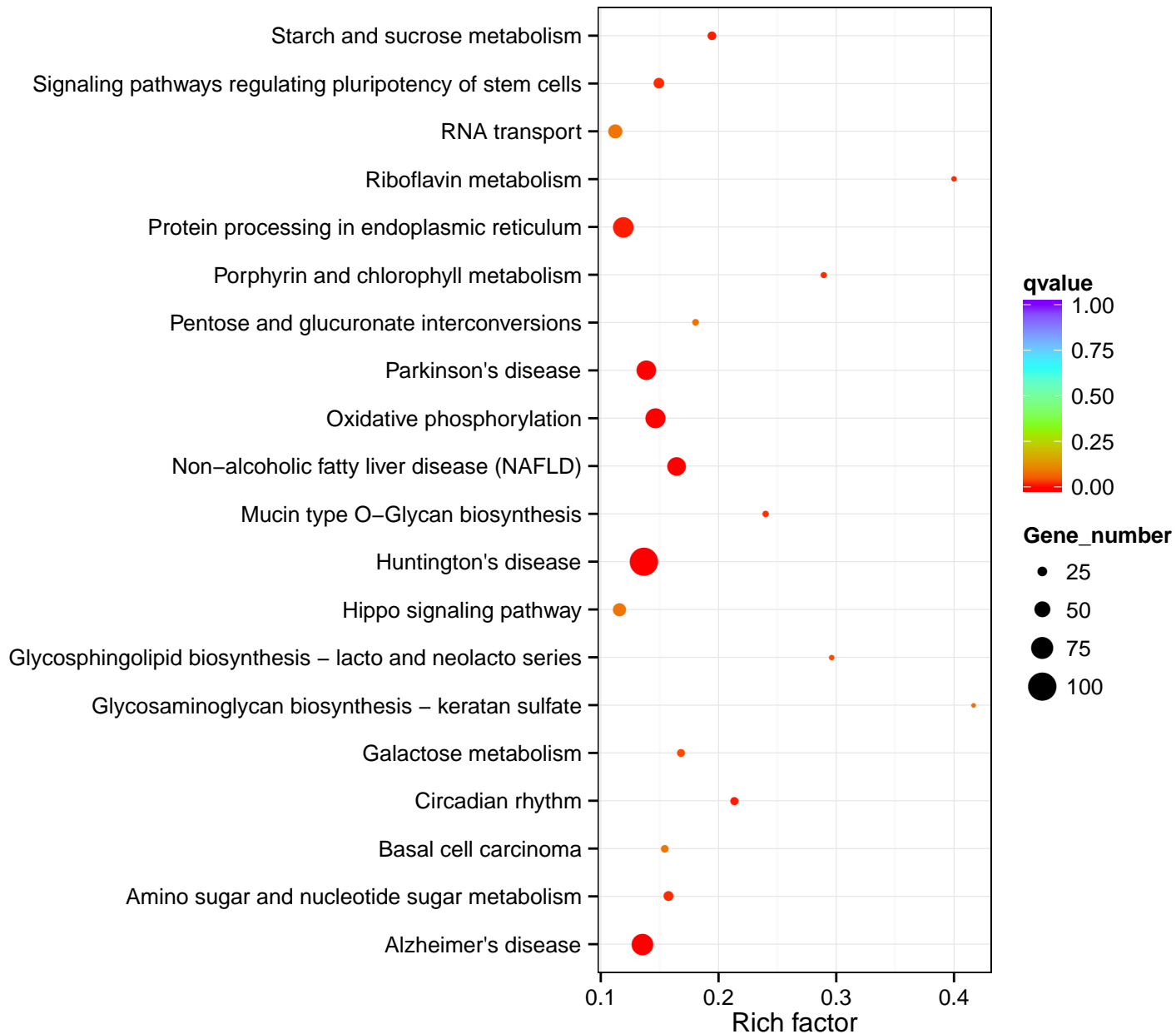

Supplement: Supplemental Material [file supp_g3.116.029314_FigureS3.zip › Figure S2. KEGG enrichment analysis of other developmental comparison groups/C1vsJ4_up.DEG_enriched_KEGG_pathway_scatterplot.pdf]

# Statistics of Pathway Enrichment

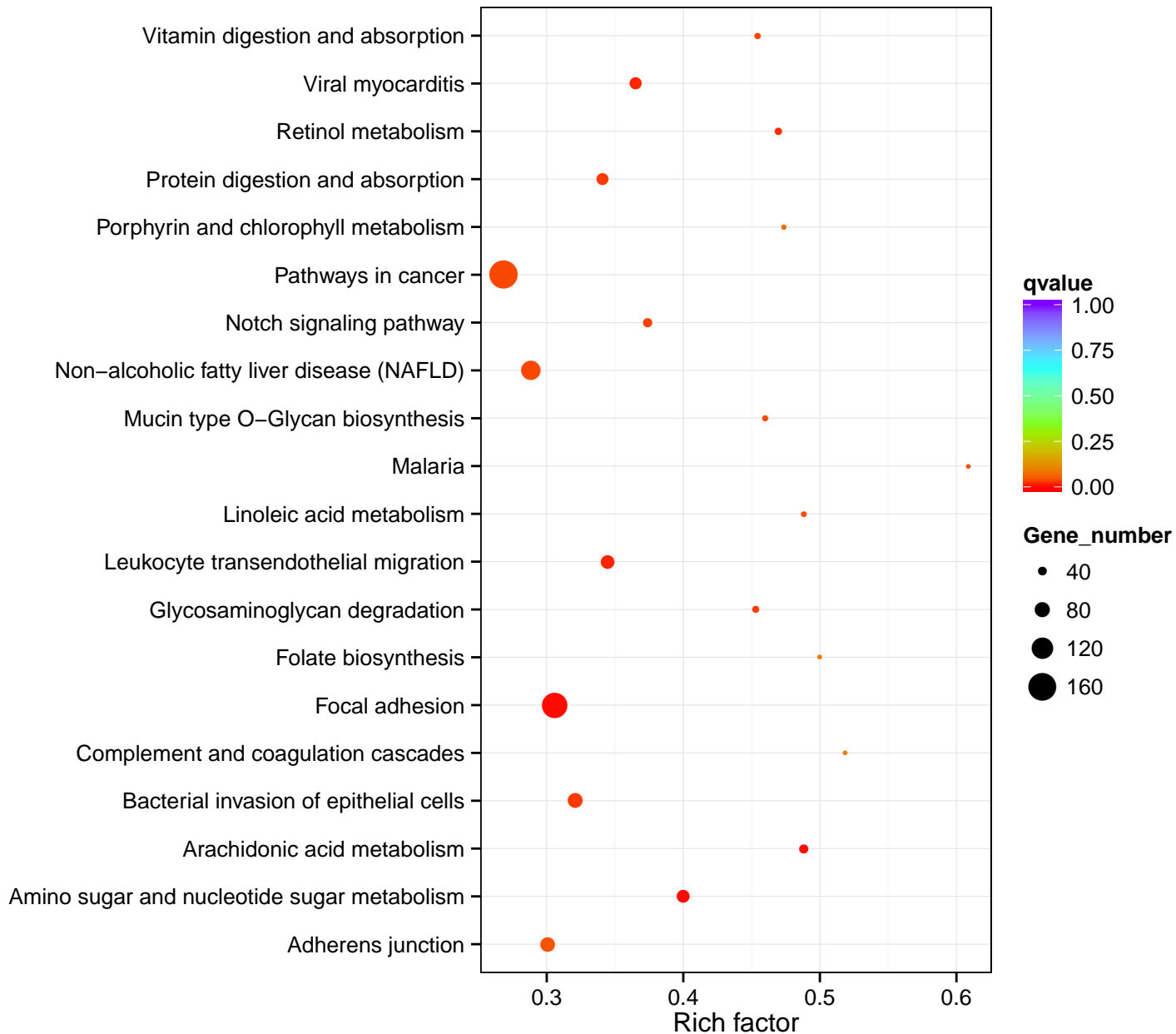

Supplement: Supplemental Material [file supp_g3.116.029314_FigureS3.zip › Figure S2. KEGG enrichment analysis of other developmental comparison groups/C1vsY5.DEG_enriched_KEGG_pathway_scatterplot.pdf]

# Statistics of Pathway Enrichment

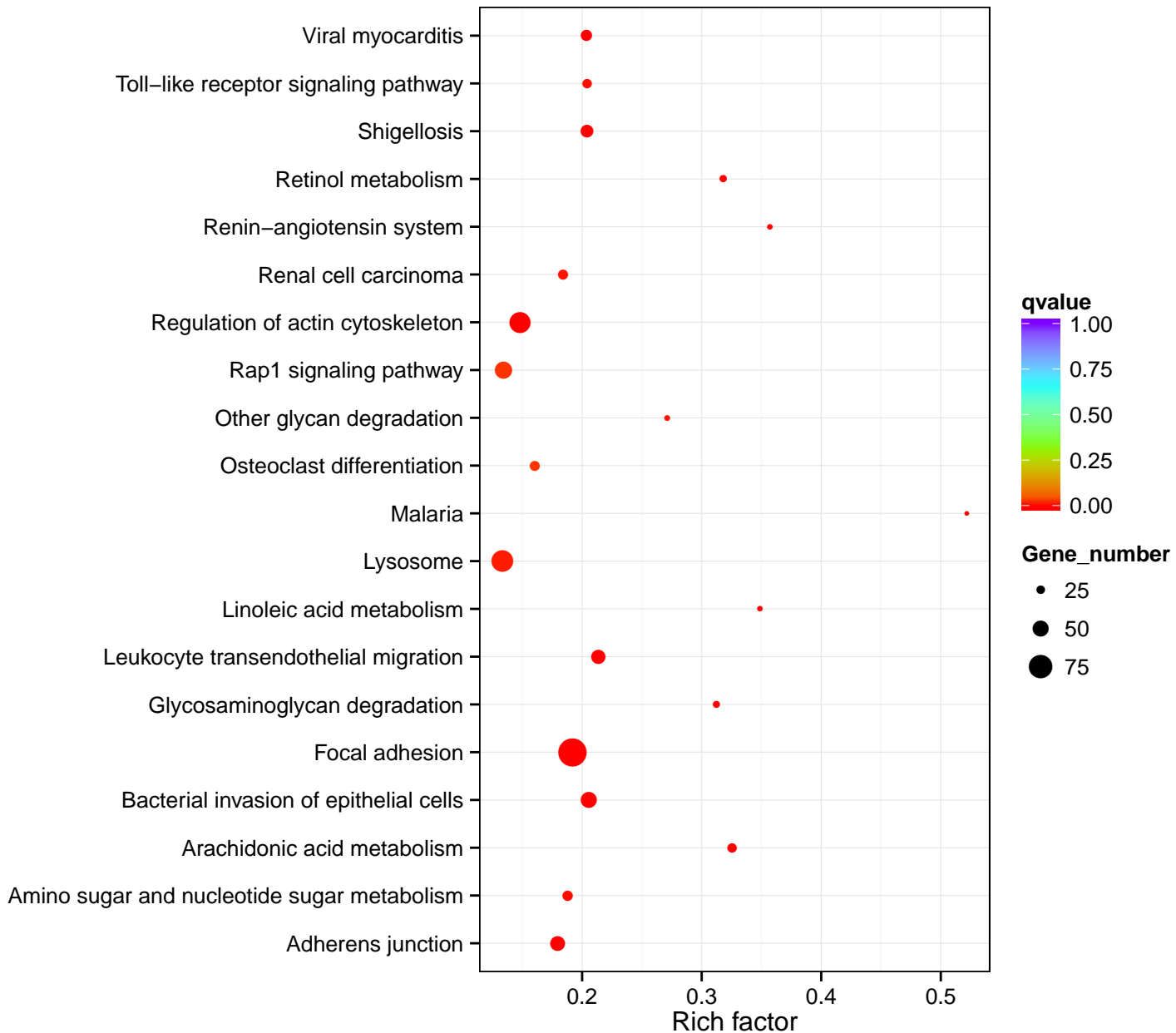

Supplement: Supplemental Material [file supp_g3.116.029314_FigureS3.zip › Figure S2. KEGG enrichment analysis of other developmental comparison groups/C1vsY5_down.DEG_enriched_KEGG_pathway_scatterplot.pdf]

# Statistics of Pathway Enrichment

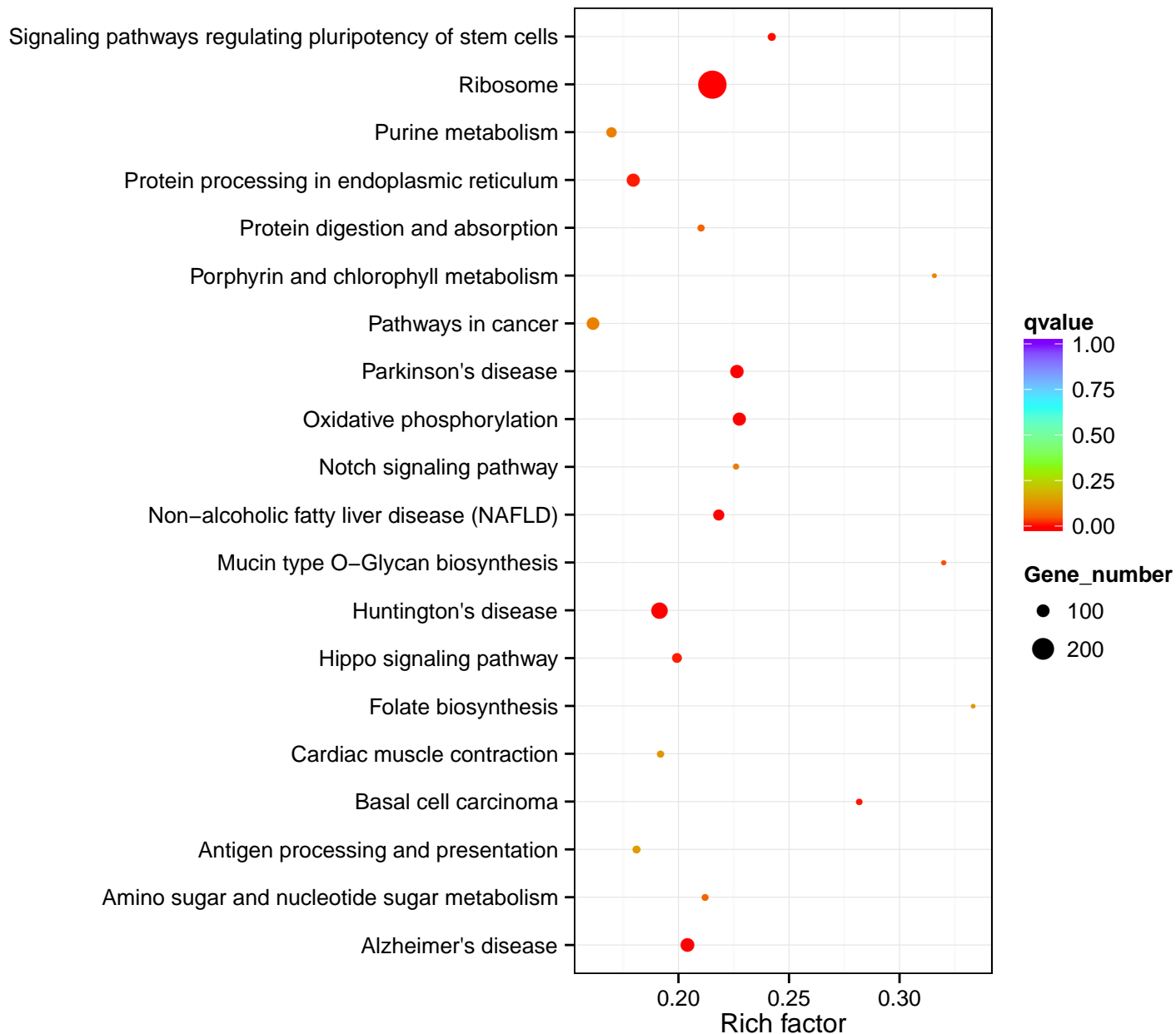

Supplement: Supplemental Material [file supp_g3.116.029314_FigureS3.zip › Figure S2. KEGG enrichment analysis of other developmental comparison groups/C1vsY5_up.DEG_enriched_KEGG_pathway_scatterplot.pdf]

# Statistics of Pathway Enrichment

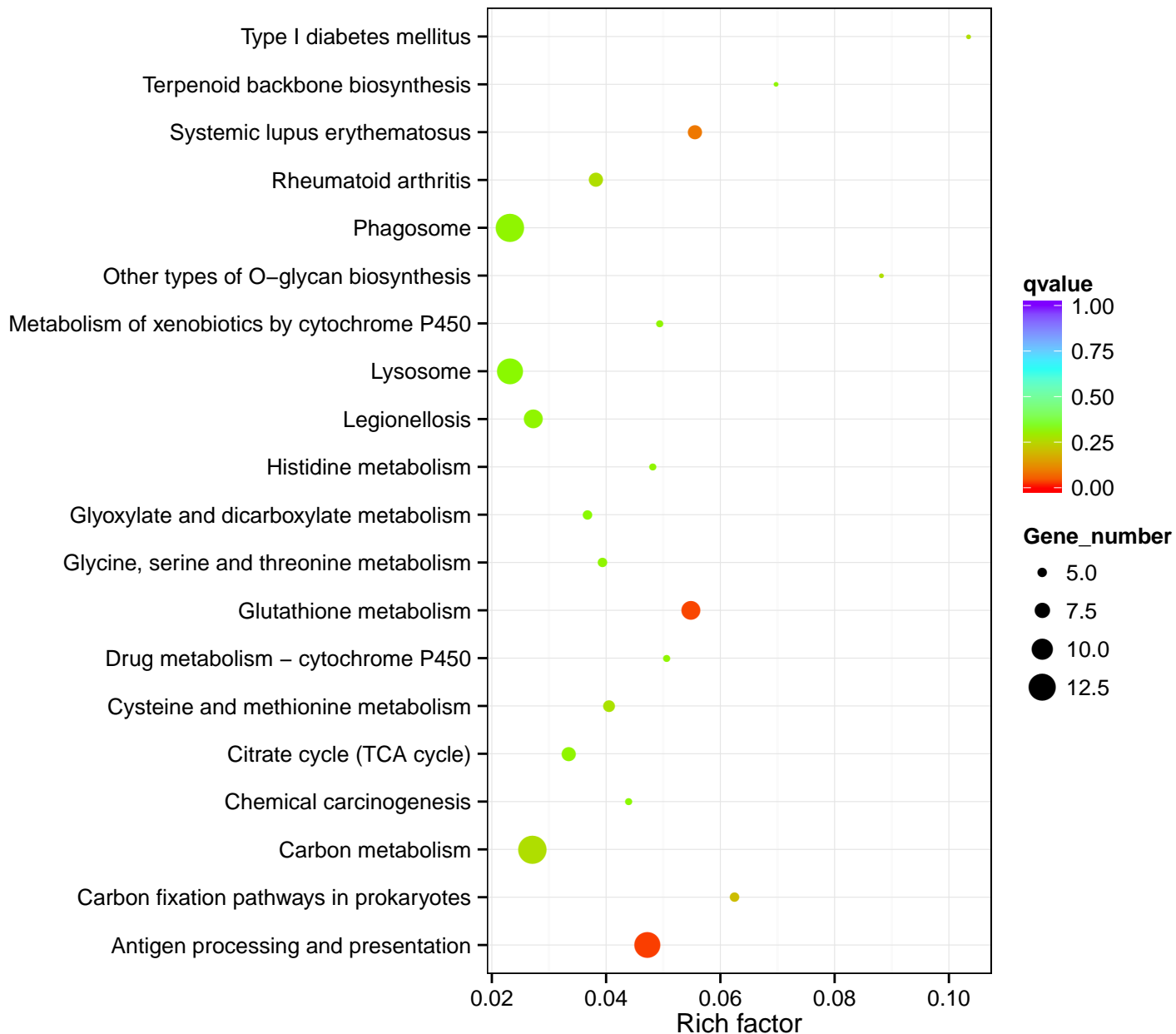

Supplement: Supplemental Material [file supp_g3.116.029314_FigureS3.zip › Figure S2. KEGG enrichment analysis of other developmental comparison groups/D2vsF3.DEG_enriched_KEGG_pathway_scatterplot.pdf]

# Statistics of Pathway Enrichment

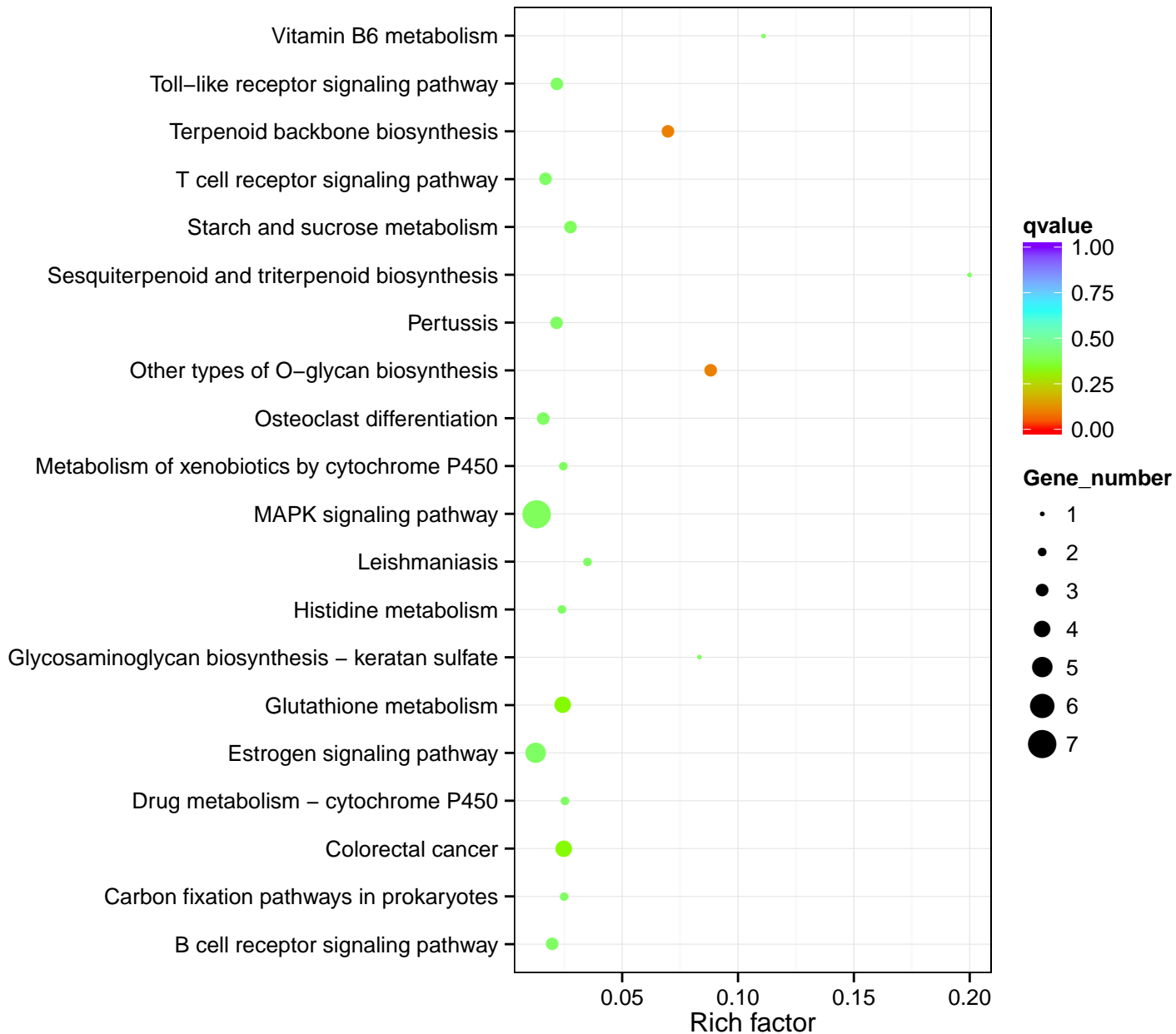

Supplement: Supplemental Material [file supp_g3.116.029314_FigureS3.zip › Figure S2. KEGG enrichment analysis of other developmental comparison groups/D2vsF3_down.DEG_enriched_KEGG_pathway_scatterplot.pdf]

# Statistics of Pathway Enrichment

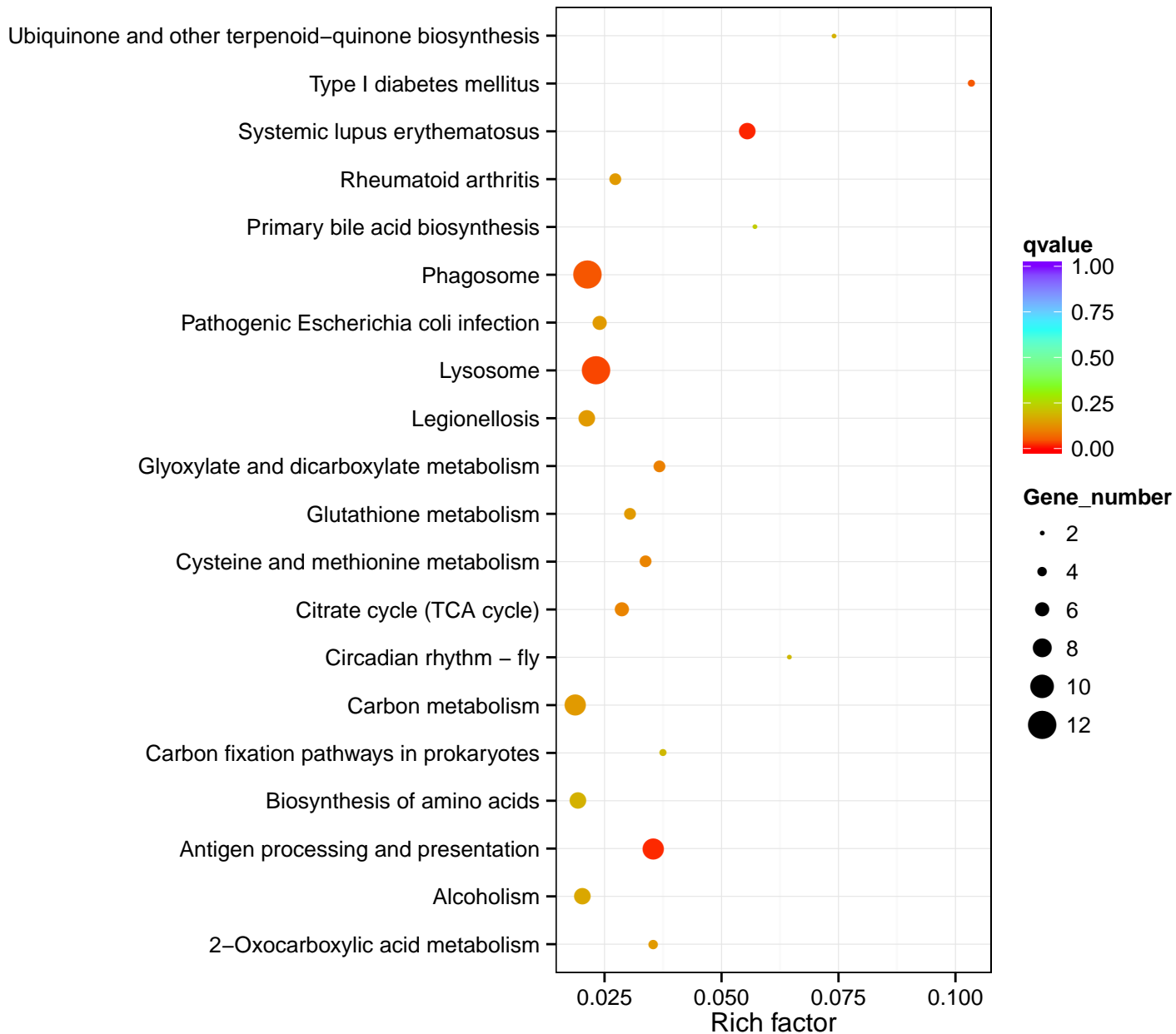

Supplement: Supplemental Material [file supp_g3.116.029314_FigureS3.zip › Figure S2. KEGG enrichment analysis of other developmental comparison groups/D2vsF3_up.DEG_enriched_KEGG_pathway_scatterplot.pdf]

# Statistics of Pathway Enrichment

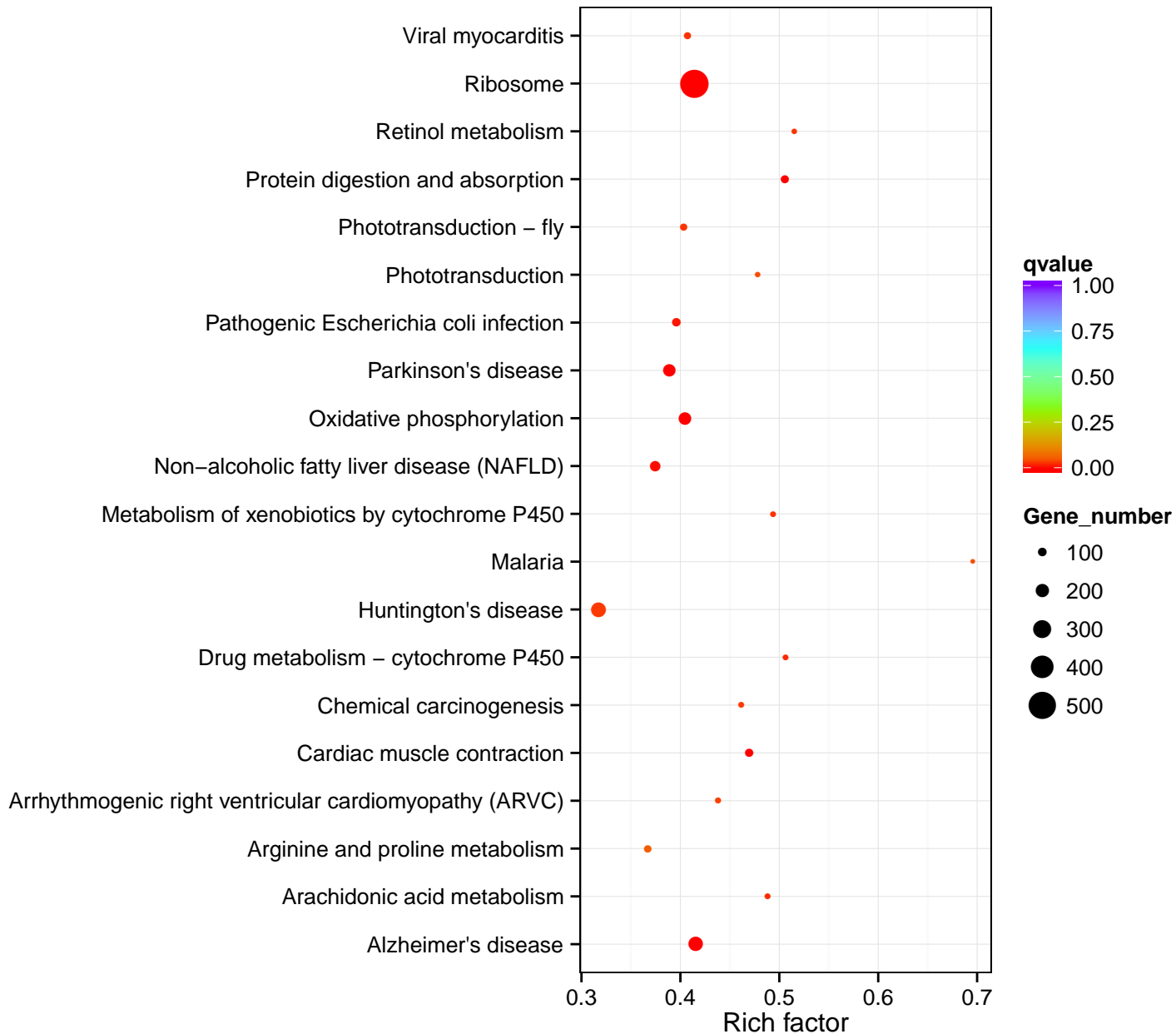

Supplement: Supplemental Material [file supp_g3.116.029314_FigureS3.zip › Figure S2. KEGG enrichment analysis of other developmental comparison groups/D2vsJ4.DEG_enriched_KEGG_pathway_scatterplot.pdf]

# Statistics of Pathway Enrichment

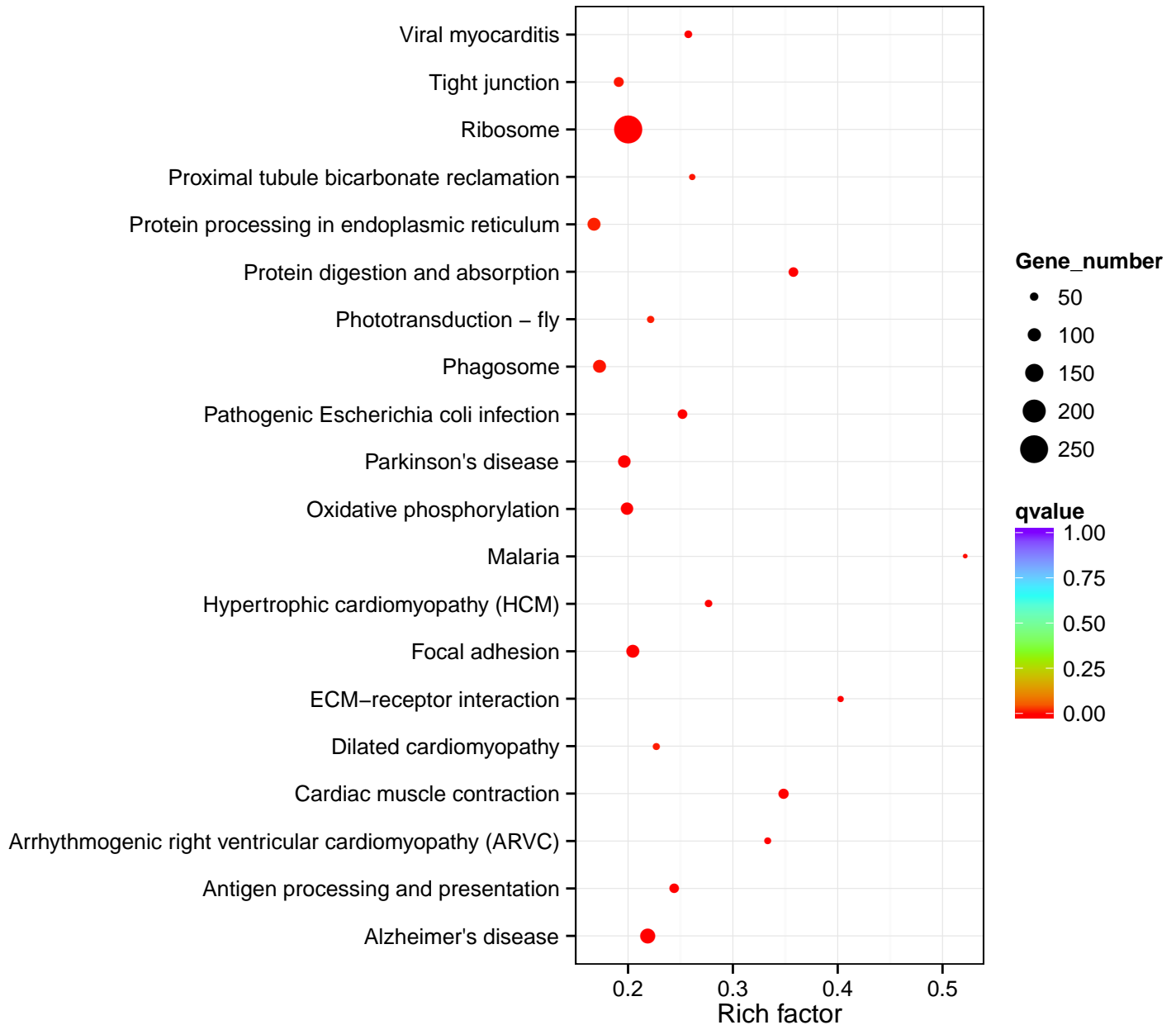

Supplement: Supplemental Material [file supp_g3.116.029314_FigureS3.zip › Figure S2. KEGG enrichment analysis of other developmental comparison groups/D2vsJ4_down.DEG_enriched_KEGG_pathway_scatterplot.pdf]

# Statistics of Pathway Enrichment

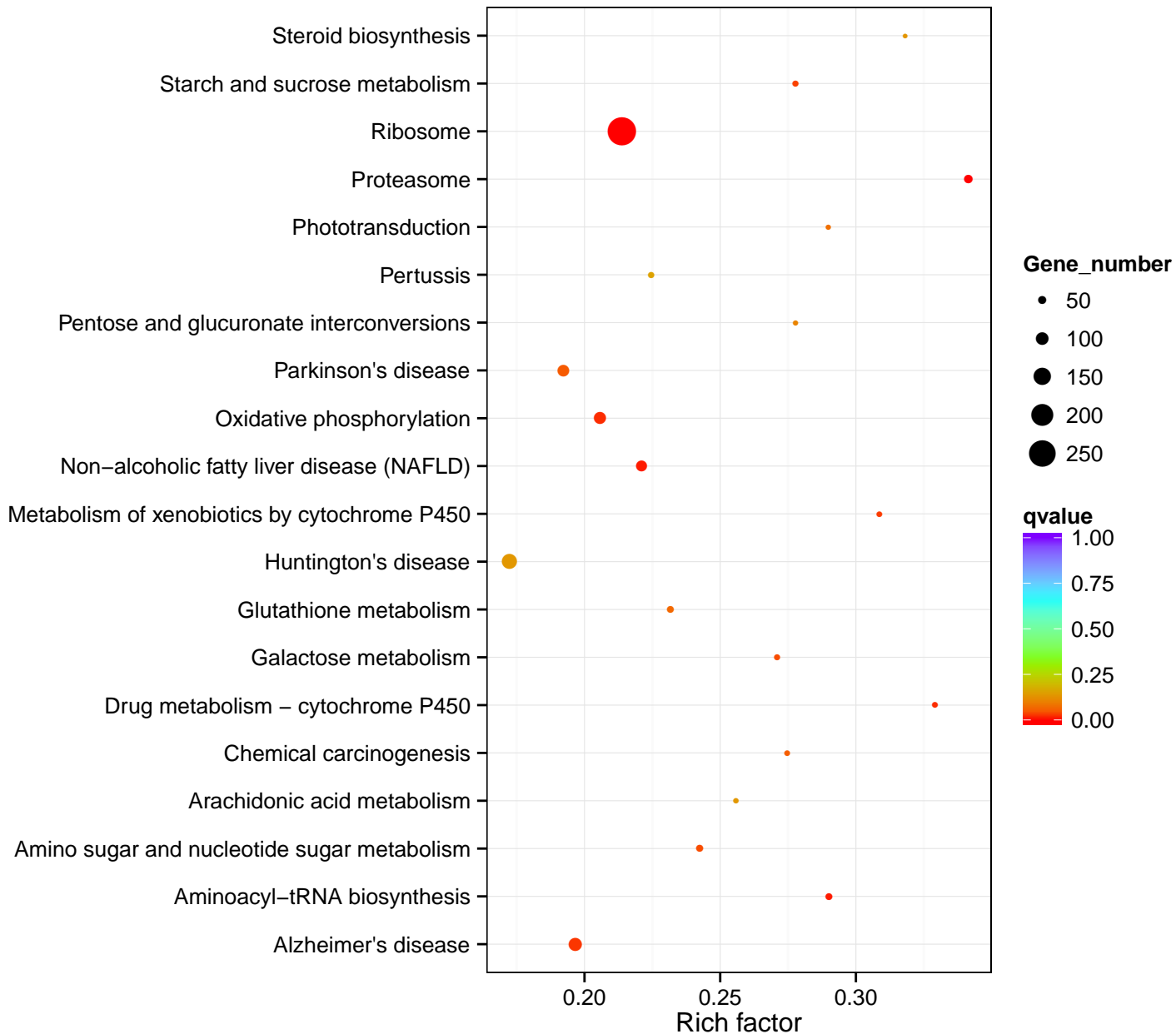

Supplement: Supplemental Material [file supp_g3.116.029314_FigureS3.zip › Figure S2. KEGG enrichment analysis of other developmental comparison groups/D2vsJ4_up.DEG_enriched_KEGG_pathway_scatterplot.pdf]

# Statistics of Pathway Enrichment

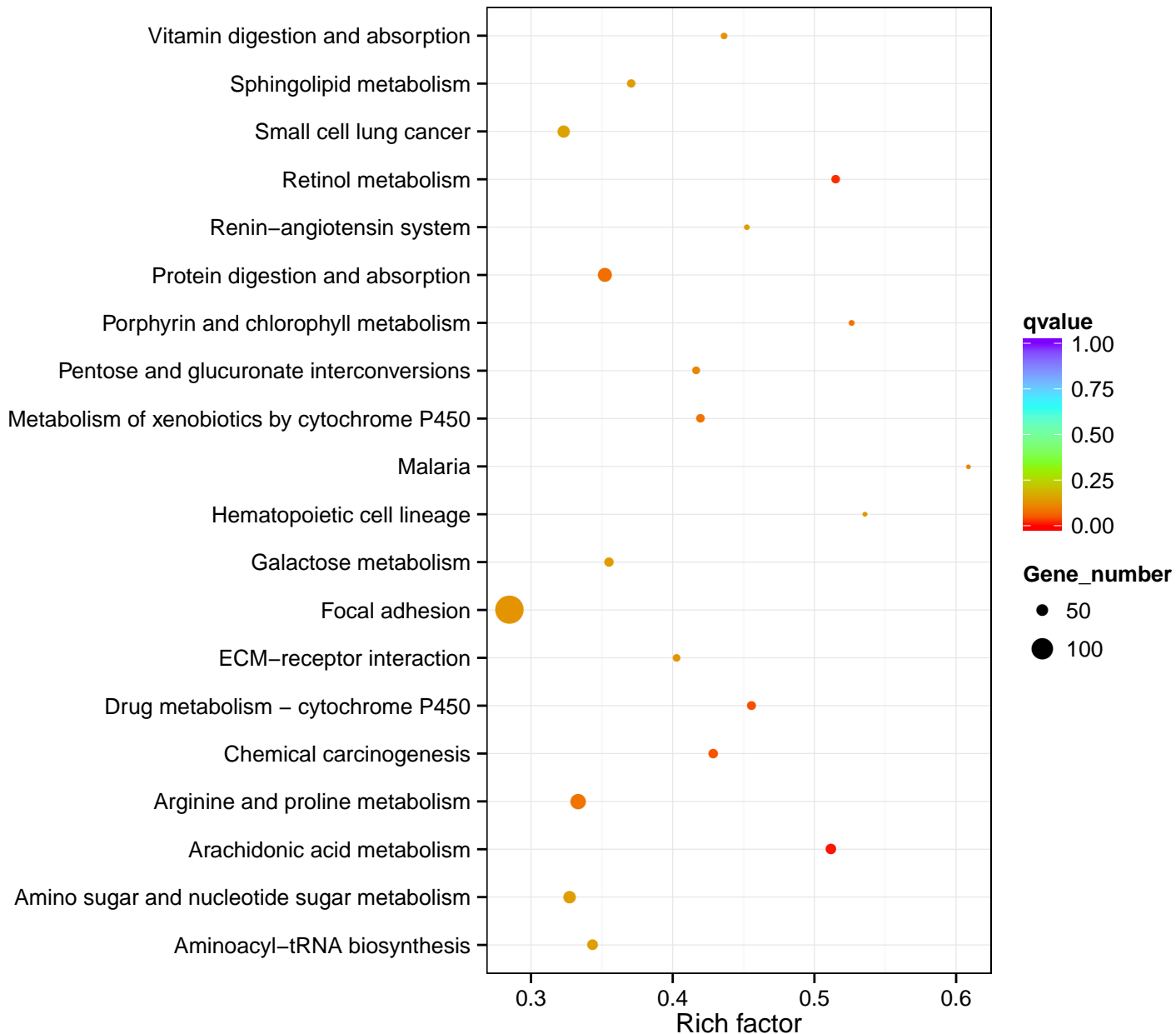

Supplement: Supplemental Material [file supp_g3.116.029314_FigureS3.zip › Figure S2. KEGG enrichment analysis of other developmental comparison groups/D2vsY5.DEG_enriched_KEGG_pathway_scatterplot.pdf]

# Statistics of Pathway Enrichment

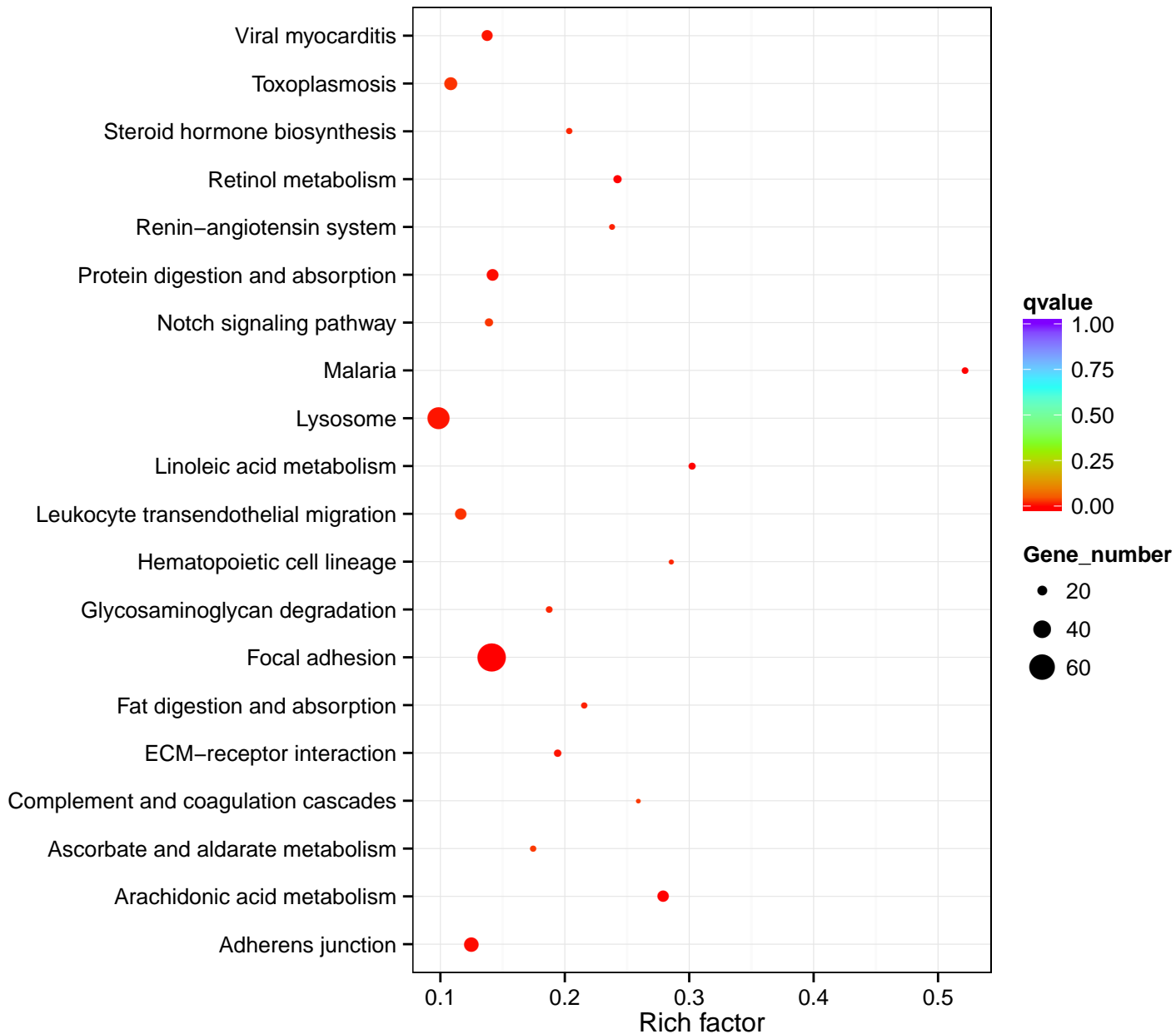

Supplement: Supplemental Material [file supp_g3.116.029314_FigureS3.zip › Figure S2. KEGG enrichment analysis of other developmental comparison groups/D2vsY5_down.DEG_enriched_KEGG_pathway_scatterplot.pdf]

# Statistics of Pathway Enrichment

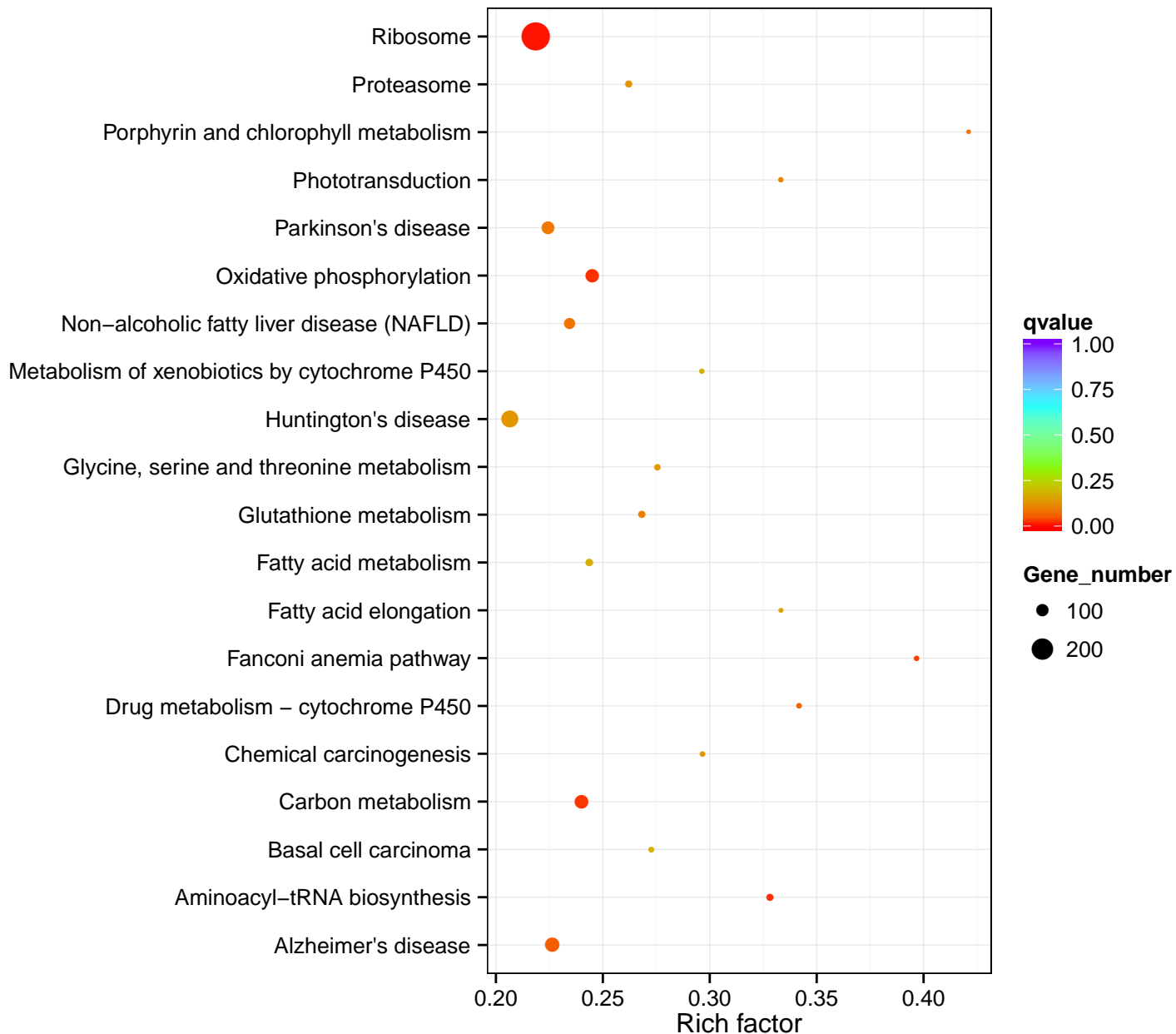

Supplement: Supplemental Material [file supp_g3.116.029314_FigureS3.zip › Figure S2. KEGG enrichment analysis of other developmental comparison groups/D2vsY5_up.DEG_enriched_KEGG_pathway_scatterplot.pdf]

# Statistics of Pathway Enrichment

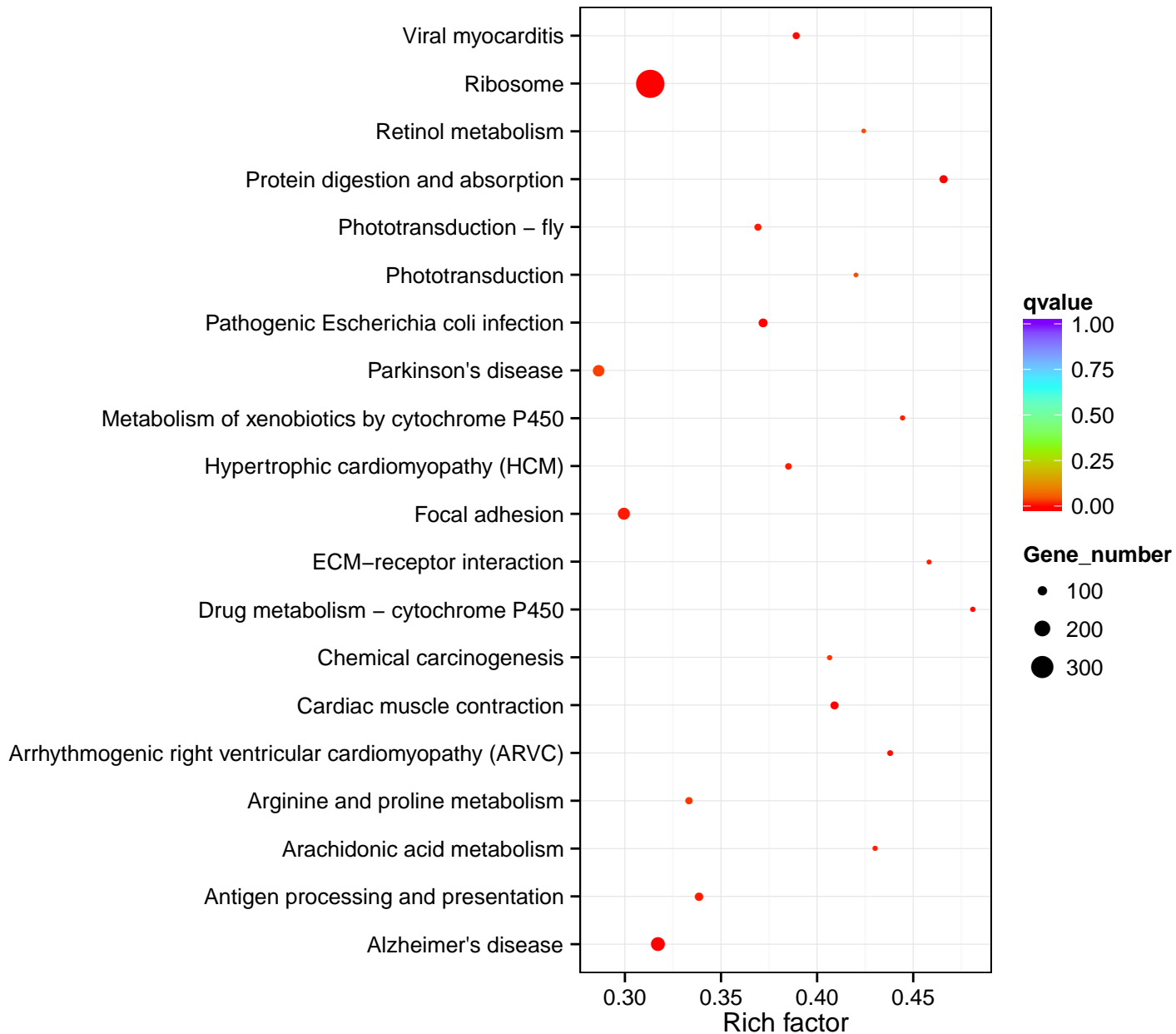

Supplement: Supplemental Material [file supp_g3.116.029314_FigureS3.zip › Figure S2. KEGG enrichment analysis of other developmental comparison groups/F3vsJ4.DEG_enriched_KEGG_pathway_scatterplot.pdf]

# Statistics of Pathway Enrichment

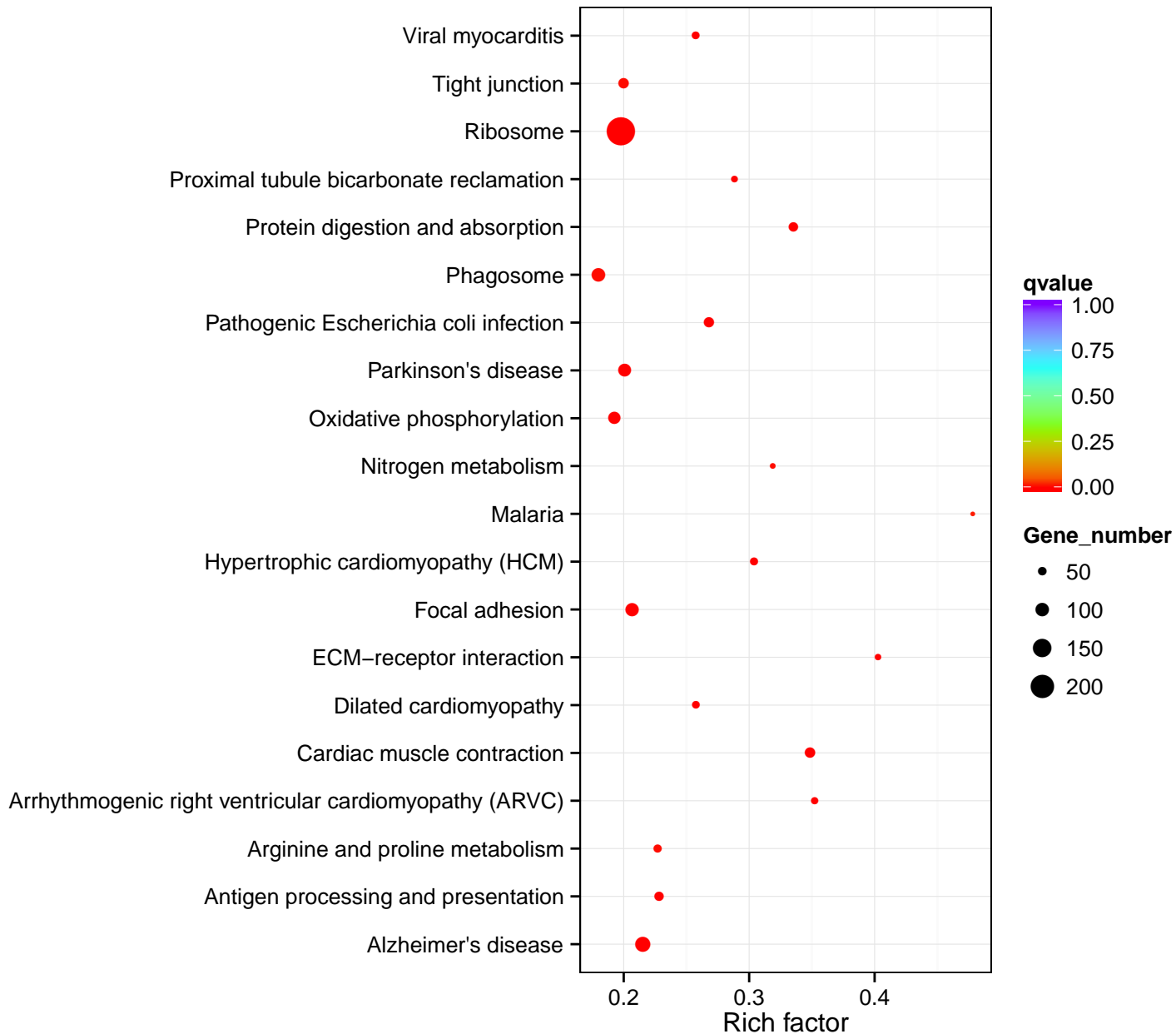

Supplement: Supplemental Material [file supp_g3.116.029314_FigureS3.zip › Figure S2. KEGG enrichment analysis of other developmental comparison groups/F3vsJ4_down.DEG_enriched_KEGG_pathway_scatterplot.pdf]

# Statistics of Pathway Enrichment

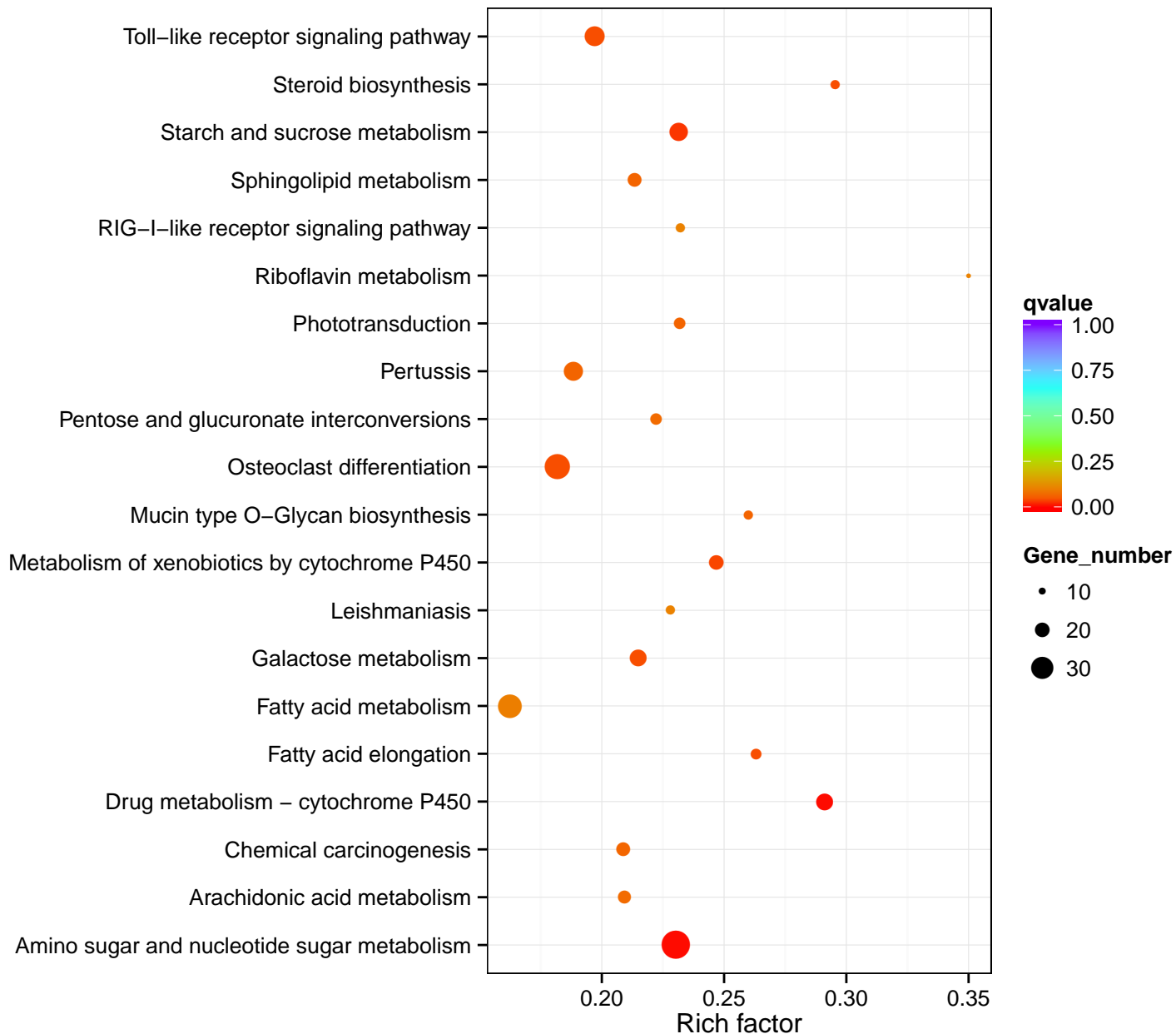

Supplement: Supplemental Material [file supp_g3.116.029314_FigureS3.zip › Figure S2. KEGG enrichment analysis of other developmental comparison groups/F3vsJ4_up.DEG_enriched_KEGG_pathway_scatterplot.pdf]

# Statistics of Pathway Enrichment

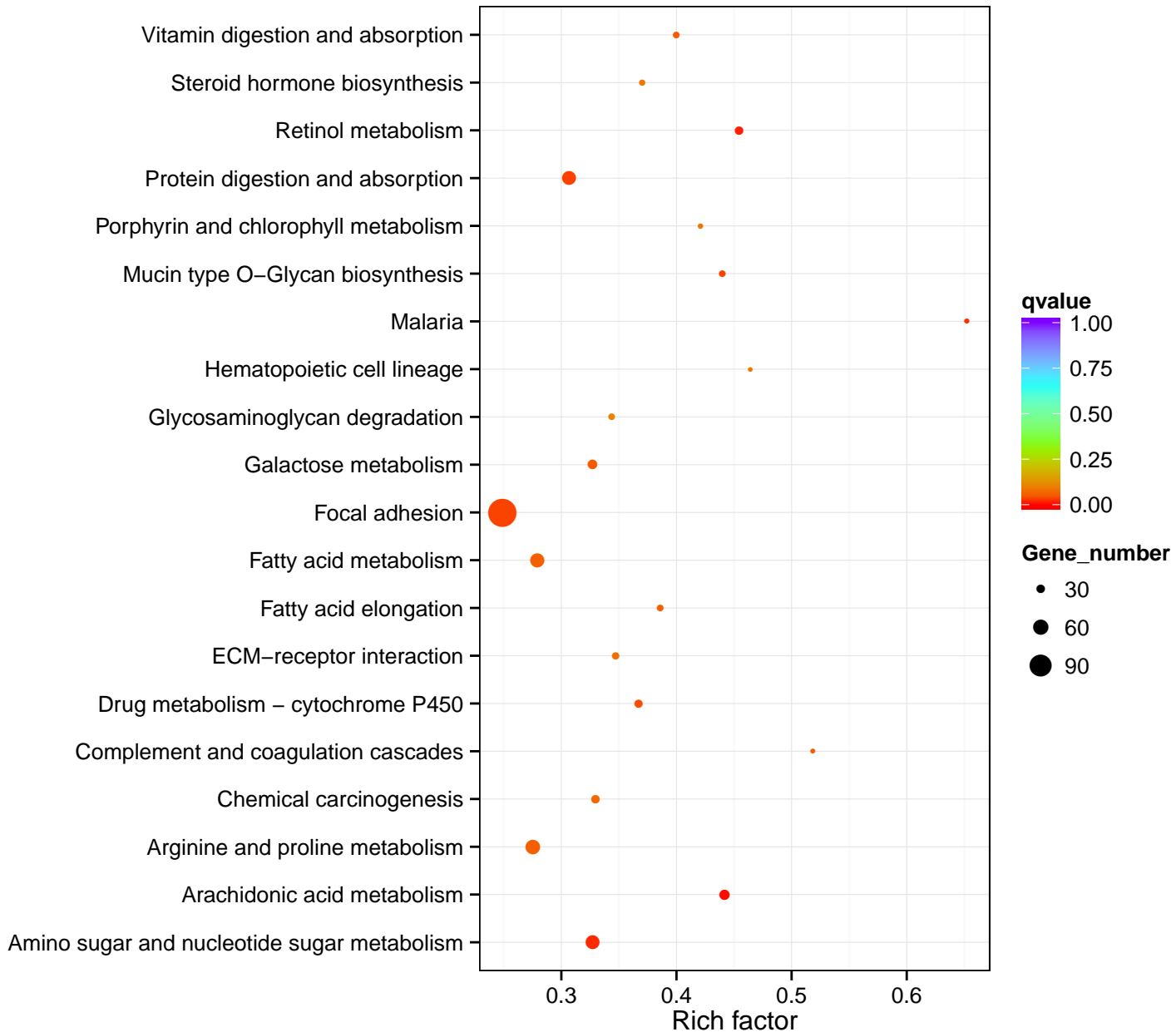

Supplement: Supplemental Material [file supp_g3.116.029314_FigureS3.zip › Figure S2. KEGG enrichment analysis of other developmental comparison groups/F3vsY5.DEG_enriched_KEGG_pathway_scatterplot.pdf]

# Statistics of Pathway Enrichment

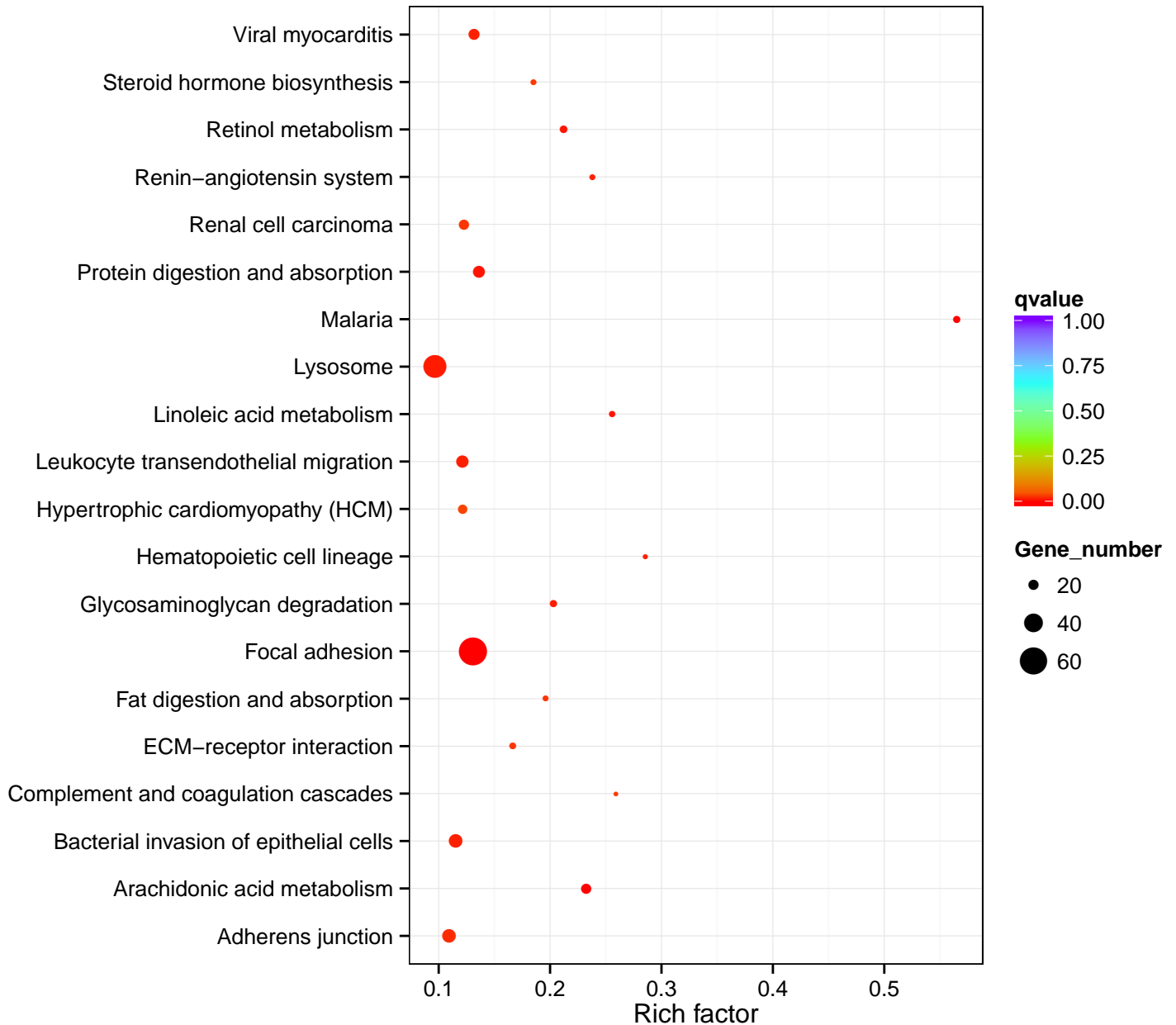

Supplement: Supplemental Material [file supp_g3.116.029314_FigureS3.zip › Figure S2. KEGG enrichment analysis of other developmental comparison groups/F3vsY5_down.DEG_enriched_KEGG_pathway_scatterplot.pdf]

# Statistics of Pathway Enrichment

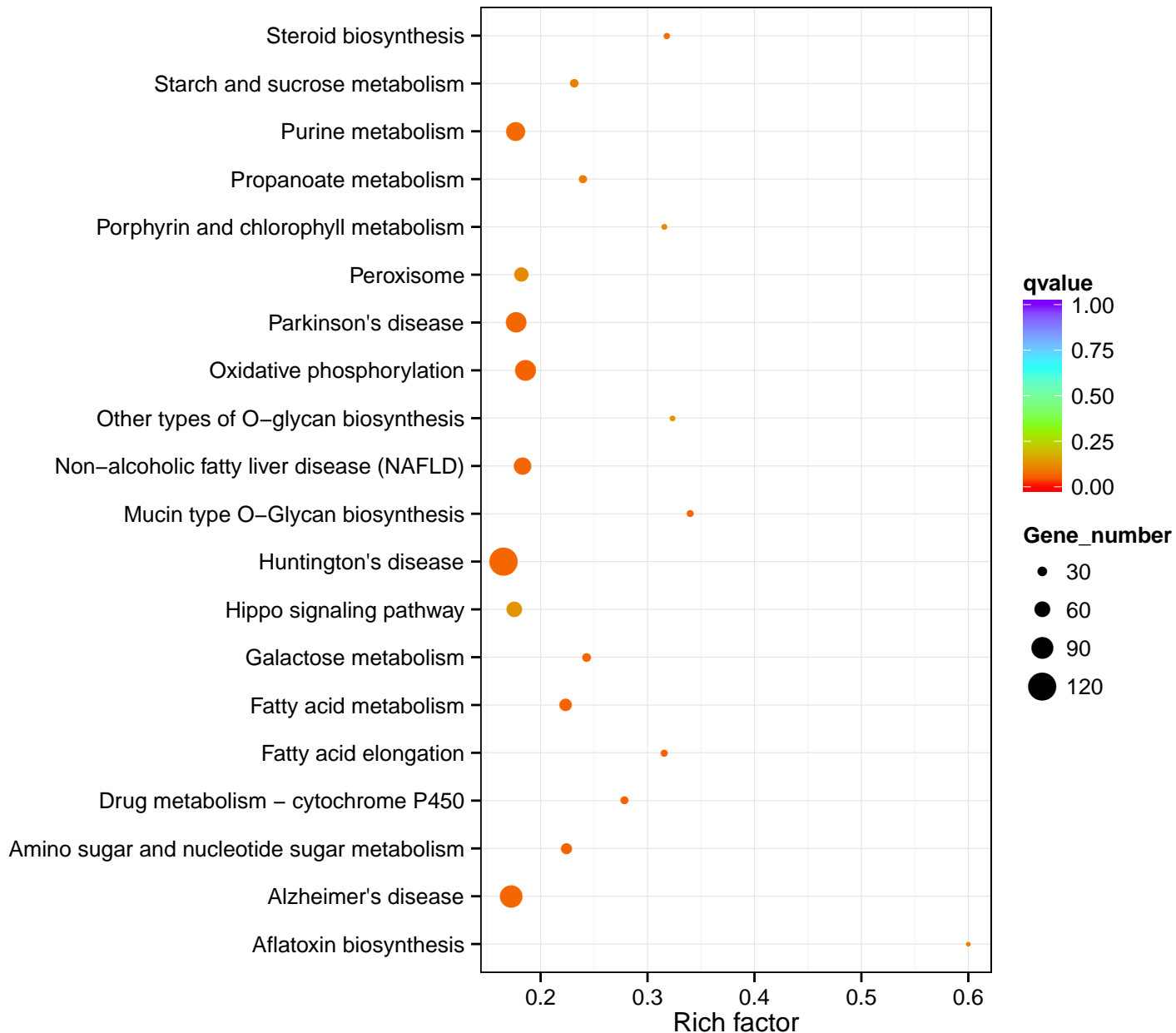

Supplement: Supplemental Material [file supp_g3.116.029314_FigureS3.zip › Figure S2. KEGG enrichment analysis of other developmental comparison groups/F3vsY5_up.DEG_enriched_KEGG_pathway_scatterplot.pdf]

# Statistics of Pathway Enrichment

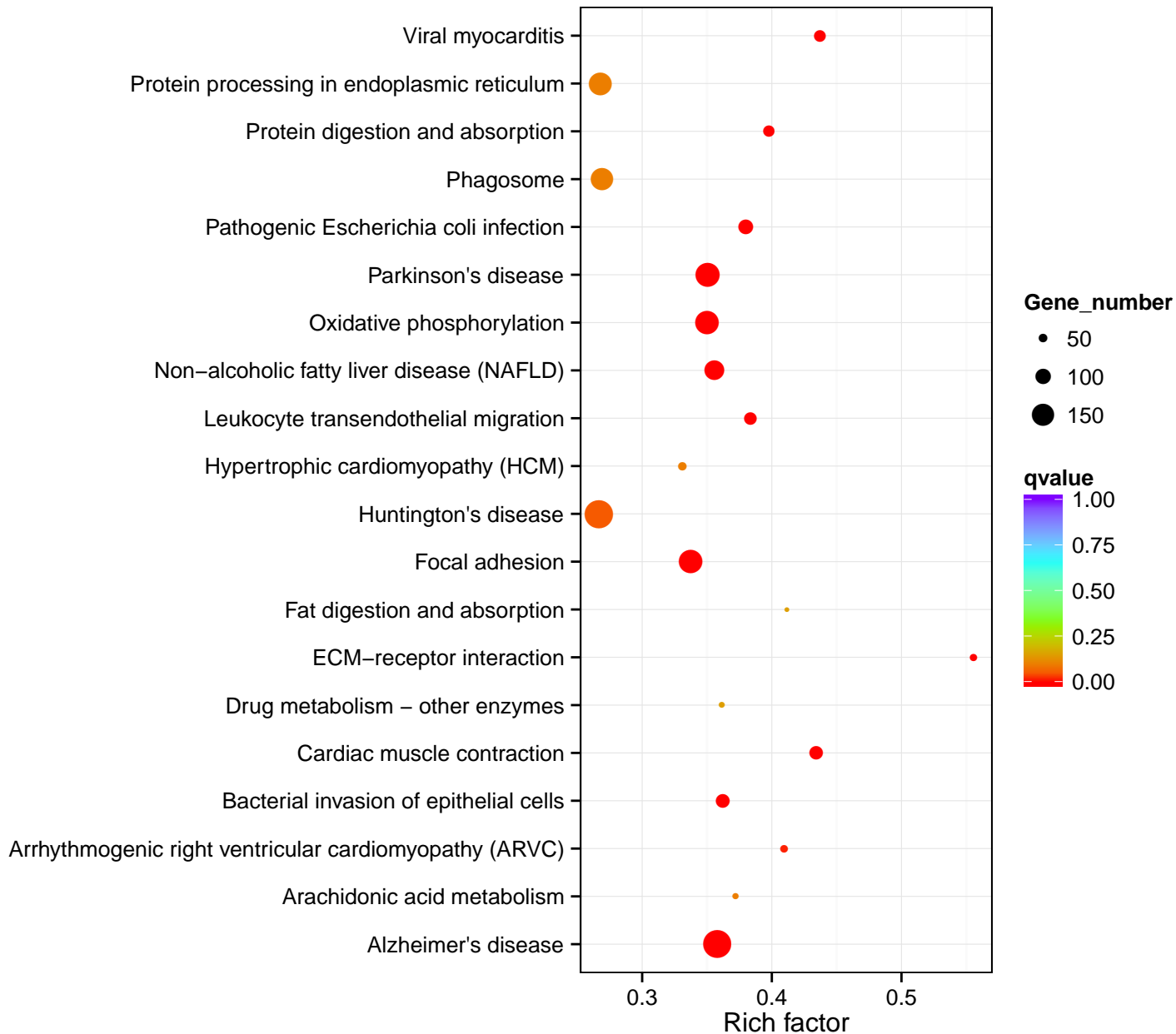

Supplement: Supplemental Material [file supp_g3.116.029314_FigureS3.zip › Figure S2. KEGG enrichment analysis of other developmental comparison groups/J4vsY5.DEG_enriched_KEGG_pathway_scatterplot.pdf]

# Statistics of Pathway Enrichment

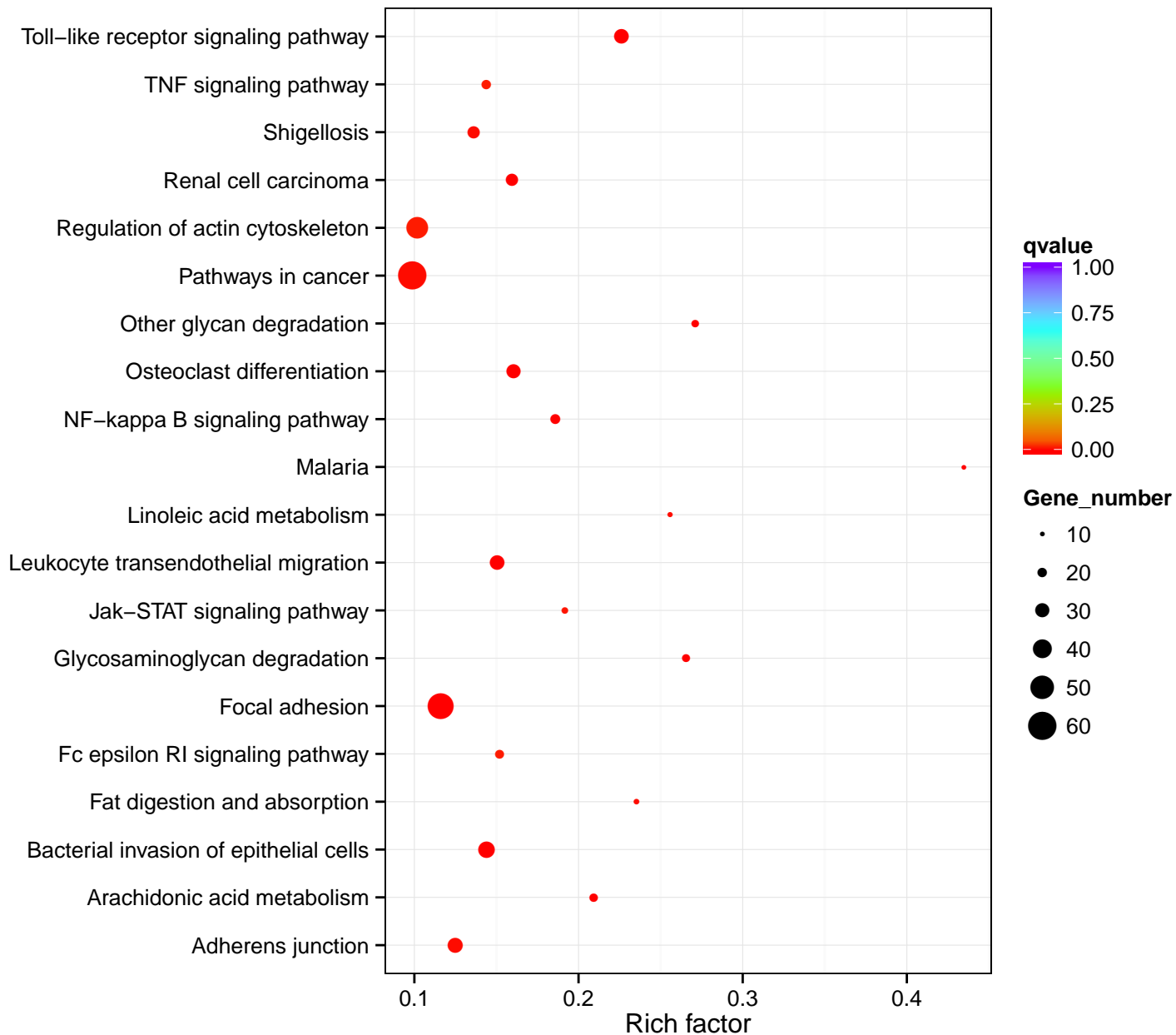

Supplement: Supplemental Material [file supp_g3.116.029314_FigureS3.zip › Figure S2. KEGG enrichment analysis of other developmental comparison groups/J4vsY5_down.DEG_enriched_KEGG_pathway_scatterplot.pdf]

# Statistics of Pathway Enrichment

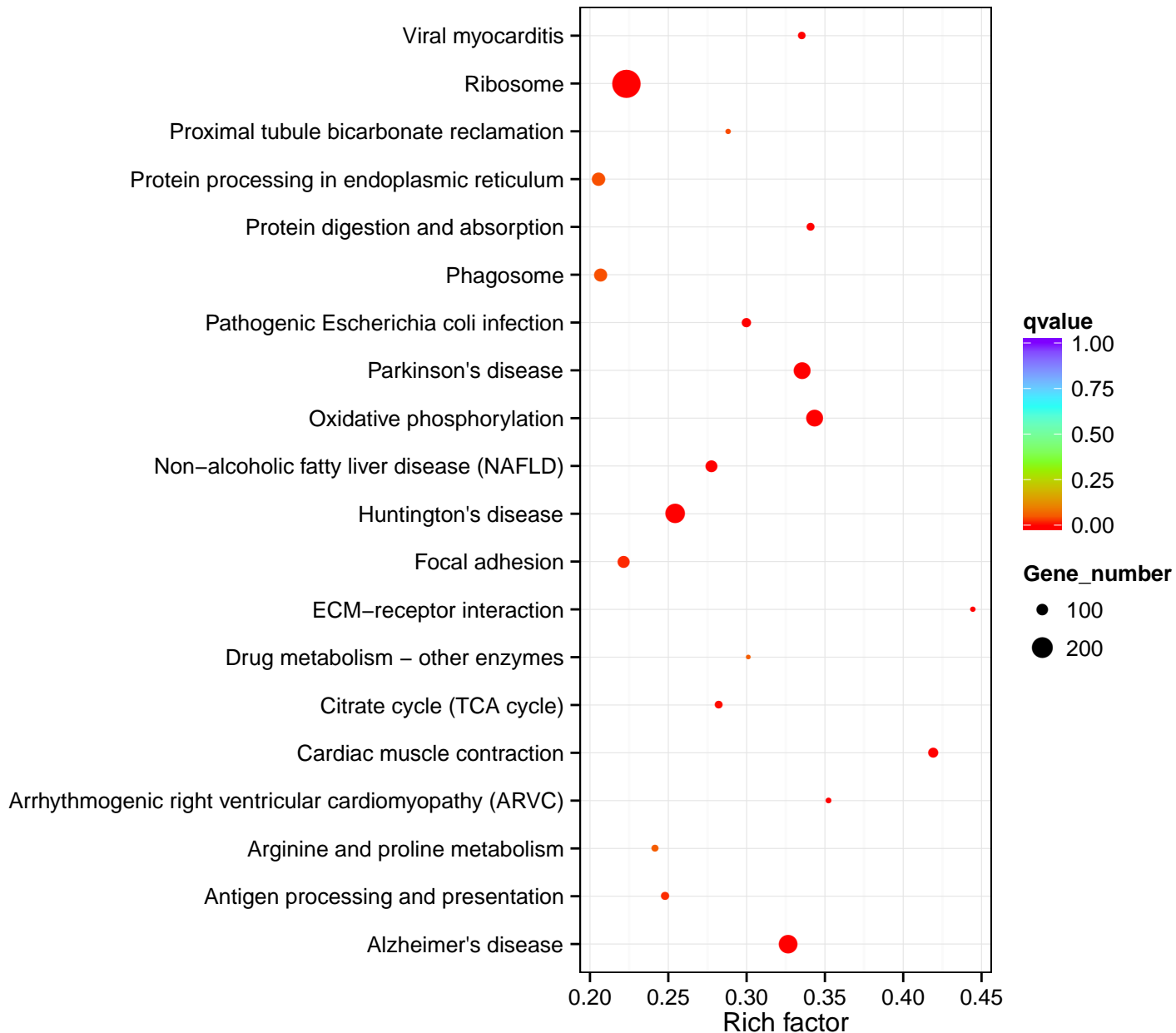

Supplement: Supplemental Material [file supp_g3.116.029314_FigureS3.zip › Figure S2. KEGG enrichment analysis of other developmental comparison groups/J4vsY5_up.DEG_enriched_KEGG_pathway_scatterplot.pdf]
